# Self-Assembled Capsules of Unprecedented Shapes**

**DOI:** 10.1002/anie.201102548

**Published:** 2011-08-26

**Authors:** Konrad Tiefenbacher, Dariush Ajami, Julius Rebek

**Affiliations:** The Skaggs Institute for Chemical Biology and Department of Chemistry, The Scripps Research Institute10550 North Torrey Pines Road, La Jolla, CA 92037 (USA)

**Keywords:** host–guest systems, molecular capsules, molecular recognition, reversible encapsulation, self-assembly

Reversible encapsulation allows the temporary isolation of molecules in very small spaces. There, molecular behavior is quite different than that in bulk solvent; in capsules recognition can be amplified,[1] reactive intermediates can be stabilized,[2] reactions can be accelerated[3] or even catalyzed[4] and new reaction pathways can appear.[5] Accordingly, a multitude of capsules have been devised over the last two decades. The main cohesive forces—covalent bonds,[6] hydrogen bonds[7] metals and ligands,[2a–c, 4c, 8] or simple hydrophobic effects[9]—are used to hold the capsules together and a variety of structures are available. Yet the self-assembly processes by their very nature of incorporating multiple subunits tend to create cavity shapes of high symmetry such as spheres, polyhedra and cylinders.

We report here examples of new capsules featuring “S”- and “banana”-shapes that arise from insertion of propanediureas **3** into cylindrical capsule **1.1** (Figure 1 a). An extensive and mutual induced fit behavior is displayed by these systems.

**Figure 1:**
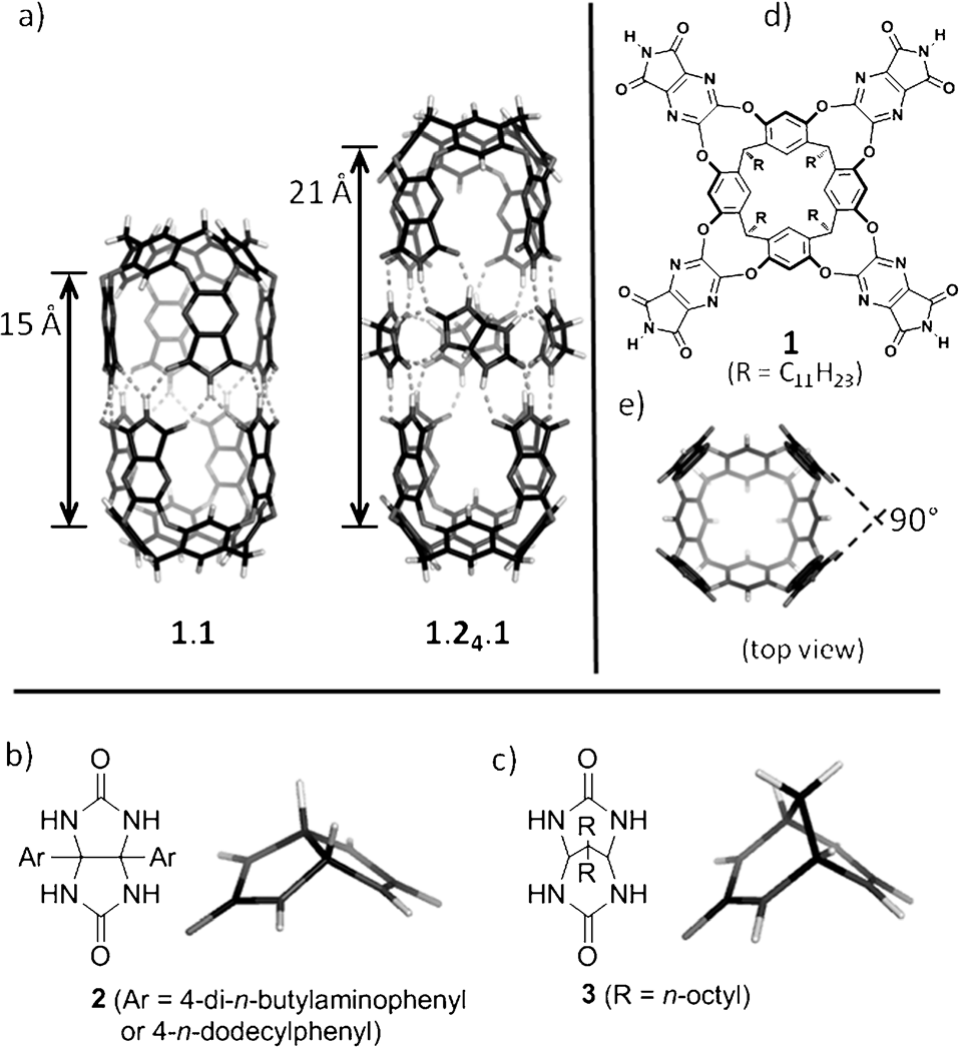
a) Models of the known cylindrical capsules **1.1** and **1.2_4_.1**. b) Structure and model of glycoluril **2**. c) Structure and model of propanediurea **3** used for the studies described herein. d) Structure of cavitand **1**. e) Model of cavitand **1**. (Peripheral alkyl and aryl groups have been deleted for easier viewing.)

The cylindrical capsule host **1.1** (Figure 1 a) spontaneously assembles around appropriate guests in apolar organic solvents such as mesitylene.[10] The complex is held together through a seam of bifurcated hydrogen bonds and attractive forces between guest and host. When glycoluril **2** (Figure 1 b) is present, new assemblies emerge: the glycolurils act as spacer elements[11] that increase the length and capacity of the inner space. The glycolurils offer superior hydrogen bond acceptors to the imides’ NH donors and four glycolurils integrate into the middle of the capsule in a twisted “belt” arrangement that results in a chiral assembly **1.2_4_.1**. The elongation of **1.1** with **2** is not limited to a single belt: longer guests can drive the assembly toward further extension with 2, 3 or 4 belts of glycoluril spacers incorporated.[12] The twisted belt arrangement is apparently due to a geometric mismatch between the adjacent walls of cavitand **1**, that are at right angles to each other (Figure 1 e), and the ureido functions of **2** (Figure 1 b) that are presented at the considerably larger angle of approximately 113°.[13] The corresponding angle of the propanediurea **3** (Figure 1 c) is smaller (ca. 99°)[14] and more appropriate as a complement to the right angles of the cavitand. Accordingly, we expected the interaction of **1.1** and **3** but were nonetheless surprised by the results.

The insertion of propanediurea (PD) **3** (see Supporting Information for synthesis and characterization of **3**) into capsule **1.1** was revealed by the use of commercially available *n*-alkanes as guest probes: Figure 2 shows the ^1^H NMR spectra of all capsular assemblies obtained. The spectra were recorded at 280 K, since all the assemblies exhibited sharp NMR peaks at that temperature. The shortest alkane leading to an extension of the original capsule **1.1** was *n*-tetradecane (*n*-C_14_H_30_), which unexpectedly gave two new assemblies (labeled **I** and **II**; Figure 2 line 1). Both were formed by insertion of four molecules of **3** between the two halves of capsule **1.1** as indicated by integration of the ^1^H NMR spectra (see Supporting Information, Figure SI4). The spectrum features the anticipated upfield shifts of the guest signals, and the identical signals for C1/C14, C2/C13, C3/C12 and C4/C11 indicate some symmetry: the two ends of the capsules have the same magnetic environment. The spacing of the alkane guest's methylene signals indicates an extended conformation with little or no compression (coiling). Both assemblies appear achiral on the NMR timescale at 300 K and 280 K. However, as the sample is cooled to 240 K (Figure SI2), diastereotopic geminal guest protons are observed for the guest in the major assembly (**I**), indicating a chiral extended capsule structure (this diastereotopic splitting can be observed more clearly in assembly **I** encapsulating *n*-C_15_H_32_: see Figure SI2a). A *D*_4_-symmetric structure **1.3_4_.1** (Figure 3 a) is proposed for this assembly. This structure is merely the propanediurea counterpart to the chiral assembly **1.2_4_.1** generated by the glycoluril **2**. The latter capsule showed racemization when longer (C_17_–C_19_) *n*-alkane guests were inside, but those assemblies had to be heated to racemize. The faster racemization process for **1.3_4_.1** speaks for a weaker H-bonding pattern compared to **1.2_4_.1**, perhaps as a result of the decreased angle on the concave side of **3**.

**Figure 2:**
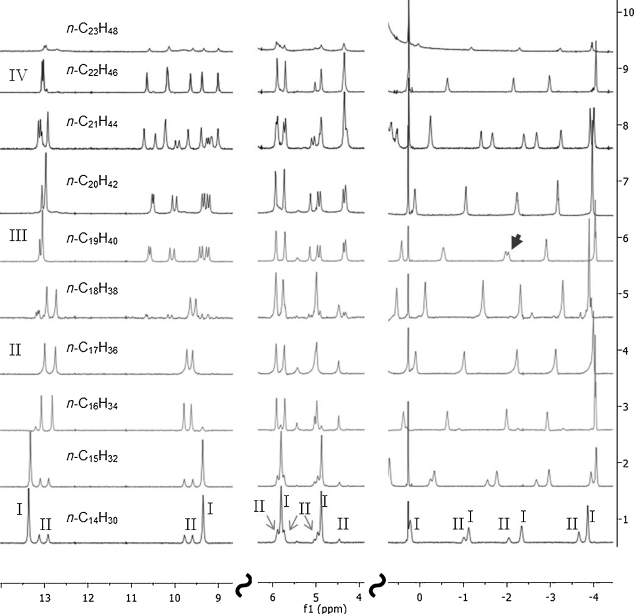
Overview of the 1H NMR spectra of all assemblies formed with guests ranging from *n*-tetradecane to *n*-tricosane. The concentrations are 4.8 mm in cavitand **1**, 10.4 mm in propanediurea **3** (20.8 mm in **3** for guests *n*-C_19_H_40_ to *n*-C_23_H_48_) and 24 mm in guest (148 mm for guests *n*-C_21_H_44_to *n*-C_23_H_48_) with [D_12_]mesitylene as solvent. The spectra were recorded at 280 K. The arrow indicates a diastereotopic CH_2_ group of the guest in the chiral assembly **III**.

**Figure 3:**
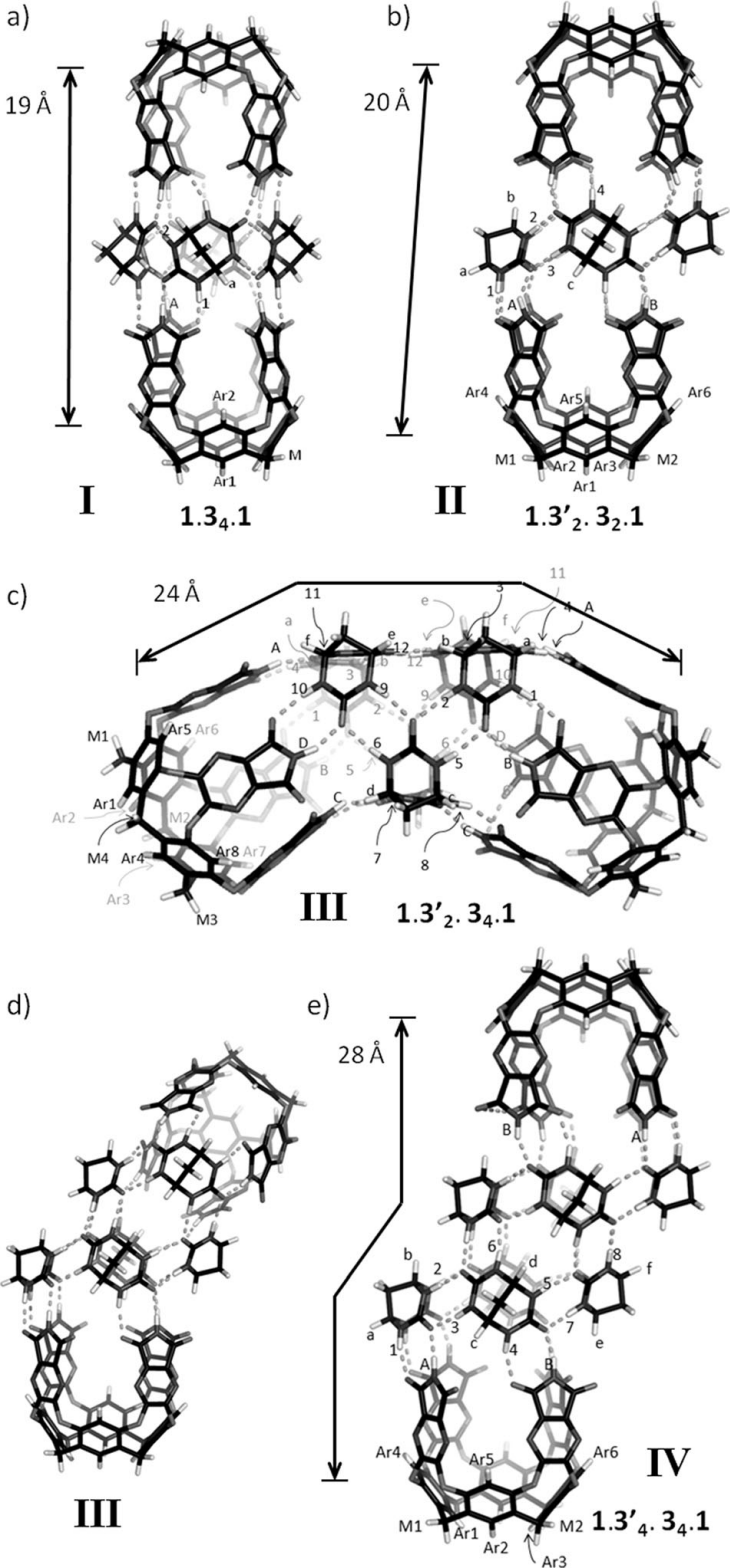
Overview of the formed assemblies **I** to **IV** and their approximate accessible cavity length. (Peripheral alkyl and aryl groups have been deleted for easier viewing.)

In the presence of *n*-C_14_H_30_, the minor assembly **II** can only be observed at lower temperatures (≤280 K). The characteristic signals of the capsule host are doubled but the encapsulated guest protons are not affected this way. In addition, the guest in **II** appears to be in a longer assembly as the methylene signals are shifted slightly downfield (ca. 0.1–0.3 ppm, Figure 2) when compared to their counterparts in the chiral assembly **I** (**1.3_4_.1**). As the sample is heated, the two assemblies interconvert with coalescence at ca. 300 K (Figure SI2). Similar behavior was observed on encapsulation of *n*-pentadecane. A ROESY experiment with encapsulated *n*-C_15_H_32_, recorded at 280 K, revealed that the signals for the imide N-H’s (located between *δ*=12.9 and 13.3 ppm) as well as the corresponding guest signals exchange, as the two different assemblies interconvert on the NMR-timescale (Figure SI5).

With longer *n*-alkanes the less symmetric assembly **II** becomes dominant—only a small amount of capsule **I** is formed when *n*-hexadecane is the guest and with *n*-heptadecane capsule **II** is formed exclusively. A DOSY experiment confirmed that all signals are part of the same assembly, using *n*-C_17_H_36_ as the guest (Figure SI6). This guest's methylene protons in **II** are not diastereotopic (even at low temperatures) indicating an achiral structure for this extended capsule. Molecular modeling led to a *C*_2*h*_-symmetric assembly **1.3′_2_.3_2_.1** (where the prime ′ denotes the different (horizontal) orientation of the propanediurea carbonyls as shown in Figure 3 b). The *C*_2*h*_ symmetry is responsible for the appearance of two imide N-H signals (A and B), four different ureido N-H-resonances (1–4), three PD-bridgehead C-H signals (a–c), two different methine C-H’s (M1 and M2) and six aromatic C-H signals (Ar1–6). In addition, a majority of signals (Figure 3 b: 1, 2, 3, 4, c, A, B, M1, M2, Ar1 and Ar5) are enantiotopic due to the plane of symmetry in the assembly.

There is a dynamic motion in the spacer belt of **1.3′_2_.3_2_.1**: The split signals of the capsule's components exchange even at lower temperature (280 K) as indicated by the appropriate exchange peaks observed in the ROESY spectrum (see Supporting Figure SI11). The relatively fast dynamics in the PD-belt (exchange on the NMR time scale) are responsible for the lack of information from the NOE-interactions (see Supporting Figure SI8): For instance, all PD-bridgehead C-H’s a, b, c display NOE-signals to all PD-N-H protons.

Further evidence for the structure of capsule **II** (**1.3′_2_.3_2_.1**) was provided by encapsulation of a chiral guest. Earlier work by Waldvogel et al.,[15] showed that 2-tetradecanol in capsule **1.1** induced local stereoselective helical folding, and we expected doubling of the enantiotopic host protons of **II** when a chiral magnetic environment was induced in **1.3′_2_.3_2_.1** by a suitable guest. This was provided by 2-heptadecanol, which upon encapsulation displayed diastereotopic signals of the guest at C_3_, C_4_ and C_5_ and the expected—now—diastereotopic hydrogens at the imide N-H signals of the host (Figure SI12). Separation of the other enantiotopic proton signals of the host is not clearly observable, due to signal overlap.

What governs the structural changes in the two assemblies? Longer guests apply pressure on the two ends of the capsule and favor the propanediurea orientations that give the capsule longer dimensions (the accessible cavity length in **II** is ca. 1 Å longer than in **I**).

Encapsulation of the longer *n*-octadecane (*n*-C_18_H_38_) resulted in the formation of yet a new assembly (**III**, Figure 2 line 5) in addition to **II** (ratio ca. 1:3.6). It features a more relaxed guest,[16] separated imide NH and varied PD signals. Furthermore, the new assembly does not coalesce with the major assembly (**1.3′_2_.3_2_.1**) even at 340 K (Figure SI14). The structural details of **III** were obtained with *n*-nonadecane since its formation is exclusive with this guest. First, a chiral structure is required since a diastereotopic CH_2_ group of the guest can be observed at 240–300 K (see arrow in Figure 2, and Figure SI16). Second, integration of the relevant signals revealed that the capsule contains six units of **3** (Figure SI15), an unprecedented number of spacer units. Molecular modeling led to the unusual “banana”-shaped structure **1.3′_2_.3_4_.1** (Figure 3 c) of *C*_2_ symmetry, which is formed by the incorporation of two additional units of **3** into assembly **II** (cf. Figure 3 b and d). The structure was supported by the NOE signals observed (Figure SI17), and a simulated NMR-spectrum at the DFT-level of theory (B3LYP/6-31G*) showed good agreement with the experimental spectrum (Figure SI21). The reduced symmetry is responsible for the appearance of four imide N-H’s (A–D, Figure 3 c), twelve different urea NH resonances (1–12), six PD-bridgehead C-H signals (a–f), four different methine C-H signals (M1–M4), and eight aromatic C-H signals (Ar1–8).

The unusual shape of **III** suggested its potential for shape-selective encapsulation of rigid guests and this proved to be the case. While the rigid and rectilinear *p*-pentaphenyl (**4**) was taken up by the known[12] doubly extended capsule **1.2_8_.1**, (Figure 4) it was not encapsulated by **III**, apparently due to the insurmountable shape incompatibilities (for spectra see Figure SI22). The reverse outcome was observed with bent dialkinylketone guest **5** (see Supporting Information for synthesis and characterization of **5**): it was encapsulated in the congruent host **III** but not in the linear capsule **1.2_8_.1** (for spectra see Figure SI23).

**Figure 4:**
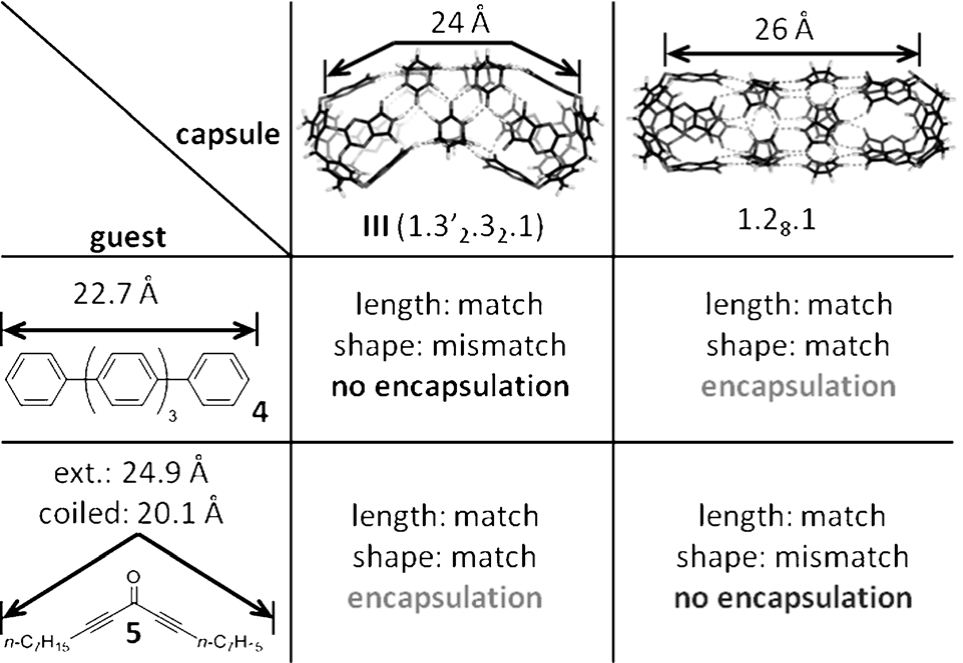
Selective encapsulation of complementary shaped guests in assembly **III** and the known double-extended cylindrical capsule **1.2_8_.1.**

With *n*-eicosane (*n*-C_20_H_42_) as guest, the same assembly **III** (**1.3′_2_.3_4_.1**) was observed and, as expected, the guest signals are shifted upfield (Figure 2; up to *δ*=0.5 ppm for protons at C4/C17) indicating a more compressed guest conformation. The use of *n*-heneicosane (*n*-C_21_H_44_) as guest gives rise to yet a new assembly (**IV**, Figure 2 line 8) in addition to **III** in a 1.1:1 ratio. As expected for a longer capsule, the guest is in a more extended conformation in **IV**. It was studied in more detail with *n*-docosane as guest since, once again, the formation of a single complex simplified the NMR spectra. Integration of the spectrum (Figure SI24) revealed the presence of eight PD units in the new, elongated assembly formulated as **IV**. The guest methylene protons are not diastereotopic even at low temperatures indicating an achiral structure for this double-extended capsule. We propose the *C*_2*h*_-symmetric structure for assembly **IV** (**1.3′_4_.3_4_.1**) shown in Figure 3 e, which is supported by ^1^H NMR data and NOE signals observed (Figure SI26). Due to the *C*_2*h*_ symmetry the assembly displays two imide N-H signals (A, B), eight different urea NH resonances (1–8), six PD-bridgehead C-H signals (a–f), two different methane C-H (M1, M2), and six aromatic C-H signals (Ar1–6).

With the longer *n*-tricosane (*n*-C_23_H_48_), the limits of self-assembly for this system are reached. Although assembly **IV** is still formed, the signal-to-noise ratio is quite low even in the presence of 60 equivalents of guest (Figure 2 line 10).

The structure of host **IV** follows the same expansion principles as apply for assemblies **II** and **III**: each cavitand **1** binds to one unit of **3** in a “horizontal” fashion at a corner of two walls; the two remaining walls bind one unit of **3** each in a “tilted” fashion. Since three out of four of the host structures self-assemble in such a fashion, it appears that this specific arrangement of the propanediurea units is thermodynamically favorable. The exception to this motif is assembly **I**, formed in the presence of the shortest guests (*n*-C_14_H_30_ and *n*-C_15_H_32_). In this case the packing coefficient (PC, Table 1) might explain the deviation from the thermodynamically most stable arrangement: Since host **I** is smaller than assembly **II**, *n*-C_14_H_30_ and *n*-C_15_H_32_ enjoy a more favorable PC inside. As can be seen in Table 1, the complexes prefer a PC of 53 to 54 %, which can be reached in host **II** starting with *n*-C_16_H_34_. By further increasing the guest length, the space inside **II** becomes more and more crowded, again triggering an extension of the capsule to host **III** with *n*-C_19_H_40_. A similar trend can be observed for guest *n*-C_21_H_44_.

**Table 1:** Packing coefficients (PC) for encapsulated alkane guests. The PC values highlighted in bold represent the major assemblies observed

Guest	Length[a]		PC[b]	PC[b]	PC[b]	PC[b]
	Extended	Coiled	[%] in **I**	[%] in **II**	[%] in **III**	[%] in **IV**
*n*-C_14_H_30_	19.2	14.4	**50**	47		
*n*-C_15_H_32_	20.5	15.3	**53**	50		
*n*-C_16_H_34_	21.7	16.2	55	**53**		
*n*-C_17_H_36_	23.0	17.2		**53**		
*n*-C_18_H_38_	24.3	18.1		**54**	52	
*n*-C_19_H_40_	25.5	19.1			**53**	
*n*-C_20_H_42_	26.8	20.1			**53**	
*n*-C_21_H_44_	28.0	21.0			55	**54**
*n*-C_22_H_46_	29.3	21.9				**55**
*n*-C_23_H_48_	30.6	22.7				**56**

[a] For determination of guest length see the Supporting Information.

[b] Assemblies were energy-minimized with guest inside at molecular mechanics level before determination of the cavity volume (for details see the Supporting Information).

The propensity of PD **3**, to form longer assemblies whenever the guest increases by only two or three carbon atoms is unusual: For glycolurils a change in host structure was observed when the guest length increased by five additional carbon atoms.[12] This high adaptability is caused by the extensive induced-fit behavior of both the host and the guest. It leads to complexes which stay very close to the ideal PC of slightly more than 50 % (Table 1) over a wide range of guest length.

In summary, the extension of the cylindrical capsule **1.1** with propanediurea units **3** results in the self-assembly of three new molecular capsules including the “banana”- and “S”-shaped structures **III** and **IV**. To best of our knowledge, these structures are the first examples of “bent” molecular capsules. The unique “banana” shape of host **III** allows the encapsulation of a complementary shaped guest that is not encapsulated in the known cylindrical capsules. These bent assemblies augur well for a richer space-shape and concomitant recognition properties for encapsulation complexes of the future.

## Supplementary material

Detailed facts of importance to specialist readers are published as ”Supporting Information”. Such documents are peer-reviewed, but not copy-edited or typeset. They are made available as submitted by the authors.
