# Supplementary material for: Self-Assembled Capsules of Unprecedented Shapes
Source: Angew Chem Int Ed Engl. 2011 Aug 26;50(50):12003–7. doi: 10.1002/anie.201102548 (PMC3272346; doi:10.1002/anie.201102548)
Supplement: Supplementary file 1 [file anie0050-12003-SD1.pdf]

Angewandte Chemie
Eine Zeitschrift der Gesellschaft Deutscher Chemiker

Supporting Information
© Wiley-VCH 2011
69451 Weinheim, Germany

Self-Assembled Capsules of Unprecedented Shapes**

*Konrad Tiefenbacher, Dariush Ajami, and Julius Rebek Jr.**

anie_201102548_sm_miscellaneous_information.pdf

Table of Contents

Materials and General Methods .... S 2

Synthesis of propanediurea 3 .... S 2

Synthesis of nonadeca-8,11-diyn-10-one (5) .... S 4

General procedure for the encapsulation studies: .... S 6

Determination of guest length .... S 6

Determination of packing coefficients (PC) .... S 7

1H-NMR spectra of encapsulated *n*-tetradecane in host I/II .... S 9

1H-NMR spectra of encapsulated *n*-pentadecane in host I/II .... S 10

ROESY spectrum of encapsulated *n*-pentadecane in host I/II at 280 K. .... S 13

NMR spectra of encapsulated *n*-heptadecane in host II .... S 14

Encapsulation of 2-heptadecanol in host II .... S 17

NMR spectra of encapsulated *n*-octadecane in host II/III .... S 20

NMR spectra of encapsulated *n*-nonadecane in host III .... S 21

Shape selective encapsulation studies .... S 27

NMR spectra of encapsulated *n*-docosane (*n*-$C_{22}H_{46}$) in host IV .... S 29

References .... S 32

raw data (B3LYP/6-31G*) for the calculated NMR spectrum .... S 33

Materials and General Methods

All reactions were carried out under an atmosphere of argon unless otherwise indicated. Analytical thin-layer chromatography (TLC) was performed on Silicycle 60 F254 glass-baked plates. ^{1}H NMR and ^{13}C NMR spectra were recorded at 600 MHz and 150 MHz respectively, using a Bruker DRX-600 spectrometer equipped with a 5 mm QNP probe. Chemical shifts of ^{1}H NMR and ^{13}C NMR are given in ppm by using $CHCl_3$ or DMSO as references (7.26 ppm, 2.50 ppm respectively for ^{1}H spectrum, and 77.16 ppm, 39.52 ppm respectively for ^{13}C spectrum). Coupling constants (J) are reported in Hertz (Hz). Standard abbreviations indicating multiplicity were used as follows: s (singlet), br (broad), d (doublet), t (triplet), q (quartet), m (multiplet). MALDI-TOF spectra and high-resolution mass spectra (HRMS) were recorded on an Applied Biosystems Voyager STR (2) apparatus and an Agilent ESI-TOF mass spectrometer respectively. Anhydrous CH_2Cl_2, NEt_3 and Et_2O were taken from a solvent drying system (SG Water USA).

All deuterated solvents were purchased from Cambridge Isotope Laboratories, Inc. All *n*-alkanes, *n*-butyl lithium (1.6 M in hexane), urea and ethyl formate were purchased from Sigma-Aldrich. 2,2-di-*n*-octyl-1,3-propanediol and *p*-pentaphenyl were purchased from TCI America. Oxalyl chloride was purchased from Fluka. 1-nonyne was purchased from Alfa Aesar. Pyridinium chlorochromate and DMSO were purchased from Acros. Acetic acid, sulfuric acid, celite and molecular sieves 4Å powder were purchased from Fisher Chemicals. All chemicals were used as received.

Cavitand **1** was prepared according to literature procedures.[1]

Synthesis of propanediurea 3

The synthesis of propanediurea[2,3] **3** is shown in Scheme SI 1: Swern oxidation of the commercially available diol **6** delivered dialdehyde **7**, which on treatment with urea under acidic conditions yielded **3** in 81% over two steps.

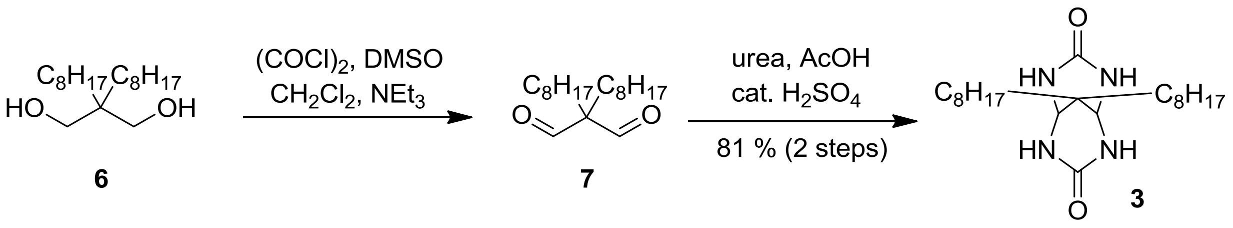

Scheme SI 1

2,2-di-n-octylmalonaldehyde (**7**)

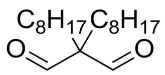

To a solution of oxalyl chloride (0.31 mL, 3.66 mmol) in CH_2Cl_2 (8.8 mL) was added DMSO (0.52 mL, 7.3 mmol) dissolved in CH_2Cl_2 (1 mL) dropwise at -78 °C. After stirring at this temperature for 40 min, a solution of commercially available 2,2-di-n-octyl-1,3-propanediol (**6**) (500 mg, 1.66 mmol) in CH_2Cl_2 (2 mL) was introduced to the suspension. The mixture was kept at -78 °C for 90 min. NEt_3 (1.6 mL, 11.6 mmol) was added and after stirring for additional 30 min at -78 °C, the reaction was warmed to 20 °C. It was quenched by the addition of saturated aqueous NH_4Cl after 60 min at 20 °C. The aqueous phase was extracted with CH_2Cl_2 (3x), the combined organic phases were washed with 1 N HCl (1x), dried over sodium sulfate, filtered and the solvent removed under vacuum to yield crude dialdehyde **7** (500 mg), which was used without further purification.

^{1}H NMR (600 MHz, $CDCl_3$, 300K) δ=9.73 (s, 2H), 1.81-1.76 (m, 4H), 1.31-1.19 (m, 24H), 0.87 (t, J = 6.8 Hz, 6H); **^{13}C NMR** (150 MHz, $CDCl_3$, 300K) δ=202.3 (CH, 2C), 65.3 (C), 31.9 (CH_2, 2C), 31.1 (CH_2, 2C), 30.3 (CH_2, 2C), 29.3 (CH_2, 2C), 29.3 (CH_2, 2C), 24.1 (CH_2, 2C), 22.8 (CH_2, 2C), 14.2 (CH_3, 2C).

HRMS(EI) calcd. for $C_{19}H_{37}O_2$ $[M+H]^+$: 297.2788, found: 297.2784.

9,9-di-n-octyl-2,4,6,8-tetraazabicyclo[3.3.1]nonane-3,7-dione (**3**)

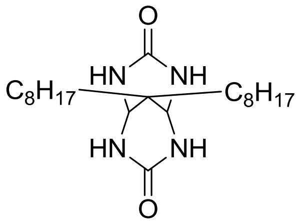

To a mixture of crude dialdehyde **7** (500 mg, th: 1.66 mmol) in acetic acid (1.0 mL) was added urea (219 mg, 3.65 mmol) and a drop of conc. sulfuric acid. After heating at 100 °C for 2h, the mixture was cooled to 20 °C and water (10 mL) was added. The solid was filtered through a glass frit, washed with water (2x) and hexane (2x) and dried under vacuum to give propanediurea **3** (510 mg, 81% over 2 steps) as a white crystalline solid.

^{1}H NMR (600 MHz, d6-DMSO, 343K) δ=6.62 (s, 4H), 3.95 (s, 2H), 1.45 (s, 4H), 1.28 (s, 24H), 0.88 (t, J = 7.0 Hz, 6H).

^{13}C NMR (150 MHz, d6-DMSO, 343K) δ=153.8 (C, 2C), 61.6 (CH, 2C), 34.7 (C), 30.8 (CH_2, 2C), 29.3 (CH_2, 2C), 29.2 (CH_2, 2C), 28.3 (CH_2, 2C), 28.1 (CH_2, 2C), 22.5 (CH_2, 2C), 21.5 (CH_2, 2C), 13.3 (CH_3, 2C).

HRMS(EI) calcd. for $C_{21}H_{41}N_4O_2$ $[M+H]^+$: 381.3224, found: 381.3222.

Mp > 210 °C

IR $\tilde{\nu}$= 3258 (br), 2920, 2851, 1641, 1525, 1461, 1215, 1125, 771 cm^{-1}

Synthesis of nonadeca-8,11-diyn-10-one (5)

The literature procedure for heneicosa-9,12-diyn-11-one[4] was adapted:

nonadeca-8,11-diyn-10-ol

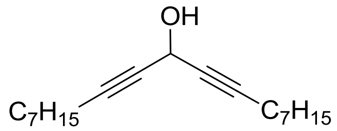

To a solution of 1-nonyne (0.80 mL, 4.88 mmol) in Et_2O (6.5 mL) was added *n*-BuLi (1.6 M in hexane, 3.0 mL, 4.86 mmol) at -78 °C. After stirring at that temperature for 30 min, ethyl formate (0.16 mL, 2.02 mmol) in Et_2O (1.0 mL) was added. The

solution was allowed to warm to 20 °C over 30 min and subsequently quenched by the addition of aq. NH_4Cl-solution (10 mL). The aqueous phase was extracted with Et_2O (2x), the combined organic phases were dried over sodium sulfate, filtered and the solvent removed under vacuum. Purification by column chromatography (60 mL silica gel) using hexane/ethyl acetate 18:1 → 15:1 as an eluent yielded nonadeca-8,11-diyn-10-ol (396 mg, 71%) as a colorless oil.

^{1}H NMR (600 MHz, $CDCl_3$, 300K) δ=5.09 (dt, *J* = 7.1, 2.0 Hz, 1H), 2.22 (td, *J* = 7.2, 2.0 Hz, 4H), 2.03 (d, *J* = 7.1 Hz, 1OH), 1.57 – 1.48 (m, 4H), 1.44 – 1.34 (m, 4H), 1.34 – 1.21 (m, 12H), 0.88 (t, *J* = 7.0 Hz, 6H).

^{13}C NMR (150 MHz, $CDCl_3$, 300K) δ=85.3 (C, 2C), 78.2 (C, 2C), 52.8 (CH), 31.9 (CH_2, 2C), 29.0 (CH_2, 2C), 28.9 (CH_2, 2C), 28.5 (CH_2, 2C), 22.8 (CH_2, 2C), 18.9 (CH_2, 2C), 14.2 (CH_3, 2C).

HRMS(EI) calcd. for $C_{19}H_{32}ONa$ $[M+Na]^+$: 299.2345, found: 299.2354.

IR $\tilde{\nu}$= 2925, 2855, 2204, 1627, 1461, 1326, 1237, 722 cm^{-1}

nonadeca-8,11-diyn-10-one (5)

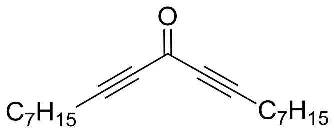

To a solution of nonadeca-8,11-diyn-10-ol (250 mg, 0.90 mmol) in CH_2Cl_2 (9.2 mL) was added celite (0.27 g), molecular sieves powder (4Å, 0.27 g) and pyridinium chlorochromate (291 mg, 1.35 mmol) at 20 °C. After stirring at that temperature for 2 h, the mixture was filtered through a plug of silica gel (15 mL), eluted with CH_2Cl_2 and concentrated under vacuum. Purification by column chromatography (50 mL silica gel) using hexane/ethyl acetate 15:1 as an eluent yielded nonadeca-8,11-diyn-10-one (**5**) (196 mg, 79%) as a colorless oil.

^{1}H NMR (600 MHz, $CDCl_3$, 300K) δ=2.38 (t, *J* = 7.2 Hz, 4H), 1.63 – 1.55 (m, 4H), 1.45 – 1.36 (m, 4H), 1.35 – 1.23 (m, 12H), 0.89 (t, *J* = 7.0 Hz, 6H).

^{13}C NMR (150 MHz, $CDCl_3$, 300K) δ=161.6 (C), 94.9 (C, 2C), 82.5 (C, 2C), 31.8 (CH_2, 2C), 29.0 (CH_2, 2C), 28.8 (CH_2, 2C), 27.7 (CH_2, 2C), 22.7 (CH_2, 2C), 19.3(CH_2, 2C), 14.2 (CH_3, 2C).

HRMS(EI) calcd. for $C_{19}H_{31}O$ $[M+H]^+$: 275.2369, found: 275.2374.

IR $\tilde{\nu}$= 2925, 2856, 2206, 1626, 1461, 1326, 1237, 722 cm^{-1}

General procedure for the encapsulation studies:

To a mixture of cavitand **1** (4.0 mg, 0.0024 mmol), propanediurea **3** (2.0 mg, 0.0052 mmol in case of C14-C18 *n*-alkane guest; 4.0 mg, 0.0104 mmol in case of C19-C23 *n*-alkane guest) and guest (0.012 mmol in case of C14-C20 *n*-alkane guest; 0.072 mmol in case of C21-C23 *n*-alkane guest) in a NMR-tube was added 1,3,5-trimethylbenzene-d12 (0.50 mL). The suspension was homogenized by repeated heating via heat gun and agitation. The spectra were recorded after letting the sample equilibrate for 24 h at 20 °C.

Determination of guest length

The length of the guest was estimated by measuring the distance between the hydrogen atoms at each end of the guest as indicated in Figure 1 (using Spartan 03, Wavefunction Inc.). The dihedral angles were fixed at 180° (extended *n*-alkanes) or 60° (coiled *n*-alkanes). To account for the size of the terminal hydrogen atoms, a value of 0.5 Å (approx. the radius of a hydrogen atom) was added to each end (Fig. SI 1).

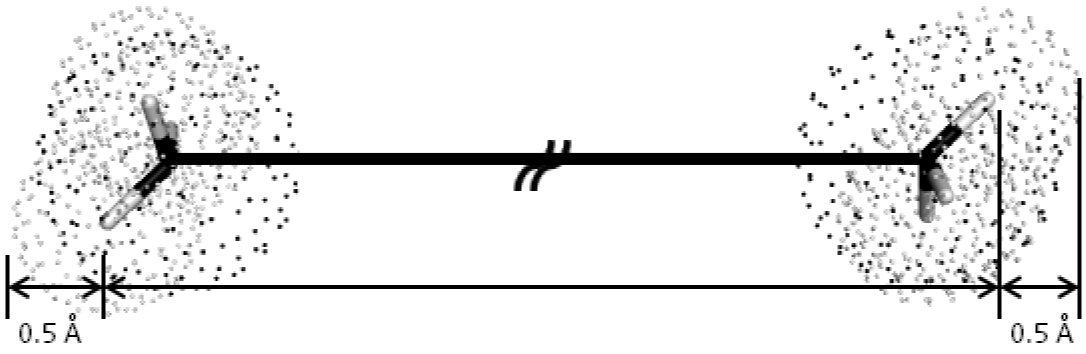

Figure SI 1: Determination of the guest length.

Determination of packing coefficients (PC)

The assemblies were first optimized with the appropriate guests inside at molecular mechanics level of theory (Spartan 03, Wavefunction Inc.). In case a guest was too long for the host in the extended conformation (dihedral angle of 180 °), coiling (dihedral angle of 60 °) was applied to the ends of the guest to fit the host dimensions. After optimization of the assemblies, the guest was removed and the file saved in the pdb-format. The determination of the cavity volume was performed using Swiss-PdbViewer 4.0.1 (Swiss Institute of Bioinformatics; http://www.expasy.org/spdbv/) at the highest quality level (Surface Preferences: Quality 6). The volume of the guests was determined using the same program. The fraction of V(guest) to V(cavity) equals the packing coefficient. All determined values are displayed in Table SI 1.

guest	volume of guest [Å^3]	Cavity volume in host [Å^3]				PC [%] in **I**	PC [%] in **II**	PC [%] in **III**	PC [%] in **IV**
		**I**	**II**	**III**	**IV**				
***n*-$C_{14}H_{30}$**	245	486	524			**50**	47		
***n*-$C_{15}H_{32}$**	261	494	524			**53**	50		
***n*-$C_{16}H_{34}$**	278	510	524			55	**53**		
***n*-$C_{17}H_{36}$**	295		552				**53**		
***n*-$C_{18}H_{38}$**	312		582	597			**54**	52	
***n*-$C_{19}H_{40}$**	328			614				**53**	
***n*-$C_{20}H_{42}$**	346			650				**53**	
***n*-$C_{21}H_{44}$**	362			653	665			55	**54**
***n*-$C_{22}H_{46}$**	379				685				**55**
***n*-$C_{23}H_{48}$**	396				713				**56**

Table SI 1: Measured guest and cavity volumes and the corresponding packing coefficients.

1H-NMR spectra of encapsulated *n*-tetradecane in host I/II

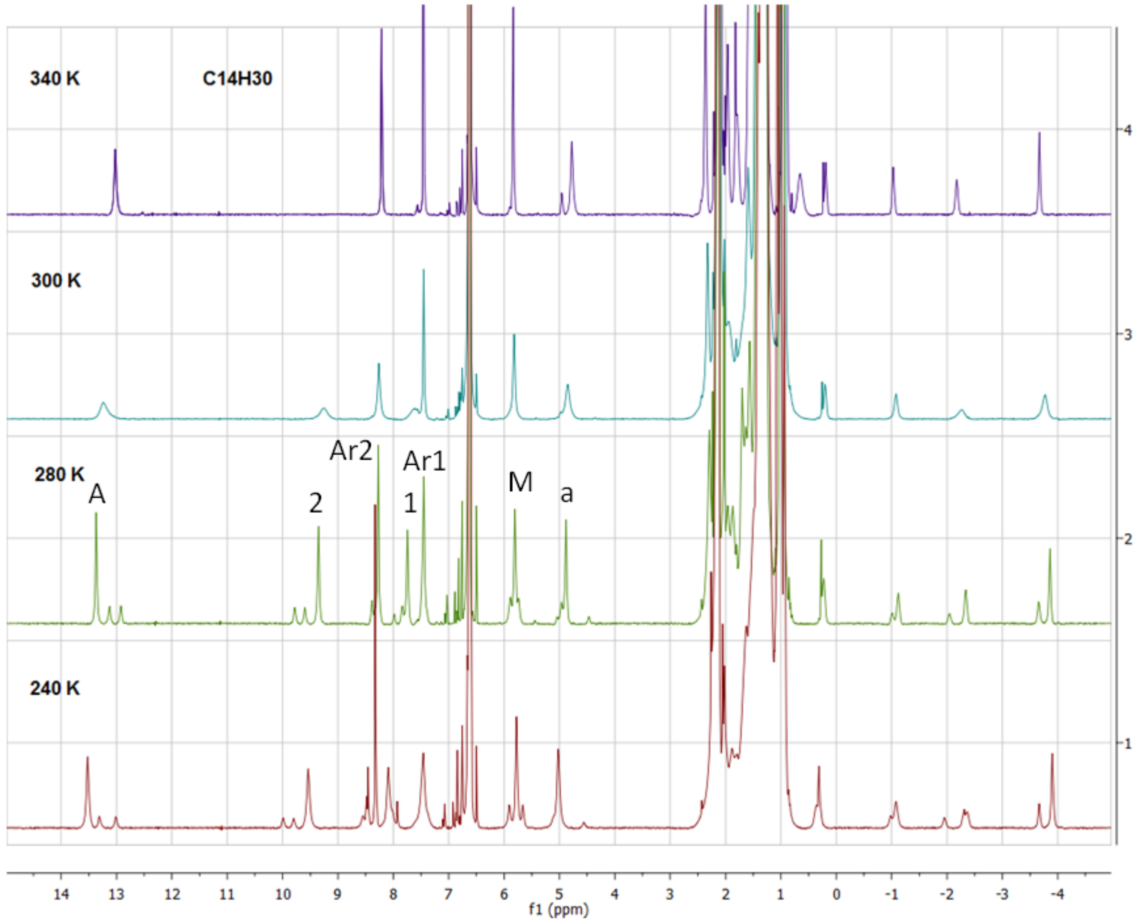

Fig. SI 2: 1H-NMR spectra of encapsulated *n*-tetradecane in host I/II at 240-340 K.

1H-NMR spectra of encapsulated *n*-pentadecane in host I/II

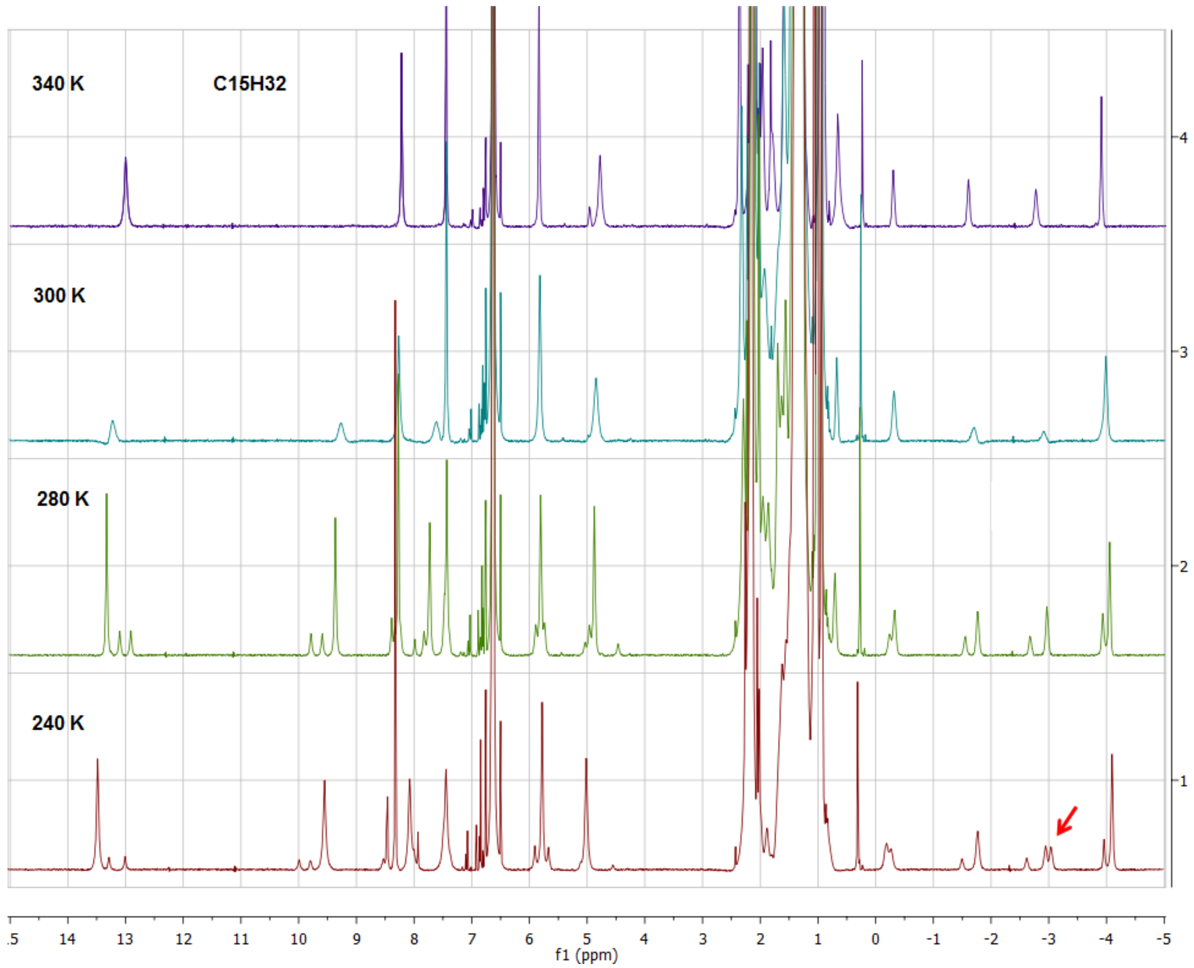

Fig. SI 2a: 1H-NMR spectra of encapsulated *n*-pentadecane in host I/II at 240-340 K.

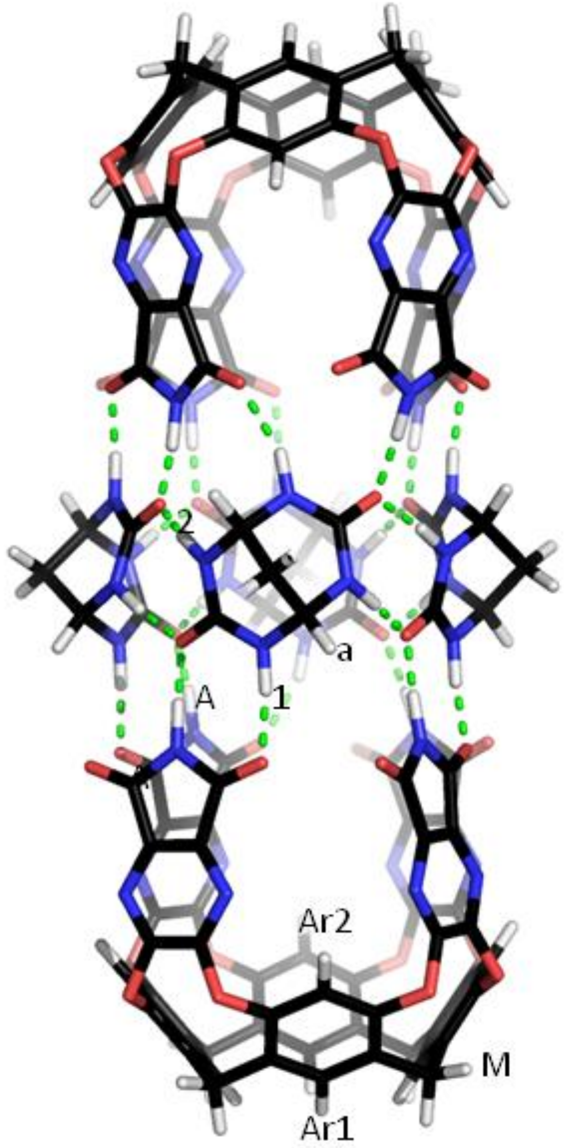

Fig. SI 3: Model of host **I** (**1**.$\mathbf{3}_4$.**1**) and assignment of proton-signals (cf. Fig. SI 2)

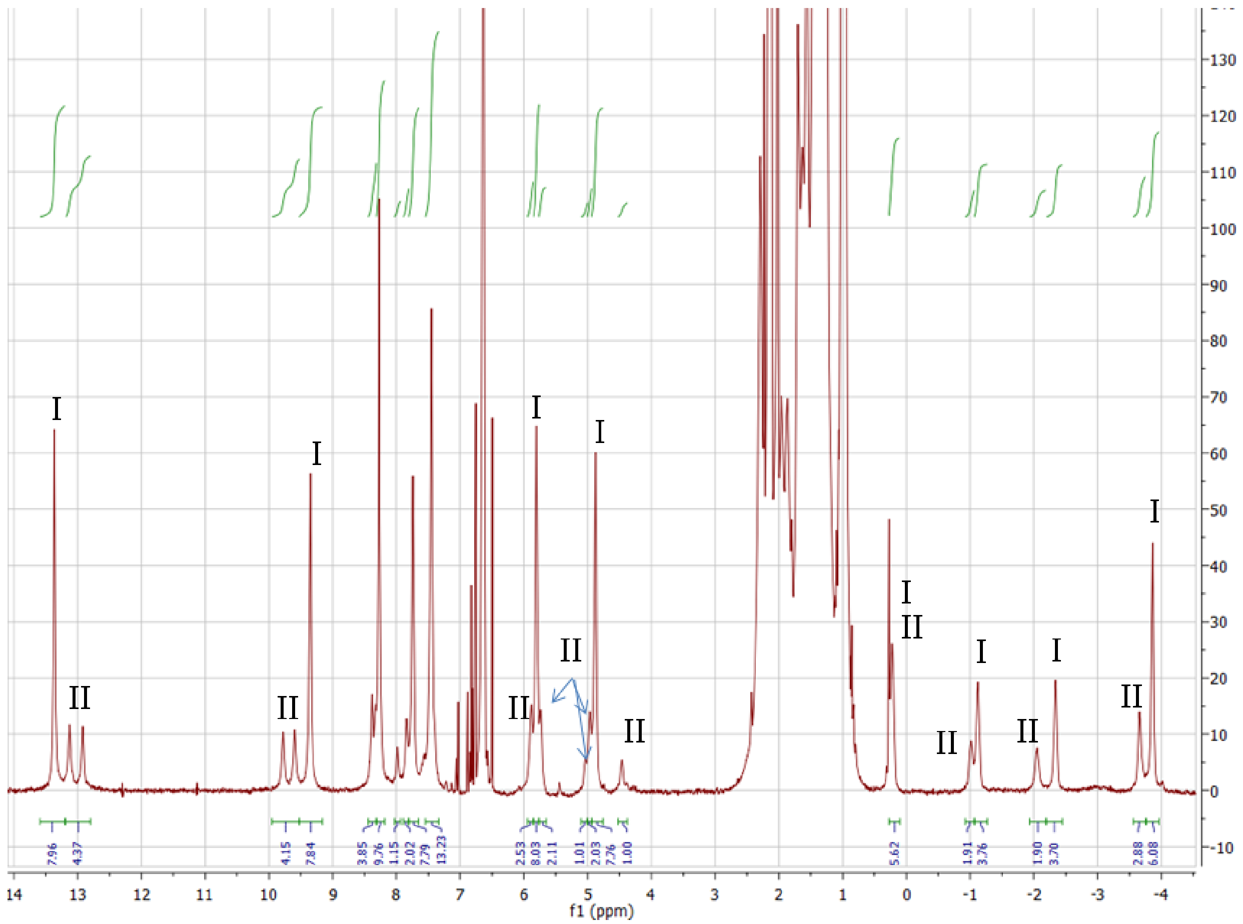

Fig. SI 4: Integrated 1H-NMR spectra of encapsulated n-tetradecane in host I/II at 280 K.

ROESY spectrum of encapsulated *n*-pentadecane in host I/II at 280 K.

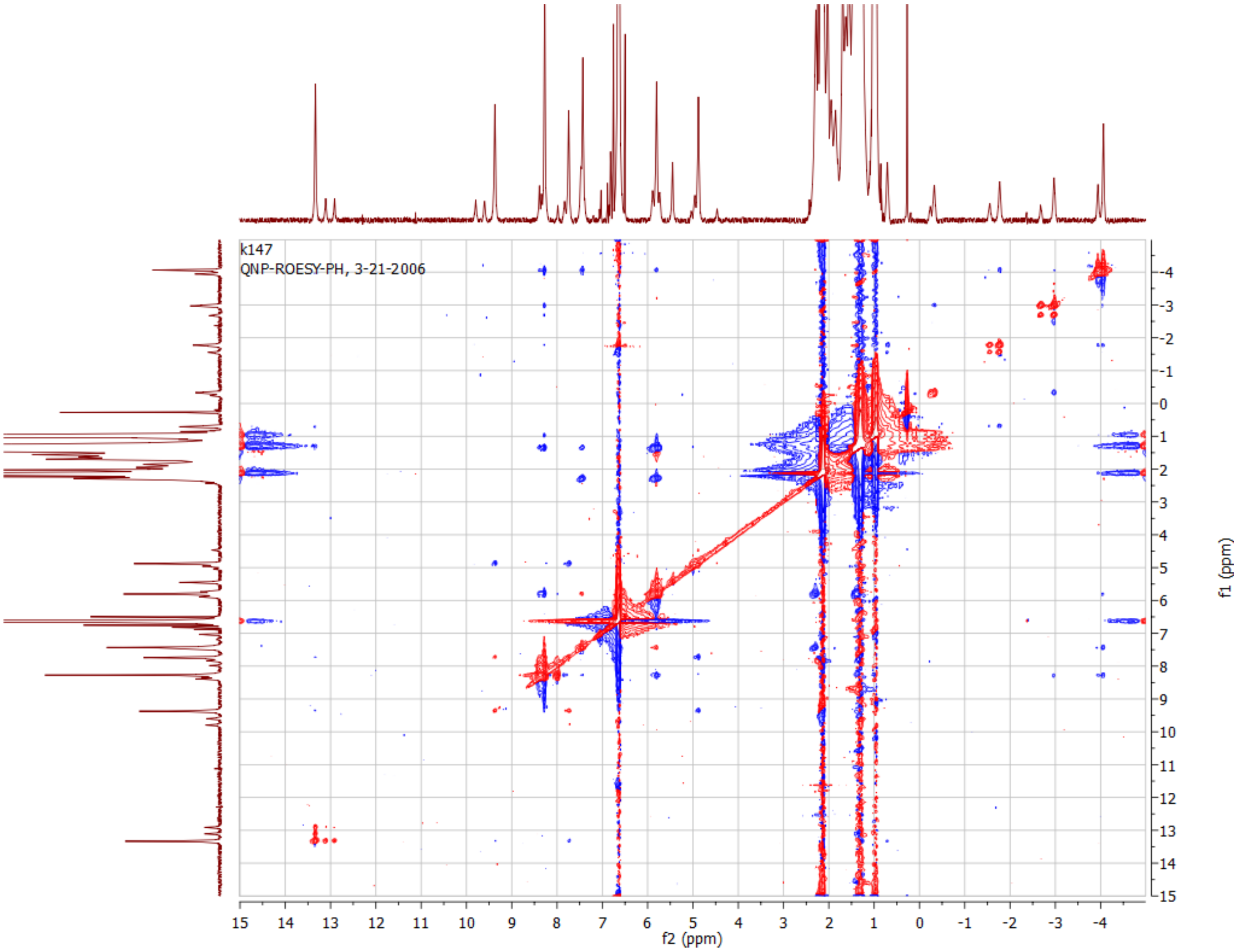

Fig. SI 5: ROESY spectrum of encapsulated *n*-pentadecane in host I/II at 280 K

NMR spectra of encapsulated *n*-heptadecane in host II

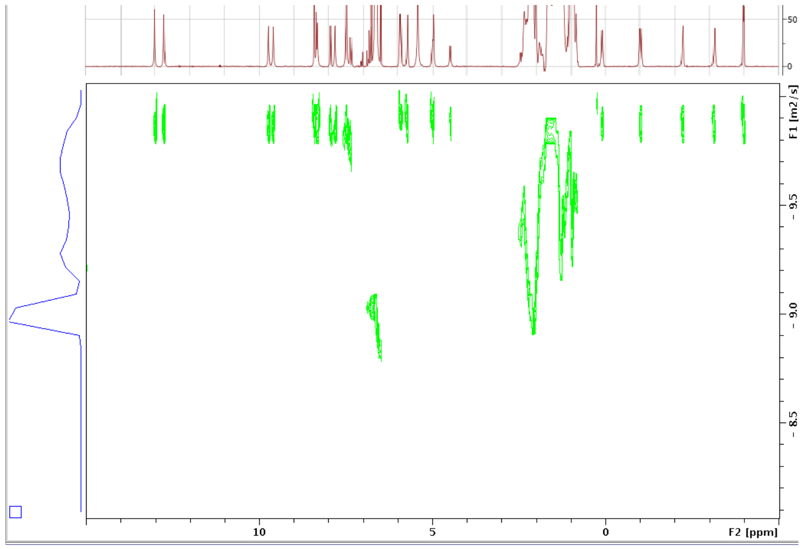

Fig. SI 6: DOSY spectrum of encapsulated *n*-heptadecane in host II.

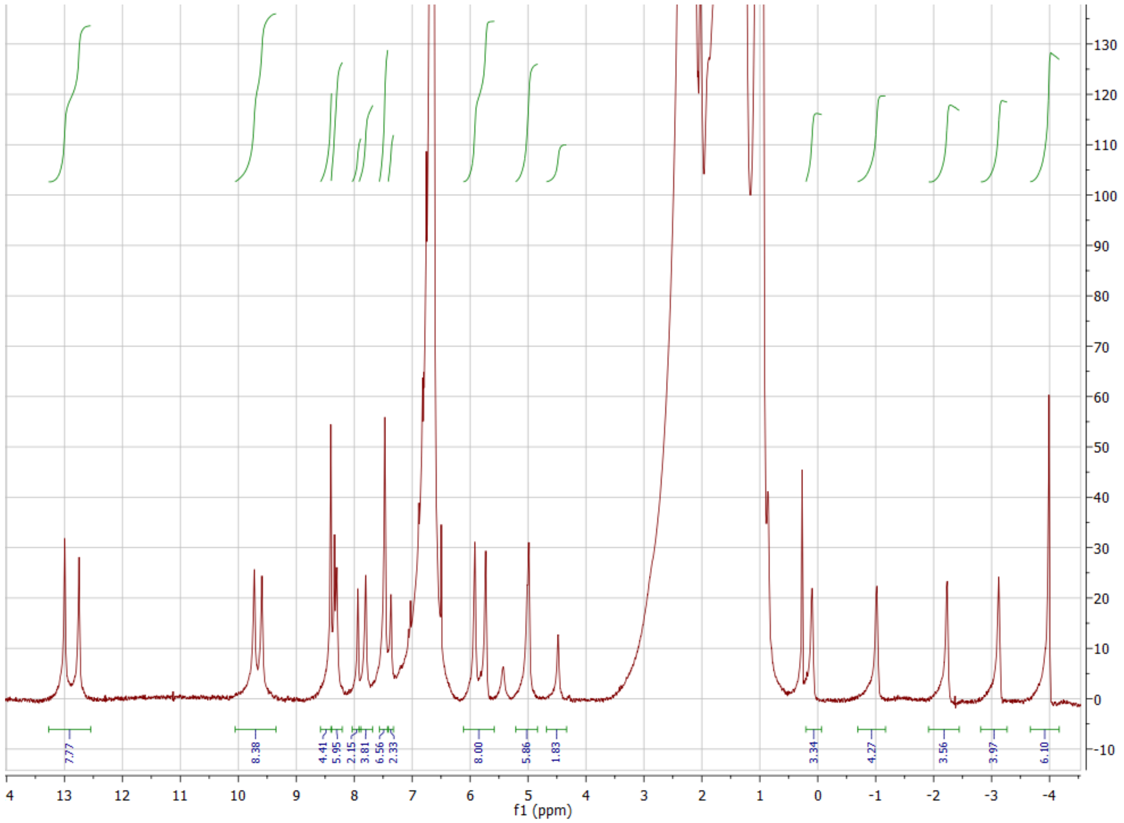

Fig SI 7: Integrated 1H-NMR spectra of encapsulated *n*-heptadecane in host **II** at 280 K.

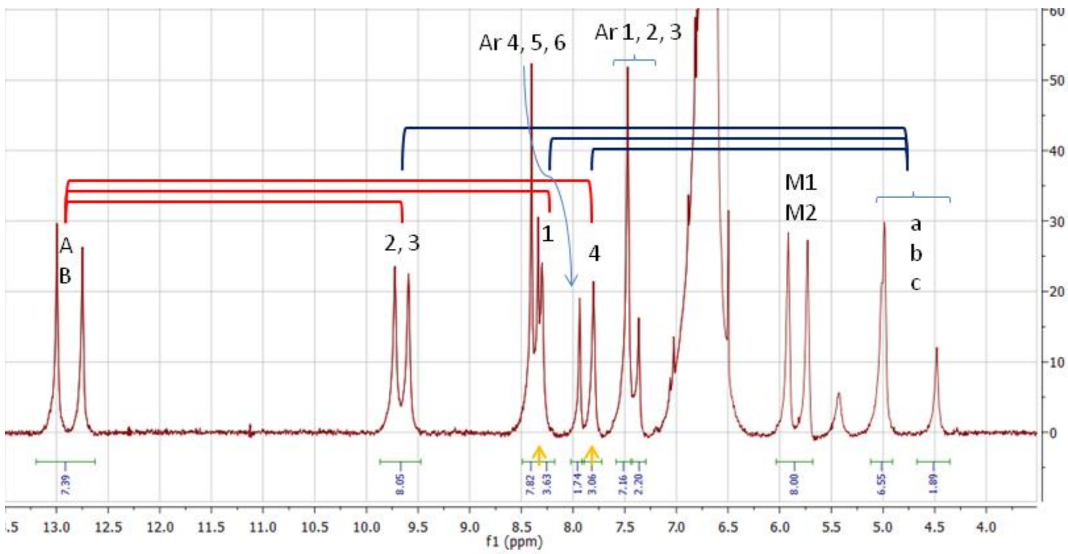

Fig. SI 8: Observed NOE-signals in assembly **II** (**1.3’$_2$.3$_2$.1**) with *n*-heptadecane as guest (cf. Fig. SI 11).

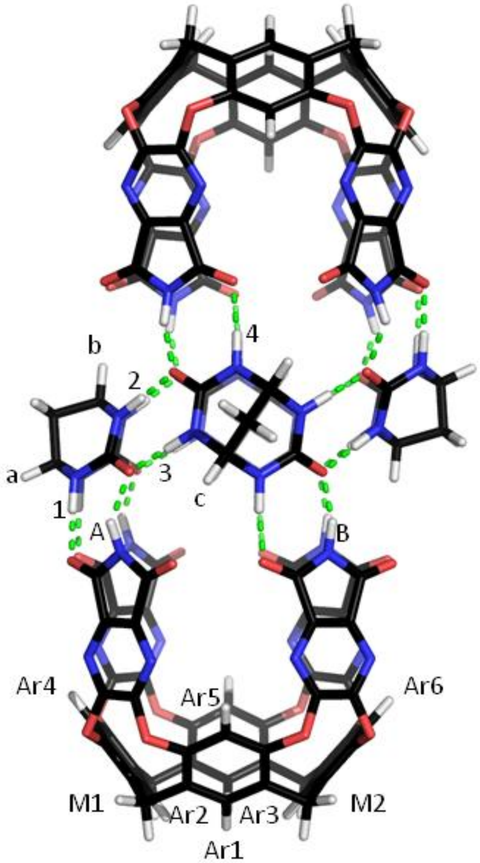

Fig. SI 9: Proposed structure of the achiral capsule II (**1.3’$_2$.3$_2$.1).** The C_{2h}-symmetry accounts for the doubling of the 1H-NMR signals: There are two magnetically different imide N-H signals (A and B), two ureido NH-signals binding to another PD-unit (2, 3), six non-equivalent aromatic signals (Ar1-6), two different ureido NH-signals binding to an imide-carbonyl (1, 4), two non-equivalent methine signals (M1 and M2) and three distinct PD-signals (a-c).

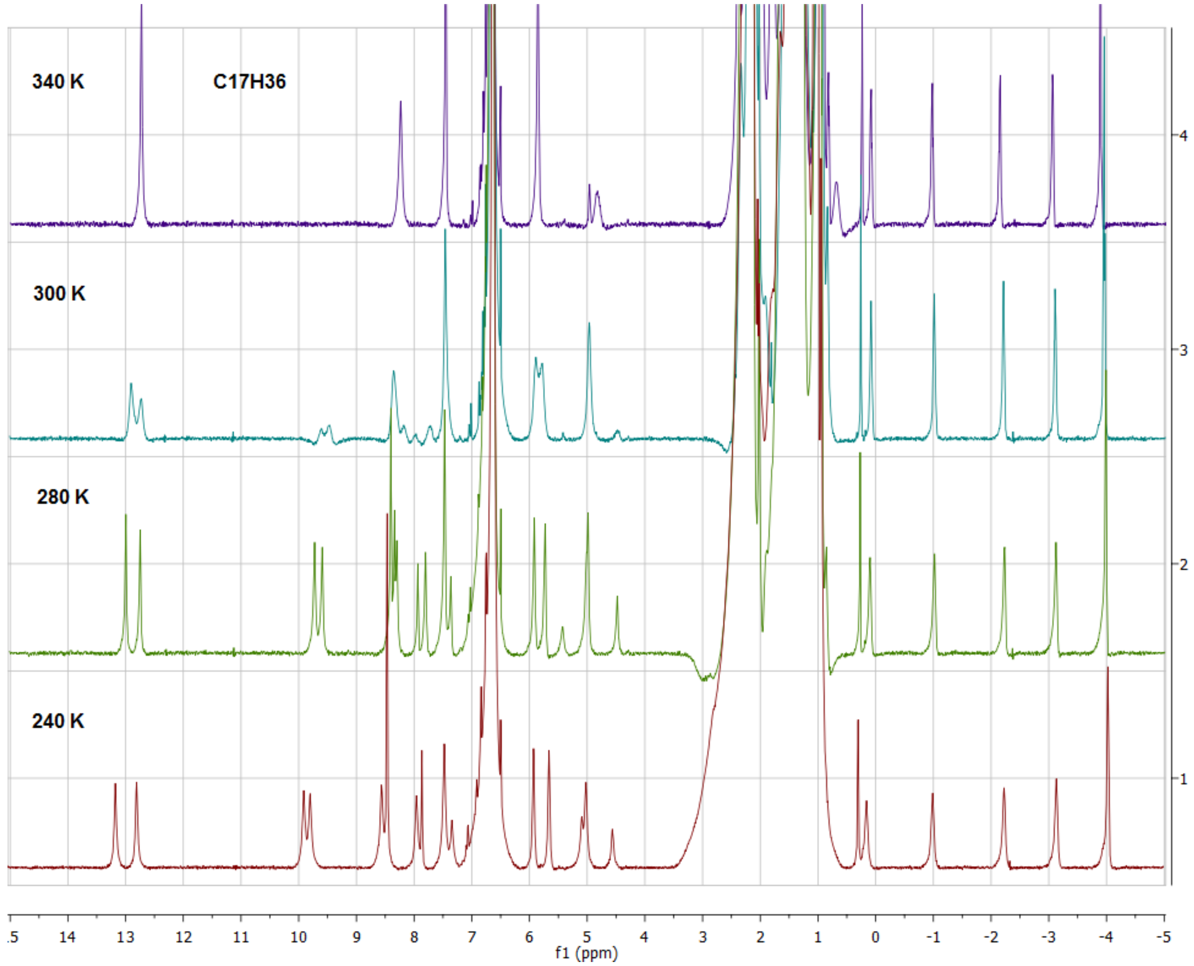

Fig. SI 10: 1H-NMR spectra of encapsulated *n*-heptadecane in host **II** at 240 – 340 K.

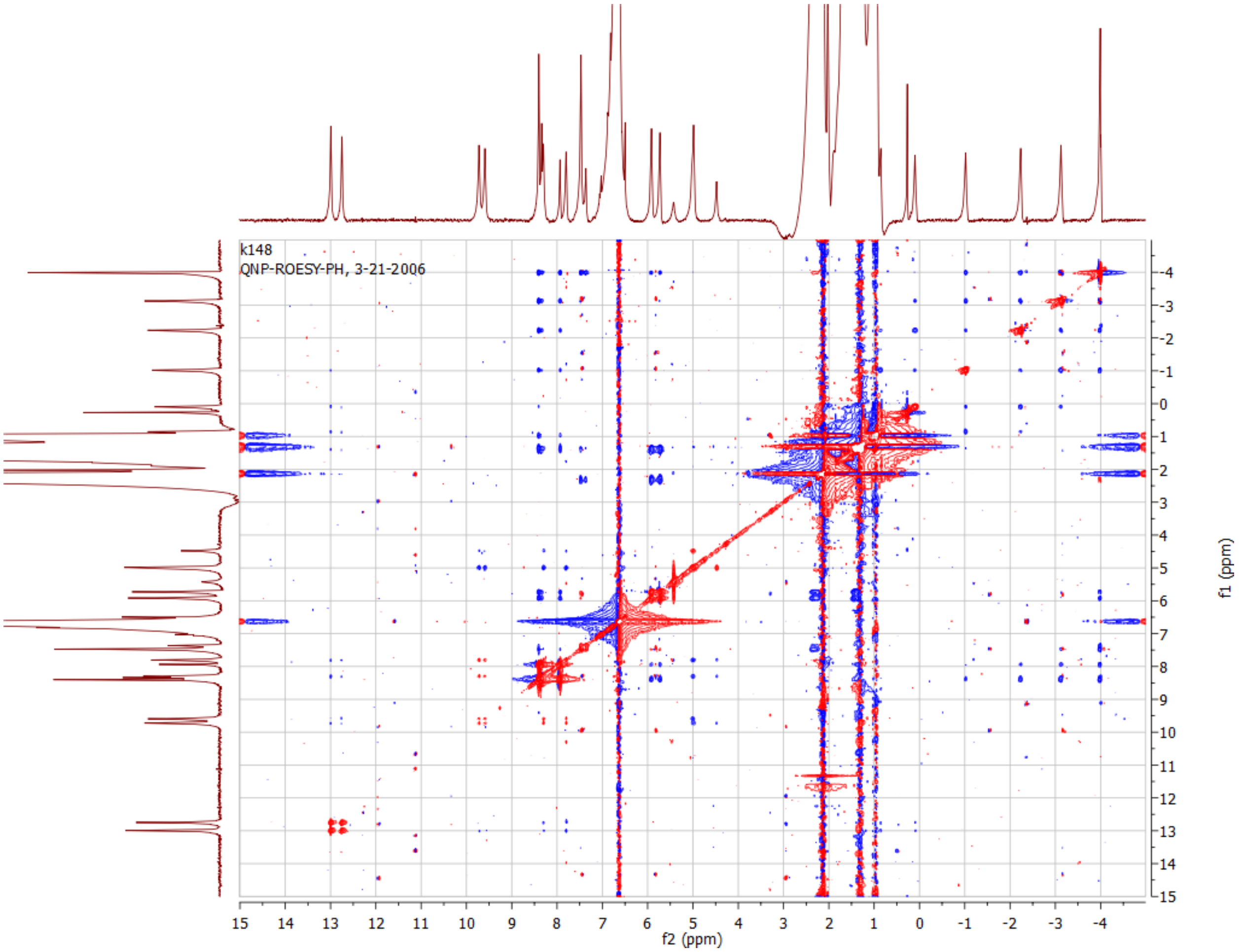

Fig. SI 11: ROESY spectrum of encapsulated *n*-heptadecane in host **II** at 280 K. The NOE-signals of this spectrum are displayed in Fig. SI 8.

Encapsulation of 2-heptadecanol in host II

Due to the non-symmetry of the guest 2-heptadecanol, four imide-signals should be detectable (as compared to two in the case of the symmetric n-heptadecane). The further splitting (at ca. 12.7 ppm, at 240K and 280K) is caused by the magnetic non-equivalence of the enantiotopic signals in the chiral field. Splitting of the other enantiotopic protons of the host is not clearly identifiable, due to signal overlap in the aromatic region.

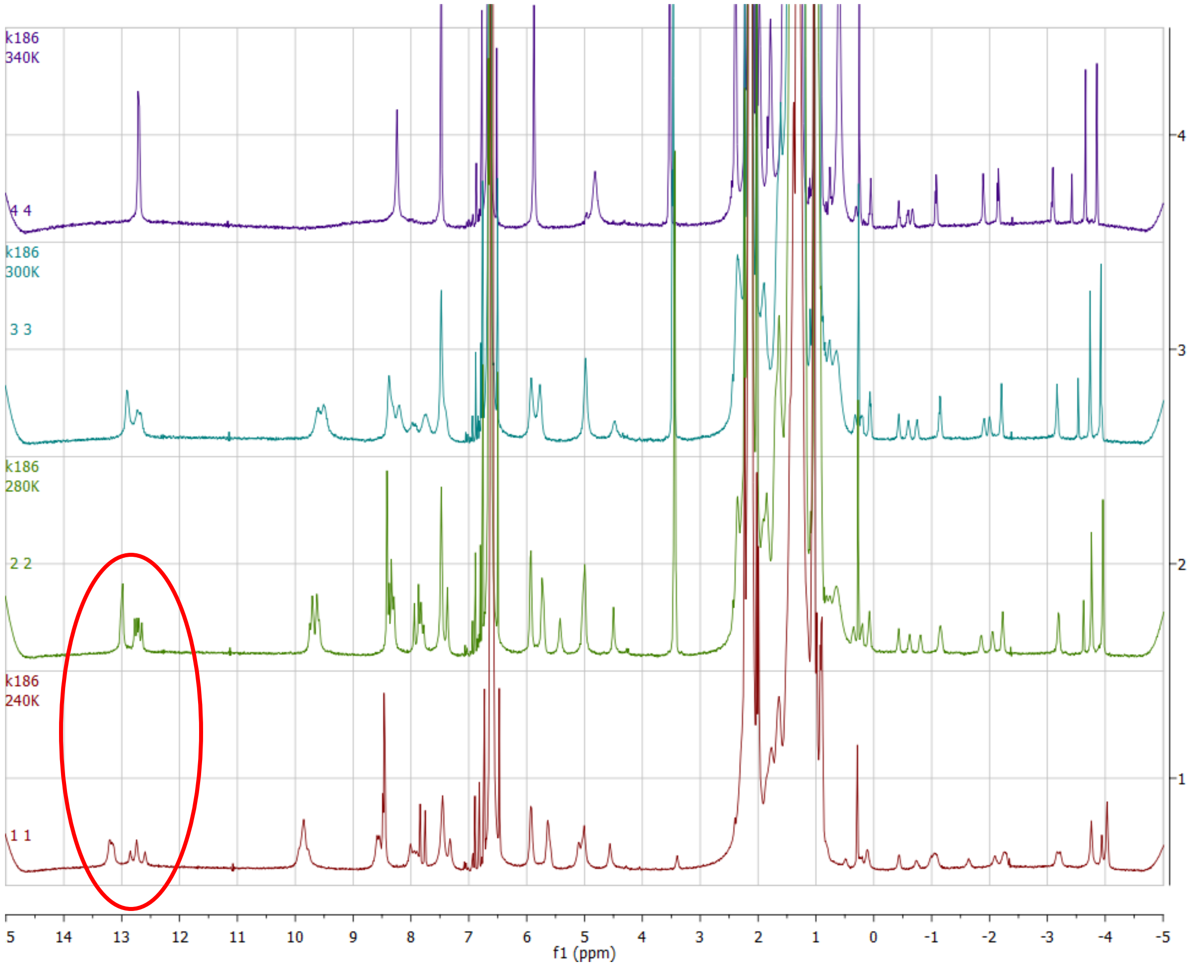

Fig. SI 12: Encapsulation of 2-heptadecanol results in a local chiral field and resolution of enantiotopic imid-signals in **1.3'$_2$.3$_2$.1** (**II**).

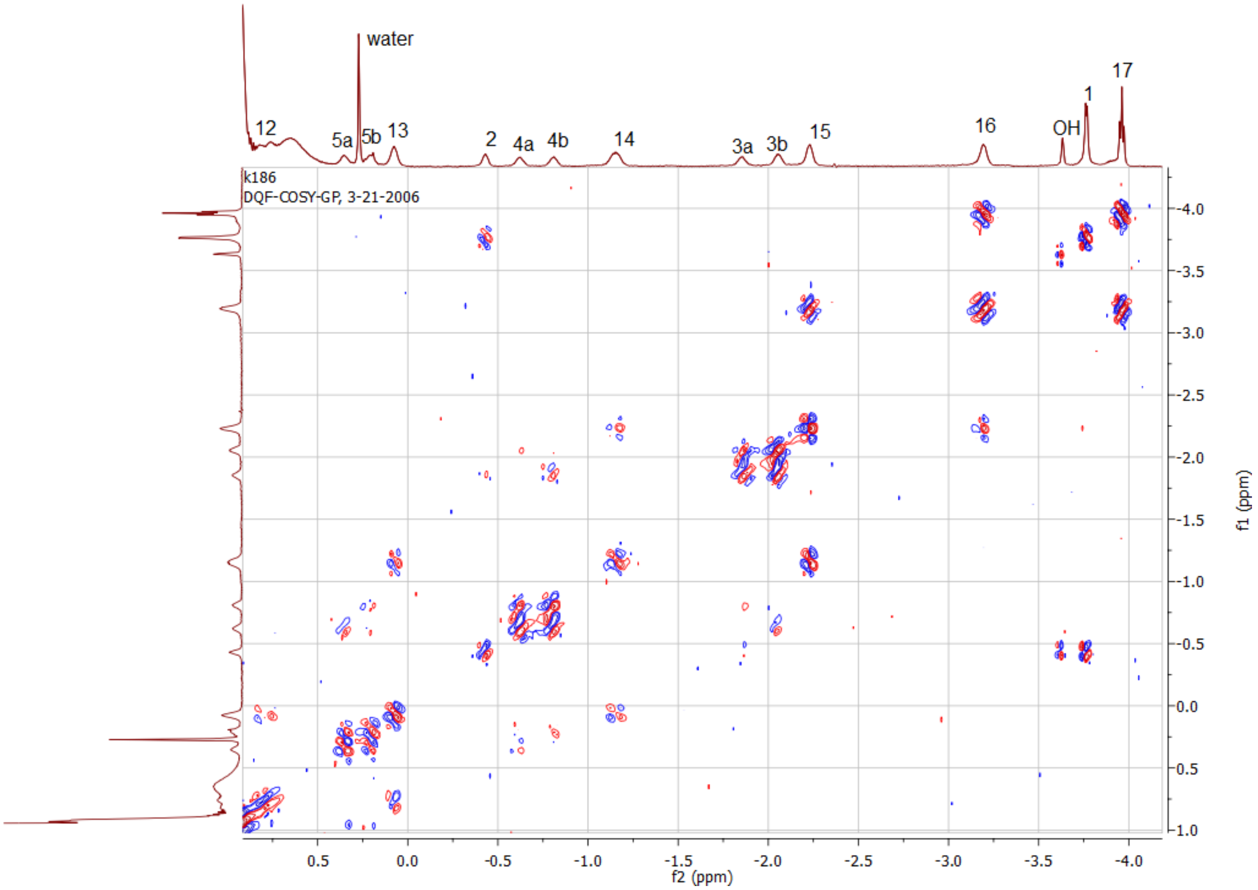

Fig. SI 13: Proton correlation (COSY) of encapsulation of 2-heptadecanol in **1.3'$_2$.3$_2$.1** (**II**).

NMR spectra of encapsulated *n*-octadecane in host II/III

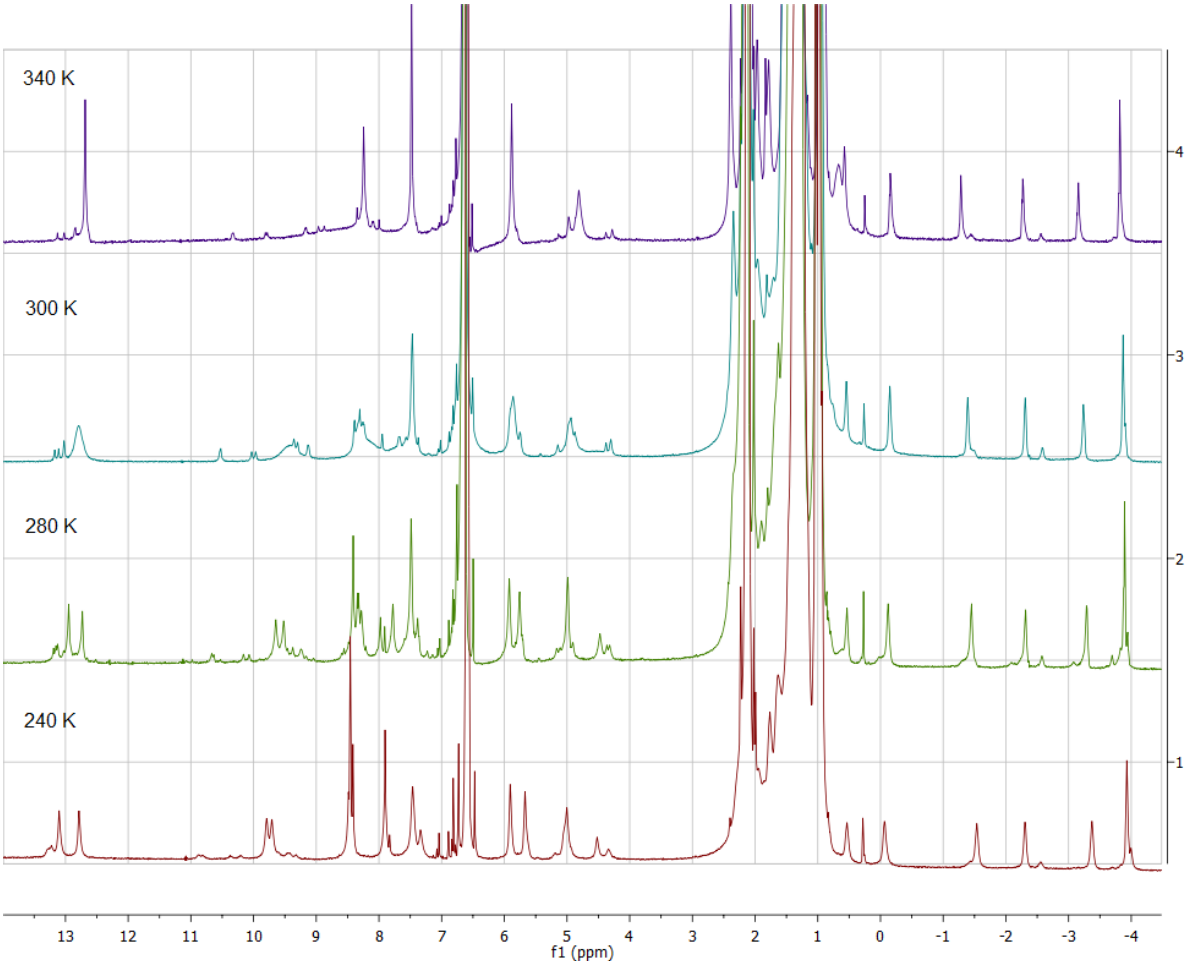

Fig. SI 14: NMR spectra of encapsulated *n*-octadecane in host II/III at 240 – 340 K.

NMR spectra of encapsulated *n*-nonadecane in host III

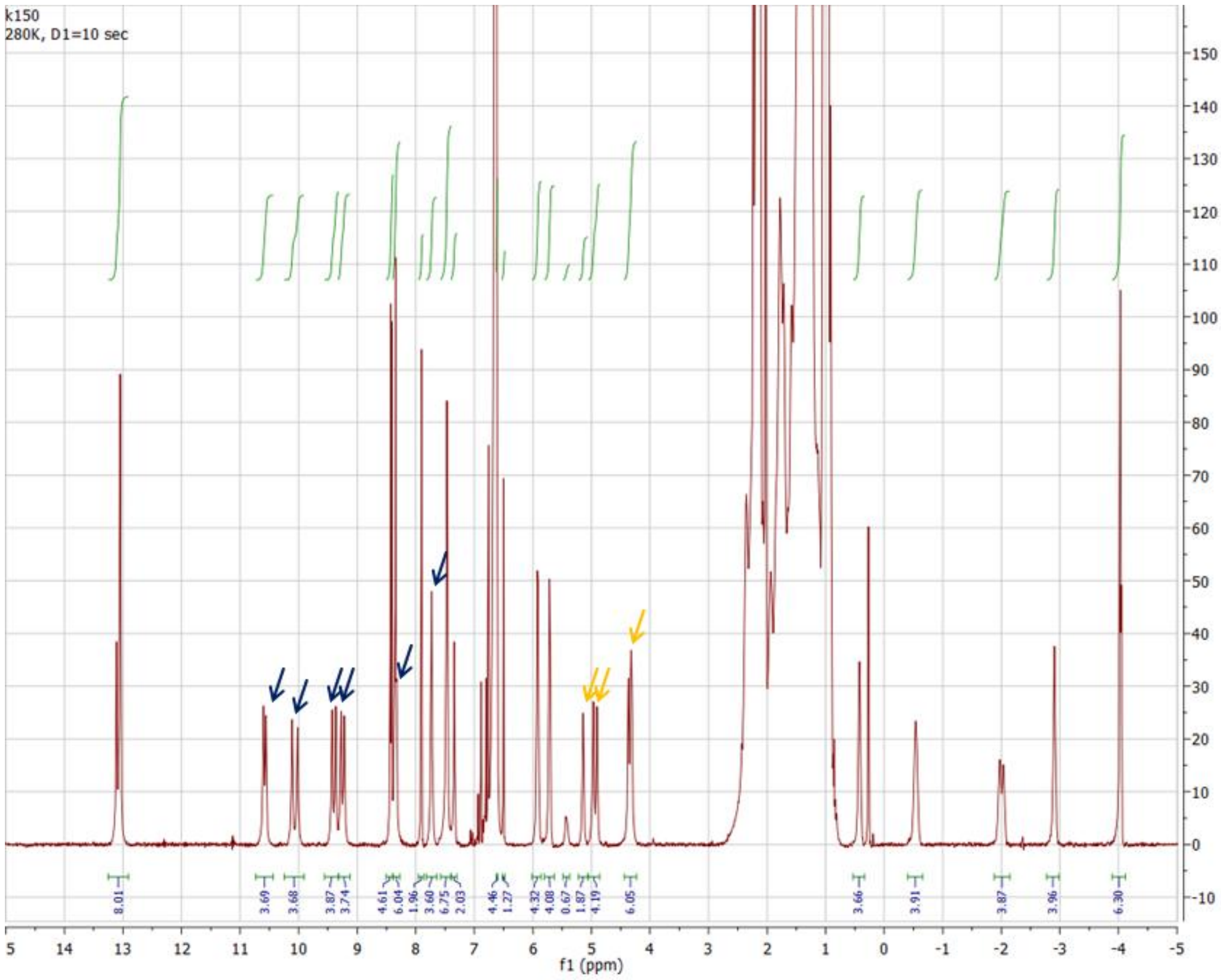

Fig. SI 15: 1H-NMR spectrum of encapsulated *n*-nonadecane. Integration of the propanediurea-NH protons (marked in blue) as well as integration of the propanediurea-bridgehead protons (marked in yellow) reveal the presence of six PD-units in the assembly **III**.

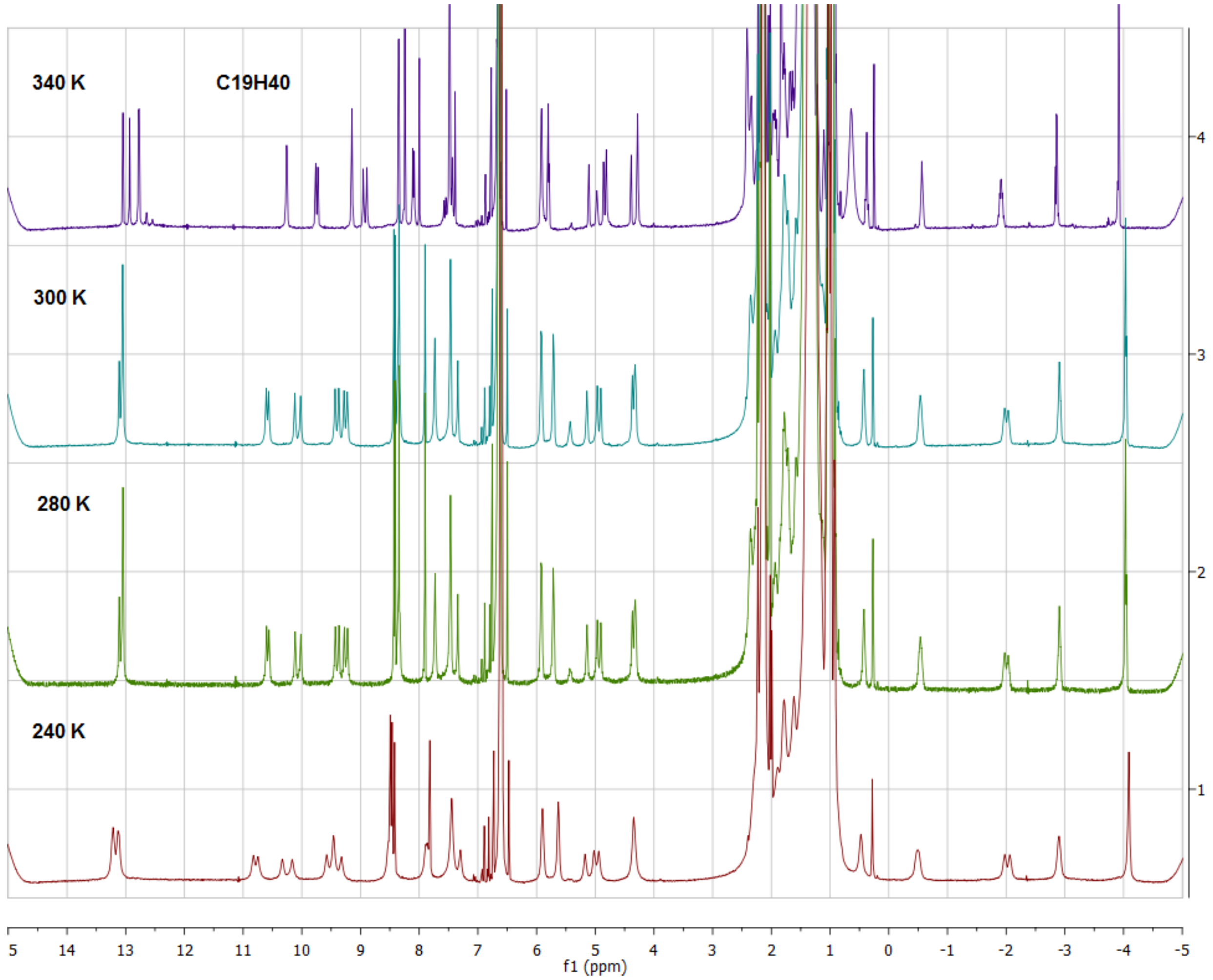

Fig. SI 16: 1H-NMR of assembly III with encapsulated *n*-nonadecane at 240 – 340 K. Diastereotopic CH2-guest signals are observed at 240-300 K, indicating a chiral assembly.

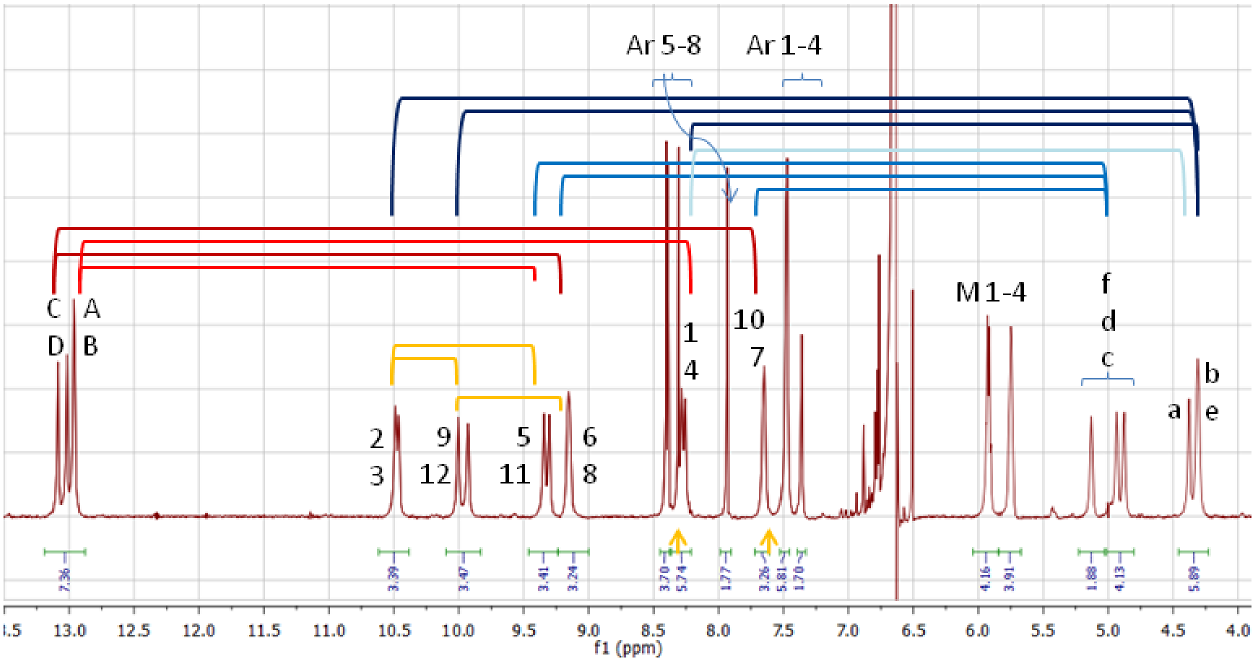

Fig. SI 17: Observed NOE-signals in assembly **III** (**1.3'$_2$.3$_4$.1**) with *n*-nonadecane as guest (cf. Fig. SI 20).

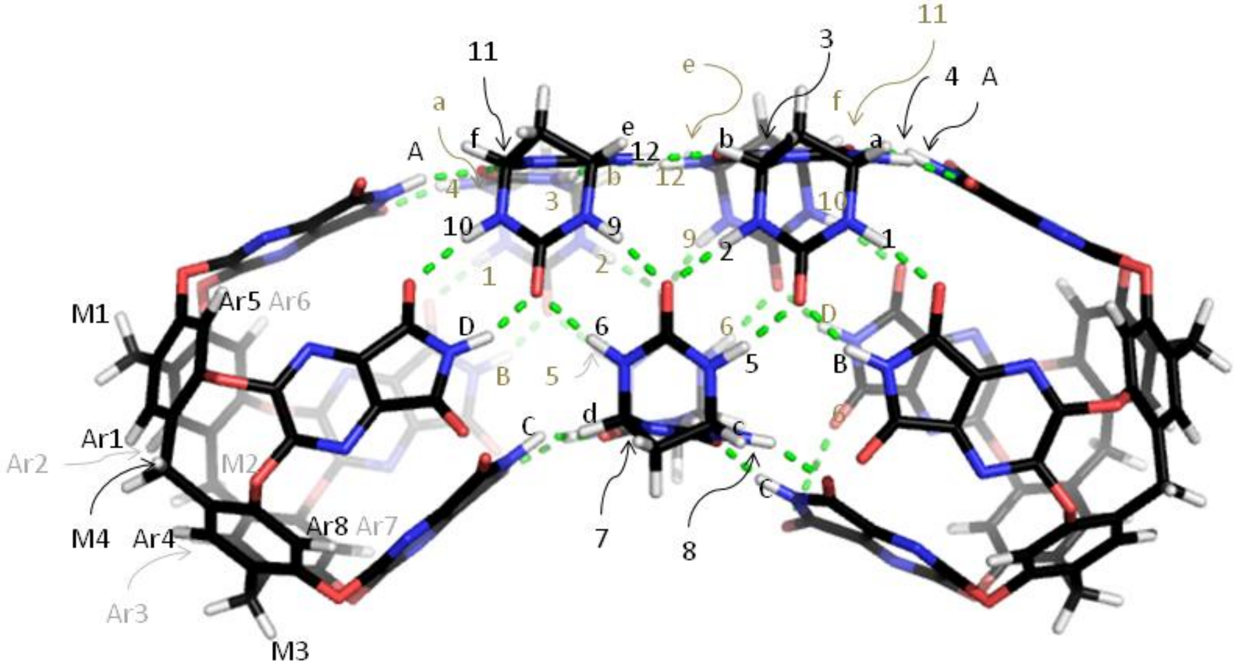

Fig. SI 18: Model of the assembly **III** (**1.3'$_2$.3$_4$.1**)- side view.

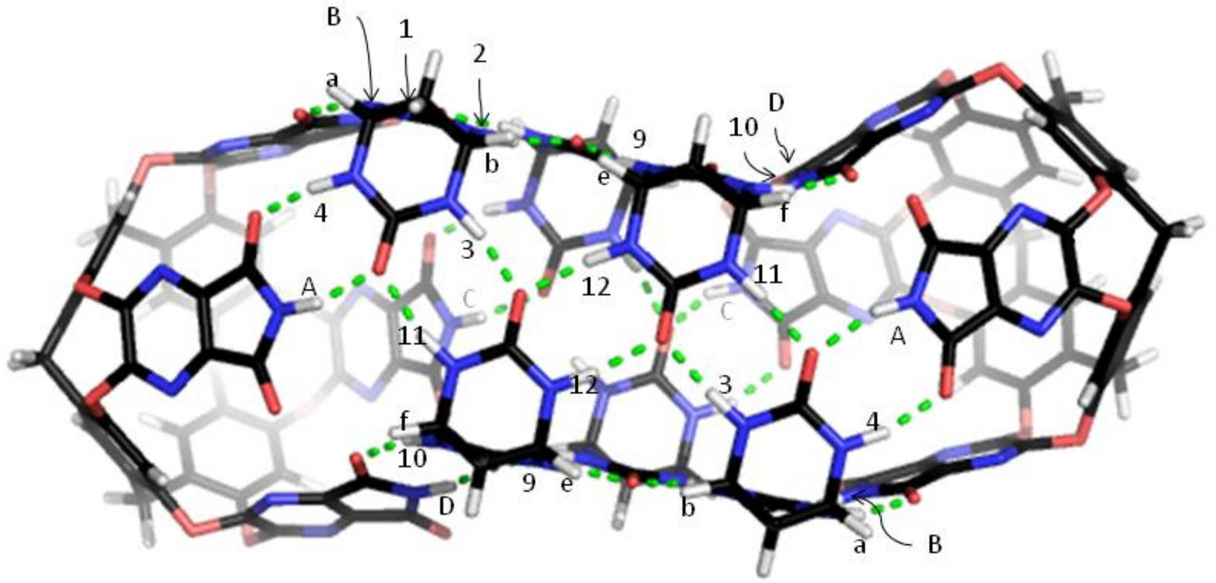

(top view)

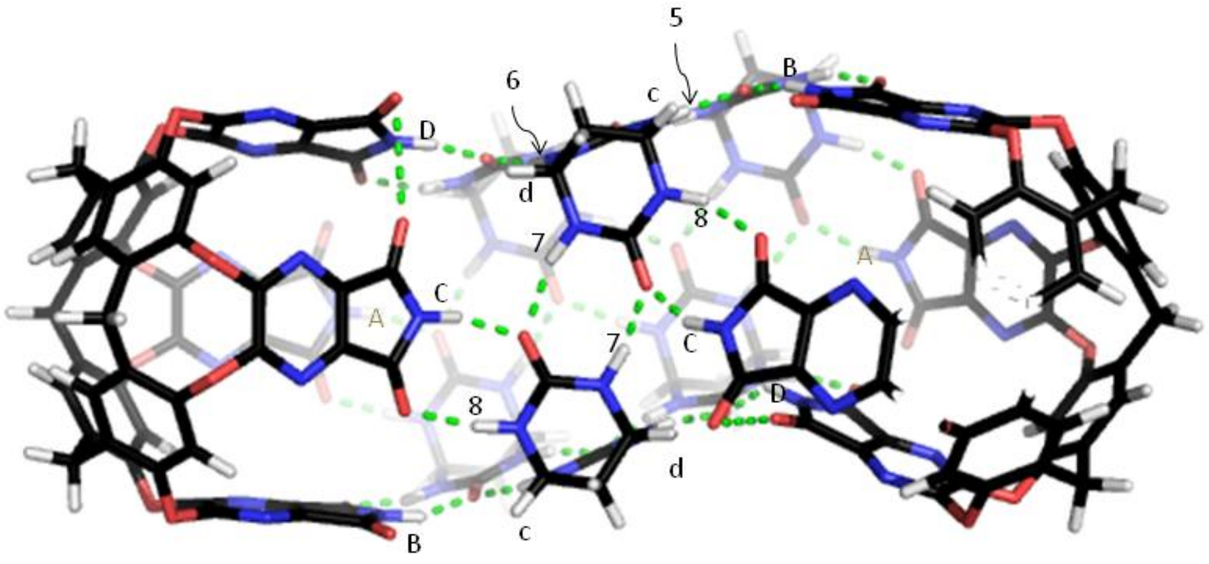

(bottom view)

Fig. SI 19: Different views of assembly **III** (**$1.3'_2.3_4.1$**).

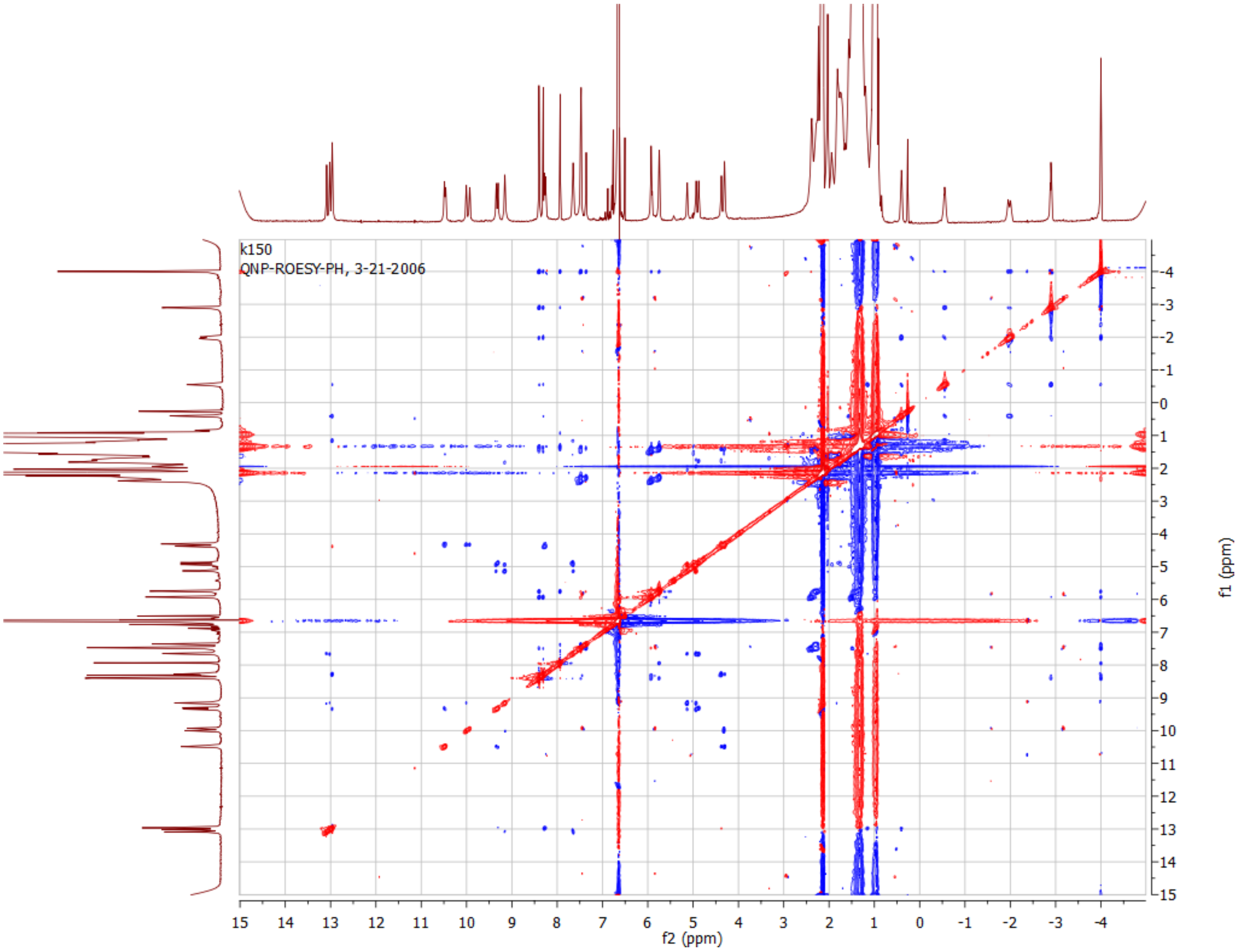

Fig. SI 20: ROESY spectrum of the assembly **III** with encapsulated *n*-nonadecane. The observed NOE-signals are displayed in Fig. SI 17.

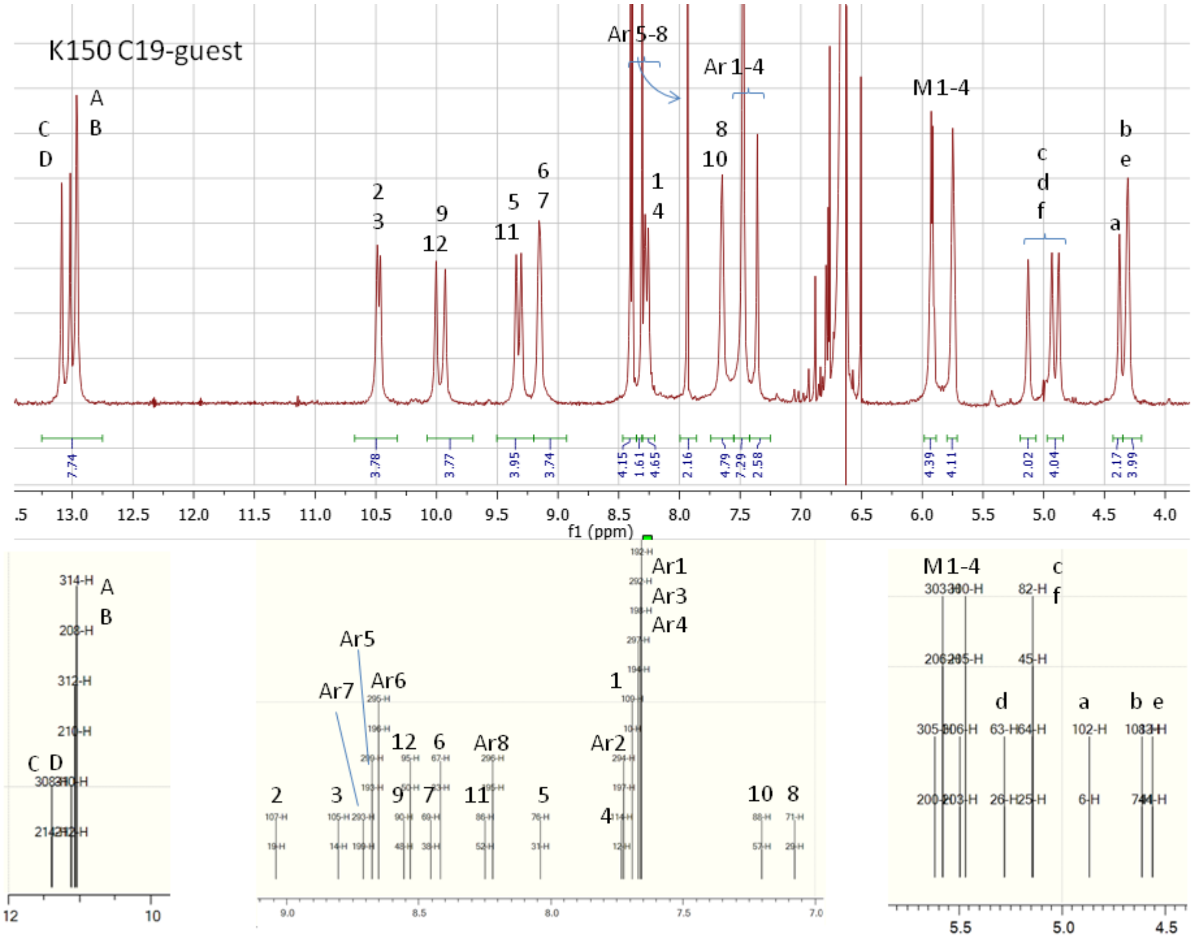

Fig. SI 21: Comparison of the observed NMR spectrum for assembly **III** encapsulating *n*-nonadecane (top) and the calculated NMR spectrum (bottom; B3LYP/6-31G*, for raw data see appendix-txt-file). To limit the degrees of freedom of the assembly all alkyl feet on cavitand **1** and on the propanediurea-units **3** had to be removed for the calculation. In addition the calculation was performed on the empty capsule (no guest present) in vacuum (no mesitylene solvent). Therefore the calculated absolute values of the resonances differ significantly from the observed ones. Nevertheless the relative orders of the imide-peaks (A-D), NH-peaks (1-12) as well as the propanediurea-bridgehead-proton signals (a-f) match well. Also the appearance and relative order of two sets of aromatic signals (Ar 5-8 and Ar 1-4) is predicted correctly by the calculation.

Shape selective encapsulation studies

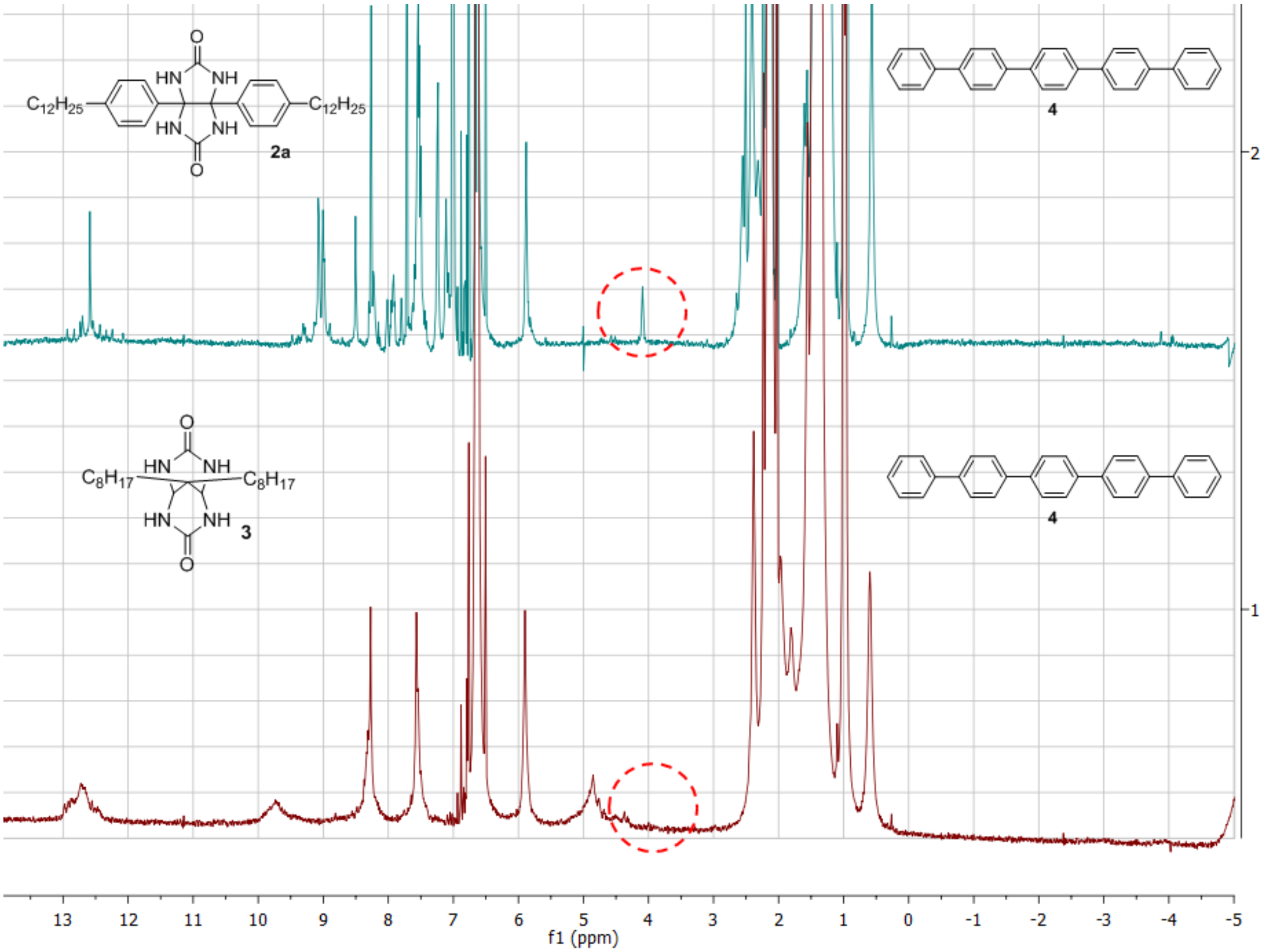

Fig. SI 22: The rigid rectilinear *p*-pentaphenyl (**4**) was accepted in the known doubly extended capsule formed by glycoluril **2a,** but it was rejected by capsule **III** assembled from propanediurea **3** apparently due to the insurmountable shape incompatibilities. The observed guest peak at ca. 4.0 ppm is indicative of the encapsulation of the aromatic guest.[5]

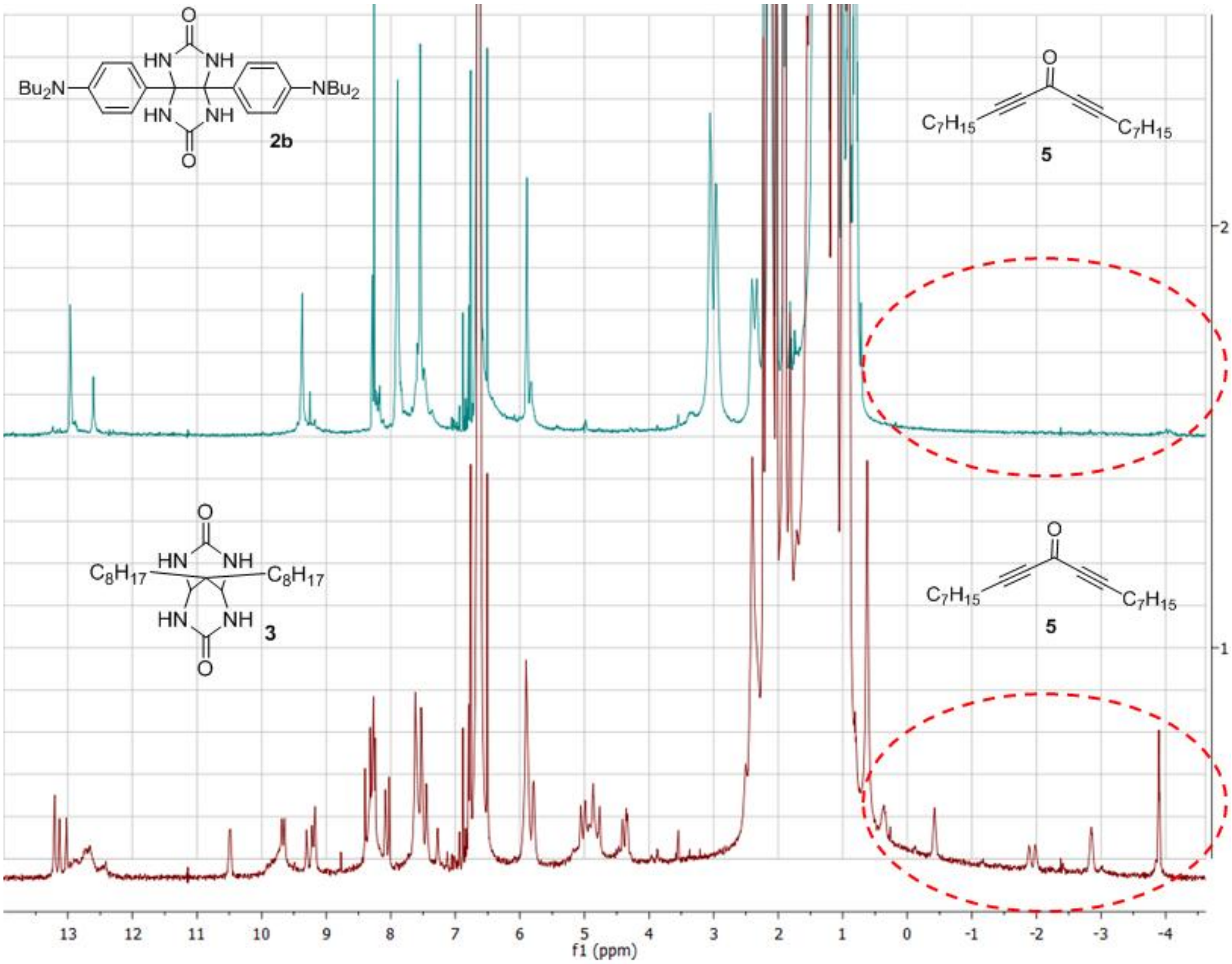

Fig. SI 23: The bent ketone-guest **5** on the other hand was encapsulated in the congruent host **III** formed by propanediurea **3** but not in the linear capsule **1.2₈.1** formed by glycoluril **2b**.

NMR spectra of encapsulated *n*-docosane (*n*-$C_{22}H_{46}$) in host IV

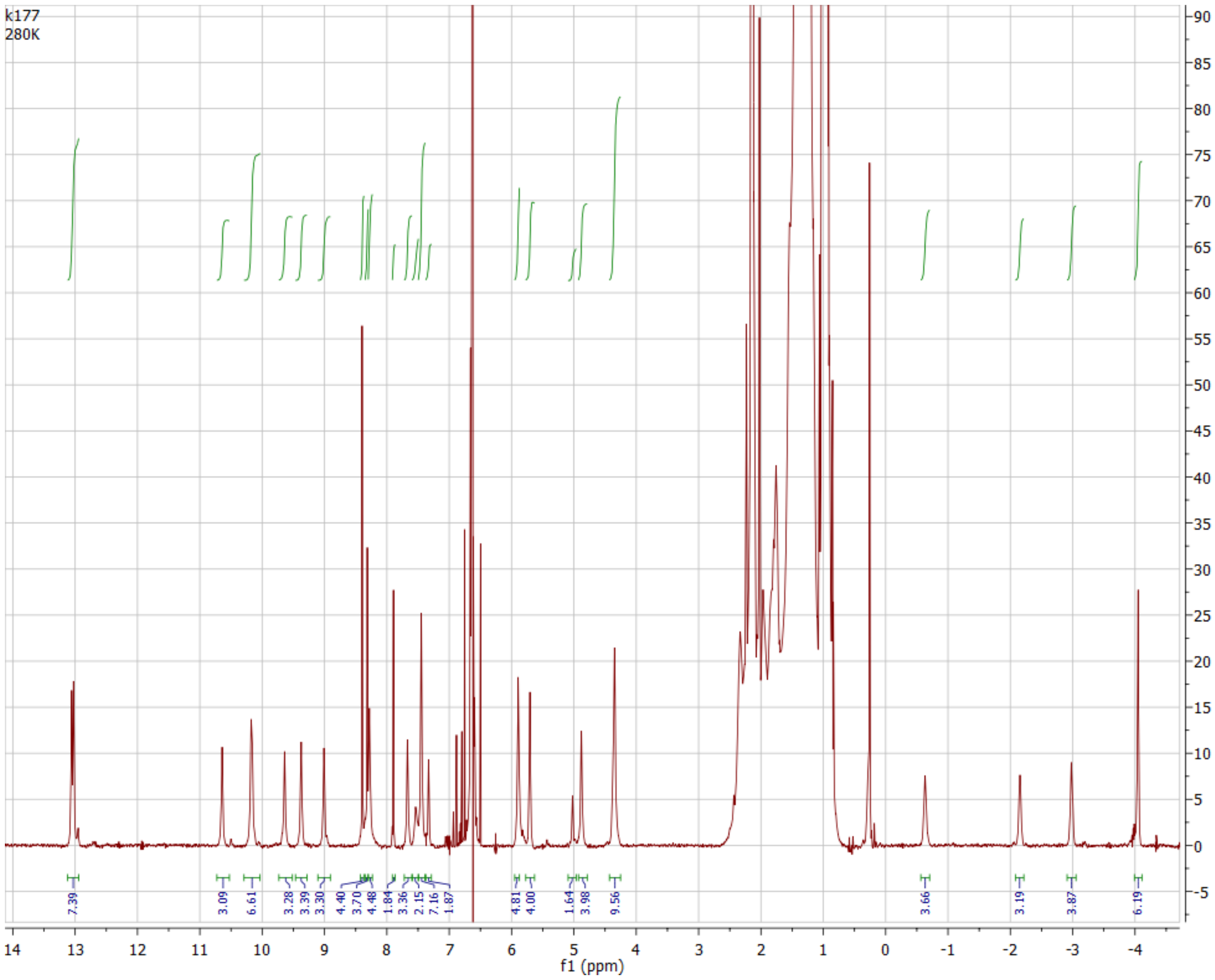

Fig. SI 24: Integrated 1H-NMR spectra of encapsulated *n*-docosane (*n*-$C_{22}H_{46}$) in host IV

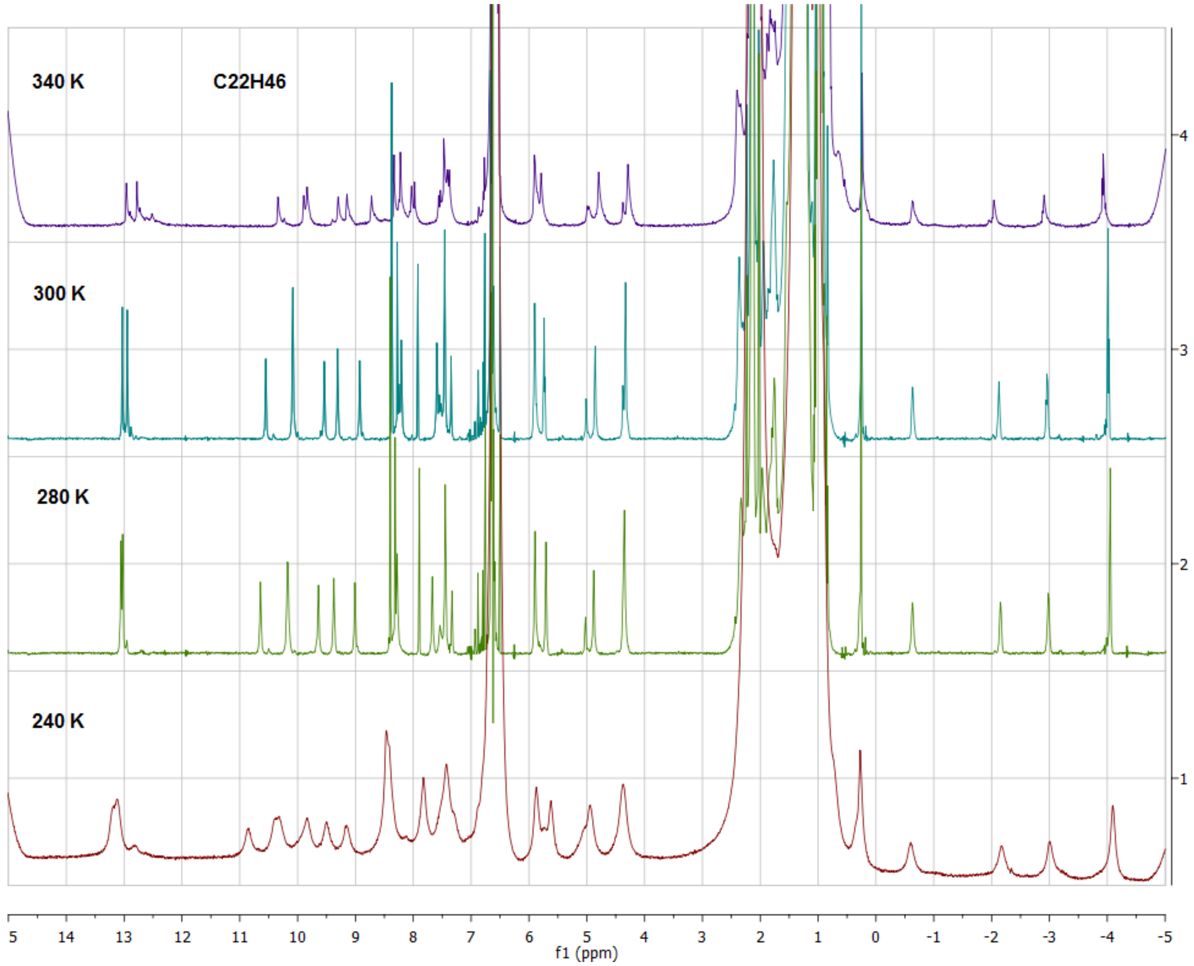

Fig. SI 25: 1H-NMR of assembly **IV** with encapsulated *n*-docosane at 240 – 340 K. No diastereotopic CH2-guest signals are displayed.

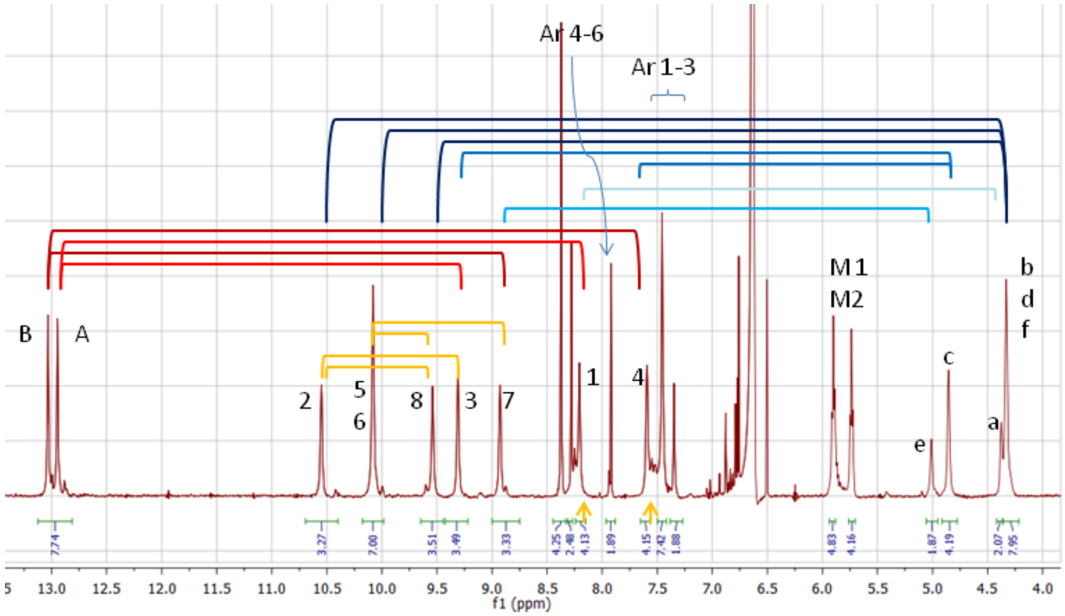

Fig. SI 26: Observed NOE-signals in assembly **IV** (**1.3'$_4$.3$_4$.1**) with *n*-docosane as guest, recorded at 300 K (cf. Fig. SI 28).

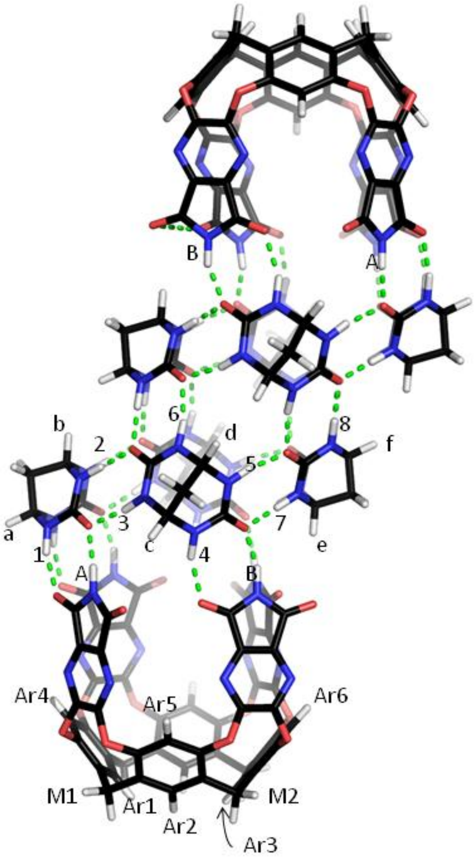

Fig. SI 27: Model of assembly **IV** (**1.3'$_4$.3$_4$.1**)-

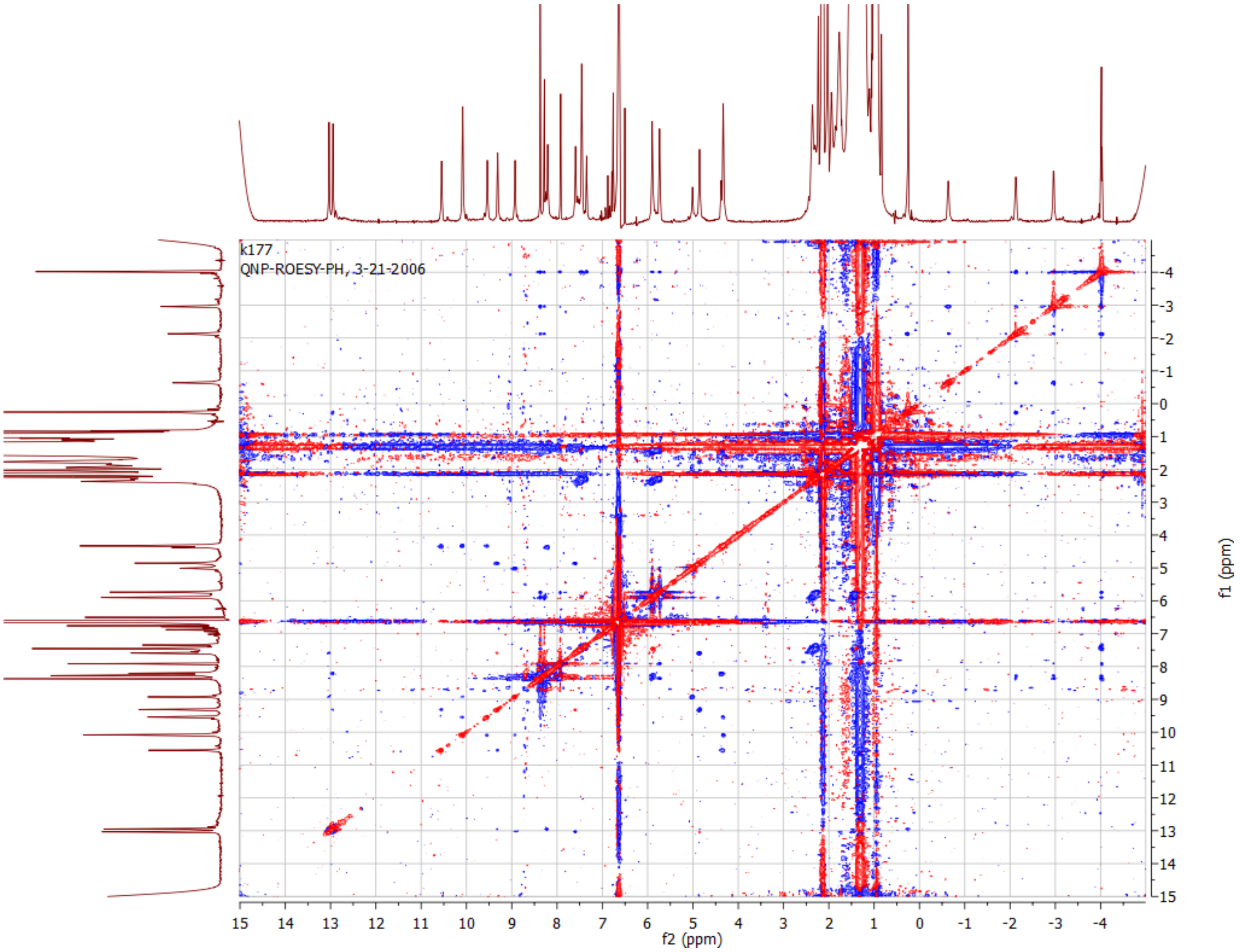

Fig. SI 28: ROESY spectrum of the assembly **IV** with encapsulated *n*-docosane. The observed NOEs were transferred to Fig. SI 26.

References

(1) Ajami, D.; Iwasawa, T.; Rebek, J. *Proc. Natl. Acad. Sci. U. S. A.* **2006**, *103*, 8934.

(2) De Haan, T. *Recl. Trav. Ch. Pays-Ba.* **1908**, *27*, 162.

(3) Jansen, R. J., PhD-thesis: Molecular clips based on propanediurea : synthesis and physical properties; University of Nijmegen, 2002.

(4) Eisler, S.; Chahal, N.; McDonald, R.; Tykwinski, R. R. *Chem.–Eur. J.* **2003**, *9*, 2542.

(5) Rebek, J.; Ajami, D. *J. Org. Chem.* **2009**, *74*, 6584.

raw data (B3LYP/6-31G*) for the calculated NMR spectrum

b3lyp/6-31g*

```
Calculating GIAO nuclear magnetic shielding tensors.
 SCF GIAO Magnetic shielding tensor (ppm):
  1  H    Isotropic =    30.0748   Anisotropy =     8.3429
   XX=    27.4798   YX=     0.4544   ZX=     1.3882
   XY=     0.2179   YY=    27.5810   ZY=     0.2688
   XZ=     2.5127   YZ=     0.0355   ZZ=    35.1636
   Eigenvalues:    26.8892    27.6985    35.6367
  2  C    Isotropic =   161.6254   Anisotropy =    26.7314
   XX=   167.6179   YX=    -1.6056   ZX=   -11.1907
   XY=    -1.8055   YY=   150.3101   ZY=     4.5296
   XZ=   -11.8891   YZ=     4.1119   ZZ=   166.9483
   Eigenvalues:   149.1517   156.2783   179.4464
  3  C    Isotropic =   131.3838   Anisotropy =    19.4443
   XX=   140.6856   YX=    -9.1312   ZX=    -1.4960
   XY=    -4.0368   YY=   111.3235   ZY=     3.0451
   XZ=    -2.9361   YZ=    -2.4642   ZZ=   142.1422
   Eigenvalues:   109.9137   139.8909   144.3466
  4  C    Isotropic =   132.1854   Anisotropy =    18.9389
   XX=   142.8616   YX=    -2.6494   ZX=    -1.2343
   XY=    -8.3305   YY=   109.9078   ZY=    -1.0705
   XZ=    -0.1738   YZ=     4.8462   ZZ=   143.7868
   Eigenvalues:   108.9293   142.8156   144.8114
  5  H    Isotropic =    30.0470   Anisotropy =     8.2669
   XX=    34.9357   YX=    -1.0388   ZX=     2.5160
   XY=    -1.1038   YY=    27.8475   ZY=    -0.2197
   XZ=     1.4254   YZ=     0.2155   ZZ=    27.3578
   Eigenvalues:    26.8063    27.7764    35.5582
  6  H    Isotropic =    27.7303   Anisotropy =     7.9720
   XX=    27.6378   YX=     3.5524   ZX=     1.5119
   XY=     3.0301   YY=    29.1539   ZY=     2.2469
   XZ=     1.6177   YZ=     2.6866   ZZ=    26.3993
   Eigenvalues:    24.7819    25.3641    33.0449
  7  H    Isotropic =    27.9865   Anisotropy =     7.4520
   XX=    25.5037   YX=    -0.5375   ZX=     0.5394
   XY=    -0.5487   YY=    32.3475   ZY=    -1.8917
   XZ=     0.4296   YZ=    -1.9706   ZZ=    26.1084
   Eigenvalues:    25.1814    25.8237    32.9545
  8  N    Isotropic =   145.6124   Anisotropy =    61.7987
   XX=   170.5029   YX=   -35.9949   ZX=    -4.6342
   XY=     1.2949   YY=   151.7612   ZY=   -27.0725
   XZ=    -6.4310   YZ=   -53.0357   ZZ=   114.5730
   Eigenvalues:    86.6288   163.3968   186.8115
  9  N    Isotropic =   147.6027   Anisotropy =    54.2502
   XX=   176.6182   YX=    23.6295   ZX=     2.0252
   XY=    -9.8162   YY=   144.1774   ZY=    27.7715
   XZ=     5.4358   YZ=    53.7713   ZZ=   122.0125
   Eigenvalues:    90.8263   168.2123   183.7695
 10  H    Isotropic =    24.9050   Anisotropy =    15.0771
   XX=    15.6856   YX=     0.9699   ZX=    -0.8185
   XY=    -0.5932   YY=    31.4498   ZY=    -6.0364
   XZ=    -0.2350   YZ=    -4.1127   ZZ=    27.5796
   Eigenvalues:    15.6622    24.0964    34.9564
```

```
11  N    Isotropic =   147.0721   Anisotropy =    55.1357
  XX=   139.6579   YX=    55.5659   ZX=    -6.6536
  XY=    29.4925   YY=   129.2329   ZY=    -2.0842
  XZ=     2.1026   YZ=    31.3984   ZZ=   172.3256
  Eigenvalues:    89.6949   167.6923   183.8293
12  H    Isotropic =    24.8645   Anisotropy =    15.0363
  XX=    25.6543   YX=    -2.9743   ZX=    -0.0895
  XY=    -4.9486   YY=    32.7409   ZY=    -3.7293
  XZ=    -0.0642   YZ=    -2.1270   ZZ=    16.1985
  Eigenvalues:    15.6346    24.0702    34.8887
13  N    Isotropic =   145.3835   Anisotropy =    60.8235
  XX=    99.7326   YX=   -43.8771   ZX=     3.8359
  XY=   -18.4659   YY=   159.9294   ZY=     5.3242
  XZ=    -6.7007   YZ=   -29.9201   ZZ=   176.4886
  Eigenvalues:    86.0790   164.1390   185.9325
14  H    Isotropic =    23.7926   Anisotropy =    19.4566
  XX=    29.3498   YX=     7.0637   ZX=    -1.0472
  XY=     7.6758   YY=    29.2534   ZY=    -0.1971
  XZ=    -2.2480   YZ=    -0.7016   ZZ=    12.7747
  Eigenvalues:    12.6069    22.0073    36.7637
15  C    Isotropic =    41.0695   Anisotropy =   103.9982
  XX=    23.8298   YX=    -8.7727   ZX=     7.7592
  XY=    -9.9515   YY=   -10.9275   ZY=    -0.1452
  XZ=    -3.8928   YZ=     5.5785   ZZ=   110.3062
  Eigenvalues:   -13.3669    26.1738   110.4016
16  C    Isotropic =    41.2857   Anisotropy =   103.2870
  XX=   106.1610   YX=   -19.9908   ZX=    -4.0868
  XY=   -23.4398   YY=    -9.9717   ZY=    -1.5229
  XZ=     7.6698   YZ=    -2.6168   ZZ=    27.6679
  Eigenvalues:   -13.9703    27.6838   110.1437
17  O    Isotropic =    97.2042   Anisotropy =   281.6395
  XX=   272.8841   YX=   -49.2852   ZX=   -21.7183
  XY=   -62.6773   YY=    21.9073   ZY=     6.5937
  XZ=    36.1454   YZ=    -3.8815   ZZ=    -3.1786
  Eigenvalues:    -3.9131    10.5620   284.9639
18  O    Isotropic =    99.6019   Anisotropy =   276.0413
  XX=     3.3594   YX=     4.7758   ZX=    29.4179
  XY=     3.2553   YY=    11.8718   ZY=   -13.5468
  XZ=   -22.5823   YZ=     9.6452   ZZ=   283.5744
  Eigenvalues:     1.7102    13.4660   283.6294
19  H    Isotropic =    23.5569   Anisotropy =    20.3394
  XX=    12.7325   YX=     2.5219   ZX=    -0.6255
  XY=     3.2700   YY=    32.0233   ZY=     7.7514
  XZ=     0.2783   YZ=     6.8768   ZZ=    25.9150
  Eigenvalues:    12.1708    21.3834    37.1166
20  H    Isotropic =    30.0175   Anisotropy =     7.8328
  XX=    34.4342   YX=    -1.7175   ZX=     2.1049
  XY=    -1.8306   YY=    28.6098   ZY=    -0.3592
  XZ=     1.0115   YZ=    -0.3069   ZZ=    27.0084
  Eigenvalues:    26.6943    28.1188    35.2393
21  C    Isotropic =   161.8933   Anisotropy =    32.0137
  XX=   167.0350   YX=    -4.1537   ZX=   -13.9315
  XY=    -4.6134   YY=   149.2672   ZY=     2.6995
  XZ=   -14.6083   YZ=     2.5791   ZZ=   169.3777
```

```
  Eigenvalues:   148.2020   154.2421   183.2358
22  C    Isotropic =   132.5075   Anisotropy =    19.2598
  XX=   142.1375   YX=   -10.8208   ZX=    -1.1942
  XY=    -6.3126   YY=   111.4855   ZY=    -0.6553
  XZ=    -2.5763   YZ=    -5.5370   ZZ=   143.8996
  Eigenvalues:   108.9090   143.2662   145.3474
23  C    Isotropic =   133.4167   Anisotropy =    20.2245
  XX=   144.3014   YX=    -4.0864   ZX=    -1.8817
  XY=    -8.8243   YY=   111.0399   ZY=    -1.4021
  XZ=    -1.0888   YZ=     3.5371   ZZ=   144.9087
  Eigenvalues:   109.8138   143.5365   146.8997
24  H    Isotropic =    30.0891   Anisotropy =     7.3960
  XX=    27.1362   YX=    -0.2958   ZX=     0.9810
  XY=    -0.2627   YY=    28.5357   ZY=    -0.2621
  XZ=     2.5144   YZ=    -0.5913   ZZ=    34.5954
  Eigenvalues:    26.7289    28.5187    35.0198
25  H    Isotropic =    27.4527   Anisotropy =     7.9851
  XX=    26.3591   YX=     3.5183   ZX=     0.7256
  XY=     2.6159   YY=    30.0388   ZY=     2.2878
  XZ=     1.1509   YZ=     2.6433   ZZ=    25.9602
  Eigenvalues:    24.3815    25.2005    32.7761
26  H    Isotropic =    27.3174   Anisotropy =     9.4011
  XX=    24.4728   YX=    -2.1358   ZX=     0.0416
  XY=    -1.4029   YY=    32.2374   ZY=    -2.5004
  XZ=    -0.0305   YZ=    -3.2854   ZZ=    25.2421
  Eigenvalues:    23.5992    24.7683    33.5849
27  N    Isotropic =   146.1511   Anisotropy =    63.0311
  XX=   101.6927   YX=   -43.7312   ZX=     5.9608
  XY=   -19.5672   YY=   161.2907   ZY=     5.3595
  XZ=    -1.9275   YZ=   -31.7114   ZZ=   175.4698
  Eigenvalues:    87.8887   162.3927   188.1718
28  N    Isotropic =   150.4020   Anisotropy =    64.0132
  XX=   149.1241   YX=    54.9299   ZX=     7.3569
  XY=    29.4785   YY=   127.5493   ZY=   -12.4526
  XZ=    14.5846   YZ=    31.1446   ZZ=   174.5326
  Eigenvalues:    94.7702   163.3583   193.0775
29  H    Isotropic =    25.5201   Anisotropy =    15.7879
  XX=    24.9475   YX=    -1.0491   ZX=    -3.3615
  XY=    -3.0146   YY=    33.0402   ZY=     5.0033
  XZ=    -4.4007   YZ=     6.6038   ZZ=    18.5725
  Eigenvalues:    15.5287    24.9862    36.0453
30  N    Isotropic =   148.4378   Anisotropy =    56.3780
  XX=   179.1578   YX=    23.1504   ZX=     7.1402
  XY=   -13.6515   YY=   142.8781   ZY=    28.1270
  XZ=    11.2285   YZ=    48.8596   ZZ=   123.2774
  Eigenvalues:    93.1350   166.1552   186.0231
31  H    Isotropic =    24.5577   Anisotropy =    19.1074
  XX=    14.6404   YX=     5.4783   ZX=    -3.1923
  XY=     4.0225   YY=    31.7239   ZY=    -6.2787
  XZ=    -2.1293   YZ=    -5.9129   ZZ=    27.3089
  Eigenvalues:    13.3157    23.0614    37.2960
32  N    Isotropic =   147.7825   Anisotropy =    62.4105
  XX=   170.6461   YX=   -37.8439   ZX=    -5.1870
  XY=     1.3306   YY=   154.9392   ZY=   -27.8043
```

```
  XZ=    -5.1293   YZ=   -50.6606   ZZ=   117.7622
  Eigenvalues:    90.3958   163.5623   189.3895
33  H    Isotropic =    24.1798   Anisotropy =    19.5897
  XX=    13.0730   YX=     0.1394   ZX=    -0.8620
  XY=     1.4874   YY=    32.2987   ZY=     7.6118
  XZ=     0.3279   YZ=     6.4738   ZZ=    27.1678
  Eigenvalues:    13.0112    22.2886    37.2396
34  C    Isotropic =    41.8601   Anisotropy =   103.7644
  XX=   106.0749   YX=   -22.9937   ZX=    -1.0451
  XY=   -24.5279   YY=    -7.3748   ZY=    -1.9804
  XZ=     7.9234   YZ=    -3.9495   ZZ=    26.8803
  Eigenvalues:   -12.2776    26.8216   111.0364
35  C    Isotropic =    42.9548   Anisotropy =   102.6525
  XX=    24.8030   YX=   -10.5722   ZX=    13.1012
  XY=    -9.5225   YY=    -5.3937   ZY=    -8.6892
  XZ=     5.0618   YZ=   -10.5164   ZZ=   109.4551
  Eigenvalues:    -8.8069    26.2815   111.3898
36  O    Isotropic =   113.6744   Anisotropy =   258.7396
  XX=    27.1159   YX=    -0.9709   ZX=    54.2319
  XY=     3.0523   YY=    29.4976   ZY=   -19.0642
  XZ=   -18.4851   YZ=    -4.2865   ZZ=   284.4097
  Eigenvalues:    25.0223    29.8334   286.1674
37  O    Isotropic =   111.5501   Anisotropy =   274.3942
  XX=   283.2734   YX=   -50.9530   ZX=   -30.1504
  XY=   -56.9572   YY=    33.1804   ZY=     4.8874
  XZ=    38.0774   YZ=    -7.5854   ZZ=    18.1966
  Eigenvalues:    18.0662    22.1046   294.4796
38  H    Isotropic =    24.1429   Anisotropy =    18.7700
  XX=    30.1100   YX=     6.3170   ZX=    -1.9018
  XY=     7.1921   YY=    28.7346   ZY=    -1.3657
  XZ=    -3.2430   YZ=    -2.4911   ZZ=    13.5839
  Eigenvalues:    13.1308    22.6416    36.6562
39  H    Isotropic =    30.1638   Anisotropy =     8.0576
  XX=    27.2900   YX=    -0.0707   ZX=    -0.9025
  XY=    -0.1916   YY=    27.9043   ZY=     0.0976
  XZ=    -1.8458   YZ=     0.3932   ZZ=    35.2972
  Eigenvalues:    27.0513    27.9046    35.5355
40  C    Isotropic =   161.3276   Anisotropy =    30.9131
  XX=   168.8867   YX=    -4.3288   ZX=    13.4103
  XY=    -4.2472   YY=   148.8359   ZY=    -3.4193
  XZ=    13.5190   YZ=    -2.7672   ZZ=   166.2602
  Eigenvalues:   147.9461   154.1003   181.9363
41  C    Isotropic =   132.4058   Anisotropy =    18.1411
  XX=   141.4883   YX=   -10.2514   ZX=     0.6201
  XY=    -5.1630   YY=   111.8317   ZY=    -1.8430
  XZ=     1.4670   YZ=     3.6530   ZZ=   143.8975
  Eigenvalues:   109.9110   142.8066   144.4999
42  C    Isotropic =   132.7569   Anisotropy =    19.5506
  XX=   144.0216   YX=    -2.3851   ZX=     1.6931
  XY=    -7.4887   YY=   109.6262   ZY=     1.5726
  XZ=     0.2874   YZ=    -3.4568   ZZ=   144.6230
  Eigenvalues:   108.9139   143.5662   145.7906
43  H    Isotropic =    30.2171   Anisotropy =     7.7940
  XX=    34.4485   YX=    -1.6324   ZX=    -2.7480
```

```
  XY=    -1.6933   YY=    28.4309   ZY=     0.3013
  XZ=    -1.3324   YZ=     0.0628   ZZ=    27.7717
  Eigenvalues:    27.1194    28.1188    35.4131
44  H    Isotropic =    28.0374   Anisotropy =     7.7987
  XX=    27.0269   YX=     3.0786   ZX=    -1.2190
  XY=     2.7080   YY=    30.8690   ZY=    -1.9218
  XZ=    -1.1882   YZ=    -2.2177   ZZ=    26.2164
  Eigenvalues:    25.3516    25.5241    33.2366
45  H    Isotropic =    27.4554   Anisotropy =     8.0185
  XX=    25.1677   YX=    -1.5609   ZX=    -0.0974
  XY=    -1.0870   YY=    31.9507   ZY=     1.7747
  XZ=     0.3585   YZ=     2.6007   ZZ=    25.2478
  Eigenvalues:    24.2526    25.3126    32.8011
46  N    Isotropic =   150.0519   Anisotropy =    64.2426
  XX=   168.6279   YX=   -36.3554   ZX=    -1.9954
  XY=     6.0465   YY=   152.9037   ZY=    30.5325
  XZ=     1.2795   YZ=    57.5663   ZZ=   128.6239
  Eigenvalues:    93.9878   163.2875   192.8802
47  N    Isotropic =   146.5642   Anisotropy =    59.6109
  XX=   177.0722   YX=    21.4138   ZX=   -11.6747
  XY=   -14.0672   YY=   143.3027   ZY=   -26.6961
  XZ=   -15.1863   YZ=   -51.4106   ZZ=   119.3175
  Eigenvalues:    89.5936   163.7942   186.3047
48  H    Isotropic =    24.0409   Anisotropy =    18.9016
  XX=    14.4016   YX=     4.9811   ZX=     3.8886
  XY=     3.8148   YY=    31.1912   ZY=     6.2296
  XZ=     2.9368   YZ=     5.6760   ZZ=    26.5299
  Eigenvalues:    13.0019    22.4788    36.6420
49  N    Isotropic =   148.0340   Anisotropy =    59.3702
  XX=   136.8360   YX=    51.6604   ZX=    -0.1503
  XY=    30.7585   YY=   131.1223   ZY=     5.0572
  XZ=    -6.2253   YZ=   -32.1169   ZZ=   176.1437
  Eigenvalues:    91.9496   164.5383   187.6141
50  H    Isotropic =    24.0655   Anisotropy =    19.5909
  XX=    24.2403   YX=    -4.6989   ZX=     0.6655
  XY=    -5.4318   YY=    35.0574   ZY=    -0.3636
  XZ=     1.4875   YZ=    -1.5264   ZZ=    12.8988
  Eigenvalues:    12.7866    22.2838    37.1261
51  N    Isotropic =   148.1389   Anisotropy =    57.5130
  XX=   106.3543   YX=   -40.1982   ZX=    -0.3267
  XY=   -19.7177   YY=   162.4447   ZY=    -5.1567
  XZ=     7.6037   YZ=    31.3135   ZZ=   175.6176
  Eigenvalues:    92.4709   165.4649   186.4808
52  H    Isotropic =    24.3485   Anisotropy =    19.1149
  XX=    30.2700   YX=     6.7306   ZX=     0.7342
  XY=     7.3555   YY=    29.6452   ZY=    -0.0184
  XZ=     2.0109   YZ=     1.2570   ZZ=    13.1303
  Eigenvalues:    13.0209    22.9329    37.0918
53  C    Isotropic =    42.1137   Anisotropy =   103.1256
  XX=    27.2546   YX=    -8.7764   ZX=    -5.5474
  XY=    -8.0180   YY=   -11.7190   ZY=     3.3650
  XZ=     4.5609   YZ=     1.7449   ZZ=   110.8054
  Eigenvalues:   -13.4977    28.9747   110.8641
54  C    Isotropic =    43.3399   Anisotropy =   102.2552
```

```
  XX=   106.7831   YX=   -16.1249   ZX=    -8.0450
  XY=   -20.6273   YY=    -7.0340   ZY=     0.8124
  XZ=   -16.2167   YZ=     2.0404   ZZ=    30.2708
  Eigenvalues:    -9.9332    28.4430   111.5101
55  O    Isotropic =   108.8461   Anisotropy =   268.2230
  XX=   275.1916   YX=   -46.9501   ZX=     8.3839
  XY=   -50.8543   YY=    33.0914   ZY=     1.9736
  XZ=   -63.2662   YZ=    11.9248   ZZ=    18.2552
  Eigenvalues:    15.0005    23.8764   287.6614
56  O    Isotropic =   106.9825   Anisotropy =   283.5864
  XX=     9.0726   YX=     1.0911   ZX=   -28.1246
  XY=     1.8298   YY=    15.9725   ZY=    10.8830
  XZ=    36.6075   YZ=    -1.7566   ZZ=   295.9023
  Eigenvalues:     8.7390    16.1684   296.0401
57  H    Isotropic =    25.3957   Anisotropy =    15.7407
  XX=    17.2614   YX=    -2.3860   ZX=     4.8472
  XY=    -1.1782   YY=    33.0652   ZY=    -5.6439
  XZ=     3.9775   YZ=    -3.2774   ZZ=    25.8605
  Eigenvalues:    15.3997    24.8980    35.8895
58  H    Isotropic =    30.0891   Anisotropy =     7.3960
  XX=    27.1362   YX=    -0.2958   ZX=    -0.9810
  XY=    -0.2627   YY=    28.5357   ZY=     0.2621
  XZ=    -2.5144   YZ=     0.5913   ZZ=    34.5954
  Eigenvalues:    26.7289    28.5187    35.0198
59  C    Isotropic =   161.8933   Anisotropy =    32.0137
  XX=   167.0350   YX=    -4.1537   ZX=    13.9315
  XY=    -4.6134   YY=   149.2672   ZY=    -2.6995
  XZ=    14.6083   YZ=    -2.5791   ZZ=   169.3777
  Eigenvalues:   148.2020   154.2421   183.2358
60  C    Isotropic =   133.4167   Anisotropy =    20.2245
  XX=   144.3014   YX=    -4.0864   ZX=     1.8817
  XY=    -8.8243   YY=   111.0399   ZY=     1.4021
  XZ=     1.0888   YZ=    -3.5371   ZZ=   144.9087
  Eigenvalues:   109.8138   143.5365   146.8997
61  C    Isotropic =   132.5075   Anisotropy =    19.2598
  XX=   142.1375   YX=   -10.8208   ZX=     1.1942
  XY=    -6.3126   YY=   111.4855   ZY=     0.6553
  XZ=     2.5763   YZ=     5.5370   ZZ=   143.8996
  Eigenvalues:   108.9090   143.2662   145.3474
62  H    Isotropic =    30.0175   Anisotropy =     7.8328
  XX=    34.4342   YX=    -1.7175   ZX=    -2.1049
  XY=    -1.8306   YY=    28.6098   ZY=     0.3592
  XZ=    -1.0115   YZ=     0.3069   ZZ=    27.0084
  Eigenvalues:    26.6943    28.1188    35.2393
63  H    Isotropic =    27.3174   Anisotropy =     9.4011
  XX=    24.4728   YX=    -2.1358   ZX=    -0.0416
  XY=    -1.4029   YY=    32.2374   ZY=     2.5004
  XZ=     0.0305   YZ=     3.2854   ZZ=    25.2421
  Eigenvalues:    23.5992    24.7683    33.5849
64  H    Isotropic =    27.4527   Anisotropy =     7.9851
  XX=    26.3591   YX=     3.5183   ZX=    -0.7256
  XY=     2.6159   YY=    30.0388   ZY=    -2.2878
  XZ=    -1.1509   YZ=    -2.6433   ZZ=    25.9602
  Eigenvalues:    24.3815    25.2005    32.7761
```

```
65  N    Isotropic =   148.4378   Anisotropy =    56.3780
  XX=   179.1578   YX=    23.1504   ZX=    -7.1402
  XY=   -13.6515   YY=   142.8781   ZY=   -28.1270
  XZ=   -11.2285   YZ=   -48.8596   ZZ=   123.2774
  Eigenvalues:    93.1350   166.1552   186.0231
66  N    Isotropic =   147.7825   Anisotropy =    62.4105
  XX=   170.6461   YX=   -37.8439   ZX=     5.1870
  XY=     1.3306   YY=   154.9392   ZY=    27.8043
  XZ=     5.1293   YZ=    50.6606   ZZ=   117.7622
  Eigenvalues:    90.3958   163.5623   189.3895
67  H    Isotropic =    24.1798   Anisotropy =    19.5897
  XX=    13.0730   YX=     0.1394   ZX=     0.8620
  XY=     1.4874   YY=    32.2987   ZY=    -7.6118
  XZ=    -0.3279   YZ=    -6.4738   ZZ=    27.1678
  Eigenvalues:    13.0112    22.2886    37.2396
68  N    Isotropic =   146.1511   Anisotropy =    63.0311
  XX=   101.6927   YX=   -43.7312   ZX=    -5.9608
  XY=   -19.5672   YY=   161.2907   ZY=    -5.3595
  XZ=     1.9275   YZ=    31.7114   ZZ=   175.4698
  Eigenvalues:    87.8887   162.3927   188.1718
69  H    Isotropic =    24.1429   Anisotropy =    18.7700
  XX=    30.1100   YX=     6.3170   ZX=     1.9018
  XY=     7.1921   YY=    28.7346   ZY=     1.3657
  XZ=     3.2430   YZ=     2.4911   ZZ=    13.5839
  Eigenvalues:    13.1308    22.6416    36.6562
70  N    Isotropic =   150.4020   Anisotropy =    64.0132
  XX=   149.1241   YX=    54.9299   ZX=    -7.3569
  XY=    29.4785   YY=   127.5493   ZY=    12.4526
  XZ=   -14.5846   YZ=   -31.1446   ZZ=   174.5326
  Eigenvalues:    94.7702   163.3583   193.0775
71  H    Isotropic =    25.5201   Anisotropy =    15.7879
  XX=    24.9475   YX=    -1.0491   ZX=     3.3615
  XY=    -3.0146   YY=    33.0402   ZY=    -5.0033
  XZ=     4.4007   YZ=    -6.6038   ZZ=    18.5725
  Eigenvalues:    15.5287    24.9862    36.0453
72  C    Isotropic =    42.9548   Anisotropy =   102.6525
  XX=    24.8030   YX=   -10.5722   ZX=   -13.1012
  XY=    -9.5225   YY=    -5.3937   ZY=     8.6892
  XZ=    -5.0618   YZ=    10.5164   ZZ=   109.4551
  Eigenvalues:    -8.8069    26.2815   111.3898
73  C    Isotropic =    41.8601   Anisotropy =   103.7644
  XX=   106.0749   YX=   -22.9938   ZX=     1.0451
  XY=   -24.5279   YY=    -7.3748   ZY=     1.9804
  XZ=    -7.9234   YZ=     3.9495   ZZ=    26.8803
  Eigenvalues:   -12.2776    26.8216   111.0364
74  O    Isotropic =   111.5501   Anisotropy =   274.3942
  XX=   283.2734   YX=   -50.9530   ZX=    30.1504
  XY=   -56.9572   YY=    33.1804   ZY=    -4.8874
  XZ=   -38.0774   YZ=     7.5854   ZZ=    18.1966
  Eigenvalues:    18.0662    22.1046   294.4796
75  O    Isotropic =   113.6744   Anisotropy =   258.7396
  XX=    27.1159   YX=    -0.9709   ZX=   -54.2319
  XY=     3.0523   YY=    29.4976   ZY=    19.0642
  XZ=    18.4851   YZ=     4.2865   ZZ=   284.4097
```

```
  Eigenvalues:    25.0223    29.8334   286.1674
76  H    Isotropic =    24.5577   Anisotropy =    19.1074
  XX=    14.6404   YX=     5.4783   ZX=     3.1923
  XY=     4.0225   YY=    31.7239   ZY=     6.2787
  XZ=     2.1293   YZ=     5.9129   ZZ=    27.3089
  Eigenvalues:    13.3157    23.0614    37.2960
77  H    Isotropic =    30.2171   Anisotropy =     7.7940
  XX=    34.4485   YX=    -1.6324   ZX=     2.7480
  XY=    -1.6933   YY=    28.4309   ZY=    -0.3013
  XZ=     1.3324   YZ=    -0.0628   ZZ=    27.7717
  Eigenvalues:    27.1194    28.1188    35.4131
78  C    Isotropic =   161.3276   Anisotropy =    30.9131
  XX=   168.8867   YX=    -4.3288   ZX=   -13.4103
  XY=    -4.2472   YY=   148.8359   ZY=     3.4193
  XZ=   -13.5190   YZ=     2.7672   ZZ=   166.2602
  Eigenvalues:   147.9461   154.1003   181.9363
79  C    Isotropic =   132.7569   Anisotropy =    19.5506
  XX=   144.0216   YX=    -2.3851   ZX=    -1.6931
  XY=    -7.4887   YY=   109.6262   ZY=    -1.5726
  XZ=    -0.2874   YZ=     3.4568   ZZ=   144.6230
  Eigenvalues:   108.9139   143.5662   145.7906
80  C    Isotropic =   132.4058   Anisotropy =    18.1411
  XX=   141.4883   YX=   -10.2514   ZX=    -0.6201
  XY=    -5.1630   YY=   111.8317   ZY=     1.8430
  XZ=    -1.4670   YZ=    -3.6530   ZZ=   143.8975
  Eigenvalues:   109.9110   142.8066   144.4999
81  H    Isotropic =    30.1638   Anisotropy =     8.0576
  XX=    27.2900   YX=    -0.0707   ZX=     0.9025
  XY=    -0.1916   YY=    27.9043   ZY=    -0.0976
  XZ=     1.8458   YZ=    -0.3932   ZZ=    35.2972
  Eigenvalues:    27.0513    27.9046    35.5355
82  H    Isotropic =    27.4554   Anisotropy =     8.0185
  XX=    25.1677   YX=    -1.5609   ZX=     0.0974
  XY=    -1.0870   YY=    31.9507   ZY=    -1.7747
  XZ=    -0.3585   YZ=    -2.6007   ZZ=    25.2478
  Eigenvalues:    24.2526    25.3126    32.8011
83  H    Isotropic =    28.0374   Anisotropy =     7.7987
  XX=    27.0269   YX=     3.0786   ZX=     1.2190
  XY=     2.7080   YY=    30.8690   ZY=     1.9218
  XZ=     1.1882   YZ=     2.2177   ZZ=    26.2164
  Eigenvalues:    25.3516    25.5241    33.2366
84  N    Isotropic =   148.0340   Anisotropy =    59.3702
  XX=   136.8360   YX=    51.6604   ZX=     0.1503
  XY=    30.7585   YY=   131.1223   ZY=    -5.0572
  XZ=     6.2253   YZ=    32.1169   ZZ=   176.1437
  Eigenvalues:    91.9496   164.5383   187.6141
85  N    Isotropic =   148.1389   Anisotropy =    57.5130
  XX=   106.3543   YX=   -40.1982   ZX=     0.3267
  XY=   -19.7177   YY=   162.4447   ZY=     5.1567
  XZ=    -7.6037   YZ=   -31.3135   ZZ=   175.6176
  Eigenvalues:    92.4709   165.4649   186.4808
86  H    Isotropic =    24.3485   Anisotropy =    19.1149
  XX=    30.2700   YX=     6.7306   ZX=    -0.7342
  XY=     7.3555   YY=    29.6452   ZY=     0.0184
```

```
  XZ=    -2.0109   YZ=    -1.2570   ZZ=    13.1303
  Eigenvalues:    13.0209    22.9329    37.0918
 87  N    Isotropic =   150.0519   Anisotropy =    64.2426
  XX=   168.6279   YX=   -36.3554   ZX=     1.9954
  XY=     6.0465   YY=   152.9037   ZY=   -30.5325
  XZ=    -1.2795   YZ=   -57.5663   ZZ=   128.6239
  Eigenvalues:    93.9878   163.2875   192.8802
 88  H    Isotropic =    25.3957   Anisotropy =    15.7407
  XX=    17.2614   YX=    -2.3860   ZX=    -4.8472
  XY=    -1.1782   YY=    33.0652   ZY=     5.6439
  XZ=    -3.9775   YZ=     3.2774   ZZ=    25.8605
  Eigenvalues:    15.3997    24.8980    35.8895
 89  N    Isotropic =   146.5642   Anisotropy =    59.6109
  XX=   177.0722   YX=    21.4138   ZX=    11.6747
  XY=   -14.0672   YY=   143.3027   ZY=    26.6961
  XZ=    15.1863   YZ=    51.4106   ZZ=   119.3175
  Eigenvalues:    89.5936   163.7942   186.3047
 90  H    Isotropic =    24.0409   Anisotropy =    18.9016
  XX=    14.4016   YX=     4.9811   ZX=    -3.8886
  XY=     3.8148   YY=    31.1912   ZY=    -6.2296
  XZ=    -2.9368   YZ=    -5.6760   ZZ=    26.5299
  Eigenvalues:    13.0019    22.4788    36.6420
 91  C    Isotropic =    43.3399   Anisotropy =   102.2552
  XX=   106.7831   YX=   -16.1249   ZX=     8.0450
  XY=   -20.6273   YY=    -7.0340   ZY=    -0.8124
  XZ=    16.2167   YZ=    -2.0404   ZZ=    30.2708
  Eigenvalues:    -9.9332    28.4430   111.5101
 92  C    Isotropic =    42.1137   Anisotropy =   103.1256
  XX=    27.2546   YX=    -8.7764   ZX=     5.5474
  XY=    -8.0180   YY=   -11.7190   ZY=    -3.3650
  XZ=    -4.5609   YZ=    -1.7449   ZZ=   110.8054
  Eigenvalues:   -13.4977    28.9747   110.8641
 93  O    Isotropic =   106.9825   Anisotropy =   283.5864
  XX=     9.0726   YX=     1.0911   ZX=    28.1246
  XY=     1.8298   YY=    15.9725   ZY=   -10.8830
  XZ=   -36.6075   YZ=     1.7566   ZZ=   295.9023
  Eigenvalues:     8.7390    16.1684   296.0401
 94  O    Isotropic =   108.8461   Anisotropy =   268.2230
  XX=   275.1916   YX=   -46.9501   ZX=    -8.3839
  XY=   -50.8543   YY=    33.0914   ZY=    -1.9736
  XZ=    63.2662   YZ=   -11.9248   ZZ=    18.2552
  Eigenvalues:    15.0005    23.8764   287.6614
 95  H    Isotropic =    24.0655   Anisotropy =    19.5909
  XX=    24.2403   YX=    -4.6989   ZX=    -0.6655
  XY=    -5.4318   YY=    35.0574   ZY=     0.3636
  XZ=    -1.4875   YZ=     1.5264   ZZ=    12.8988
  Eigenvalues:    12.7866    22.2838    37.1261
 96  H    Isotropic =    30.0470   Anisotropy =     8.2669
  XX=    34.9357   YX=    -1.0388   ZX=    -2.5160
  XY=    -1.1038   YY=    27.8475   ZY=     0.2197
  XZ=    -1.4254   YZ=    -0.2155   ZZ=    27.3578
  Eigenvalues:    26.8063    27.7764    35.5582
 97  C    Isotropic =   161.6254   Anisotropy =    26.7314
  XX=   167.6179   YX=    -1.6056   ZX=    11.1907
```

```
   XY=    -1.8055   YY=   150.3101   ZY=    -4.5296
   XZ=    11.8891   YZ=    -4.1119   ZZ=   166.9483
   Eigenvalues:   149.1517   156.2783   179.4464
 98  C    Isotropic =   132.1854   Anisotropy =    18.9389
   XX=   142.8616   YX=    -2.6494   ZX=     1.2343
   XY=    -8.3305   YY=   109.9078   ZY=     1.0705
   XZ=     0.1738   YZ=    -4.8462   ZZ=   143.7868
   Eigenvalues:   108.9293   142.8156   144.8114
 99  C    Isotropic =   131.3838   Anisotropy =    19.4443
   XX=   140.6856   YX=    -9.1312   ZX=     1.4960
   XY=    -4.0368   YY=   111.3235   ZY=    -3.0451
   XZ=     2.9361   YZ=     2.4642   ZZ=   142.1422
   Eigenvalues:   109.9137   139.8909   144.3466
100  H    Isotropic =    30.0748   Anisotropy =     8.3429
   XX=    27.4798   YX=     0.4544   ZX=    -1.3882
   XY=     0.2179   YY=    27.5810   ZY=    -0.2688
   XZ=    -2.5127   YZ=    -0.0355   ZZ=    35.1636
   Eigenvalues:    26.8892    27.6985    35.6367
101  H    Isotropic =    27.9865   Anisotropy =     7.4520
   XX=    25.5037   YX=    -0.5375   ZX=    -0.5394
   XY=    -0.5487   YY=    32.3475   ZY=     1.8917
   XZ=    -0.4296   YZ=     1.9706   ZZ=    26.1084
   Eigenvalues:    25.1814    25.8237    32.9545
102  H    Isotropic =    27.7303   Anisotropy =     7.9720
   XX=    27.6378   YX=     3.5524   ZX=    -1.5119
   XY=     3.0301   YY=    29.1539   ZY=    -2.2469
   XZ=    -1.6177   YZ=    -2.6866   ZZ=    26.3993
   Eigenvalues:    24.7819    25.3641    33.0449
103  N    Isotropic =   147.0721   Anisotropy =    55.1357
   XX=   139.6579   YX=    55.5659   ZX=     6.6536
   XY=    29.4925   YY=   129.2329   ZY=     2.0842
   XZ=    -2.1026   YZ=   -31.3984   ZZ=   172.3256
   Eigenvalues:    89.6949   167.6923   183.8293
104  N    Isotropic =   145.3835   Anisotropy =    60.8235
   XX=    99.7326   YX=   -43.8771   ZX=    -3.8359
   XY=   -18.4659   YY=   159.9294   ZY=    -5.3242
   XZ=     6.7007   YZ=    29.9201   ZZ=   176.4886
   Eigenvalues:    86.0790   164.1390   185.9325
105  H    Isotropic =    23.7926   Anisotropy =    19.4566
   XX=    29.3498   YX=     7.0637   ZX=     1.0472
   XY=     7.6758   YY=    29.2534   ZY=     0.1971
   XZ=     2.2480   YZ=     0.7016   ZZ=    12.7747
   Eigenvalues:    12.6069    22.0073    36.7637
106  N    Isotropic =   145.6124   Anisotropy =    61.7987
   XX=   170.5029   YX=   -35.9949   ZX=     4.6342
   XY=     1.2949   YY=   151.7612   ZY=    27.0725
   XZ=     6.4310   YZ=    53.0357   ZZ=   114.5730
   Eigenvalues:    86.6288   163.3968   186.8115
107  H    Isotropic =    23.5569   Anisotropy =    20.3394
   XX=    12.7325   YX=     2.5219   ZX=     0.6255
   XY=     3.2700   YY=    32.0233   ZY=    -7.7514
   XZ=    -0.2783   YZ=    -6.8768   ZZ=    25.9150
   Eigenvalues:    12.1708    21.3834    37.1166
108  N    Isotropic =   147.6027   Anisotropy =    54.2502
```

```
   XX=   176.6182   YX=    23.6295   ZX=    -2.0252
   XY=    -9.8162   YY=   144.1774   ZY=   -27.7715
   XZ=    -5.4358   YZ=   -53.7713   ZZ=   122.0125
   Eigenvalues:    90.8263   168.2123   183.7695
109  H    Isotropic =    24.9050   Anisotropy =    15.0771
   XX=    15.6856   YX=     0.9699   ZX=     0.8185
   XY=    -0.5932   YY=    31.4498   ZY=     6.0364
   XZ=     0.2350   YZ=     4.1127   ZZ=    27.5796
   Eigenvalues:    15.6622    24.0964    34.9564
110  C    Isotropic =    41.2857   Anisotropy =   103.2870
   XX=   106.1610   YX=   -19.9908   ZX=     4.0868
   XY=   -23.4398   YY=    -9.9717   ZY=     1.5229
   XZ=    -7.6698   YZ=     2.6168   ZZ=    27.6679
   Eigenvalues:   -13.9703    27.6838   110.1437
111  C    Isotropic =    41.0695   Anisotropy =   103.9982
   XX=    23.8298   YX=    -8.7727   ZX=    -7.7592
   XY=    -9.9515   YY=   -10.9275   ZY=     0.1452
   XZ=     3.8928   YZ=    -5.5785   ZZ=   110.3062
   Eigenvalues:   -13.3669    26.1738   110.4016
112  O    Isotropic =    99.6019   Anisotropy =   276.0413
   XX=     3.3594   YX=     4.7758   ZX=   -29.4179
   XY=     3.2553   YY=    11.8718   ZY=    13.5468
   XZ=    22.5823   YZ=    -9.6452   ZZ=   283.5744
   Eigenvalues:     1.7102    13.4660   283.6294
113  O    Isotropic =    97.2042   Anisotropy =   281.6395
   XX=   272.8841   YX=   -49.2852   ZX=    21.7183
   XY=   -62.6773   YY=    21.9073   ZY=    -6.5937
   XZ=   -36.1454   YZ=     3.8815   ZZ=    -3.1786
   Eigenvalues:    -3.9131    10.5620   284.9639
114  H    Isotropic =    24.8645   Anisotropy =    15.0363
   XX=    25.6543   YX=    -2.9743   ZX=     0.0895
   XY=    -4.9486   YY=    32.7409   ZY=     3.7293
   XZ=     0.0642   YZ=     2.1270   ZZ=    16.1985
   Eigenvalues:    15.6346    24.0702    34.8887
115  C    Isotropic =    65.1561   Anisotropy =   126.0875
   XX=   102.7067   YX=   -49.3099   ZX=    -8.6504
   XY=   -47.0411   YY=    62.8914   ZY=    53.6315
   XZ=   -10.0234   YZ=    57.8415   ZZ=    29.8702
   Eigenvalues:   -16.1847    62.4386   149.2144
116  C    Isotropic =    62.0648   Anisotropy =   152.6754
   XX=    79.7222   YX=   -79.5710   ZX=   -35.3881
   XY=   -69.0788   YY=    62.4805   ZY=    32.8698
   XZ=   -42.2197   YZ=    19.2316   ZZ=    43.9917
   Eigenvalues:    -4.8875    27.2335   163.8485
117  C    Isotropic =    42.5346   Anisotropy =    95.2229
   XX=    59.5912   YX=   -29.0395   ZX=   -47.9885
   XY=   -23.7985   YY=    73.5624   ZY=     8.3674
   XZ=   -41.6524   YZ=     9.6054   ZZ=    -5.5500
   Eigenvalues:   -28.5511    50.1383   106.0165
118  C    Isotropic =    74.9658   Anisotropy =   130.9378
   XX=   104.9210   YX=   -58.5779   ZX=   -13.2413
   XY=   -51.6960   YY=    81.9948   ZY=    40.1984
   XZ=   -17.0965   YZ=    45.1026   ZZ=    37.9815
   Eigenvalues:     8.2630    54.3766   162.2577
```

```
119  C    Isotropic =    42.0999   Anisotropy =    97.0281
   XX=    45.4977   YX=   -65.6673   ZX=   -16.0832
   XY=   -64.8797   YY=    26.5999   ZY=     2.4957
   XZ=   -20.7808   YZ=     3.0435   ZZ=    54.2022
   Eigenvalues:   -31.0963    50.6108   106.7854
120  C    Isotropic =    61.1818   Anisotropy =   151.7790
   XX=    89.5552   YX=   -52.7894   ZX=   -50.5405
   XY=   -64.2630   YY=    74.6471   ZY=    18.4590
   XZ=   -50.0016   YZ=    33.4828   ZZ=    19.3432
   Eigenvalues:    -7.2001    28.3778   162.3678
121  C    Isotropic =    62.5296   Anisotropy =   153.7000
   XX=    66.5899   YX=   -34.8934   ZX=    82.9051
   XY=   -18.3036   YY=    31.3006   ZY=   -23.4596
   XZ=    76.3261   YZ=    -7.7179   ZZ=    89.6983
   Eigenvalues:    -5.4223    28.0147   164.9963
122  C    Isotropic =    62.5010   Anisotropy =   131.0571
   XX=    91.6746   YX=   -38.8559   ZX=    41.5316
   XY=   -35.5432   YY=    -8.1997   ZY=   -20.1043
   XZ=    40.3266   YZ=   -23.3020   ZZ=   104.0282
   Eigenvalues:   -21.0822    58.7127   149.8724
123  C    Isotropic =    62.8259   Anisotropy =   153.4143
   XX=    86.5027   YX=    -4.8419   ZX=    72.8384
   XY=   -19.5699   YY=    24.5453   ZY=   -25.4320
   XZ=    78.1088   YZ=   -42.9465   ZZ=    77.4297
   Eigenvalues:    -5.2964    28.6720   165.1021
124  C    Isotropic =    41.3883   Anisotropy =    96.3946
   XX=    35.3885   YX=   -35.1490   ZX=    56.0293
   XY=   -29.1971   YY=    40.2031   ZY=     5.1106
   XZ=    61.3219   YZ=     8.1401   ZZ=    48.5732
   Eigenvalues:   -29.0712    47.5846   105.6513
125  C    Isotropic =    74.2387   Anisotropy =   131.2398
   XX=    97.2202   YX=   -29.8477   ZX=    49.7282
   XY=   -30.2579   YY=    17.5007   ZY=   -21.7318
   XZ=    49.6667   YZ=   -21.6819   ZZ=   107.9953
   Eigenvalues:     7.1185    53.8657   161.7319
126  C    Isotropic =    41.2940   Anisotropy =    96.7713
   XX=    54.3125   YX=    15.2680   ZX=    50.4049
   XY=    11.6291   YY=    18.3247   ZY=   -38.5317
   XZ=    45.5382   YZ=   -44.1160   ZZ=    51.2449
   Eigenvalues:   -29.2551    47.3290   105.8082
127  C    Isotropic =    75.9951   Anisotropy =   130.3573
   XX=    54.1189   YX=    51.6115   ZX=   -13.0892
   XY=    45.2759   YY=   136.3979   ZY=    26.9425
   XZ=   -11.1727   YZ=    33.3205   ZZ=    37.4686
   Eigenvalues:     9.2362    55.8492   162.9000
128  C    Isotropic =    42.2216   Anisotropy =    94.9645
   XX=    51.5254   YX=    17.7379   ZX=    27.7442
   XY=    14.5945   YY=    94.4264   ZY=    19.2716
   XZ=    22.4131   YZ=    18.9717   ZZ=   -19.2871
   Eigenvalues:   -28.7710    49.9044   105.5312
129  C    Isotropic =    61.8068   Anisotropy =   152.5349
   XX=    25.2521   YX=    64.0283   ZX=    15.5701
   XY=    54.3293   YY=   129.2041   ZY=    30.6786
   XZ=    28.2533   YZ=    18.5722   ZZ=    30.9642
```

```
   Eigenvalues:    -4.7855    26.7091   163.4967
130  C    Isotropic =    63.7200   Anisotropy =   129.2133
   XX=    45.8627   YX=    54.0662   ZX=   -24.3764
   XY=    50.6533   YY=   117.3625   ZY=    37.2095
   XZ=   -24.0255   YZ=    40.5965   ZZ=    27.9347
   Eigenvalues:   -18.2588    59.5565   149.8622
131  C    Isotropic =    61.5844   Anisotropy =   153.2047
   XX=    49.0443   YX=    46.4350   ZX=    29.1247
   XY=    55.4389   YY=   131.6895   ZY=    21.3257
   XZ=    16.9610   YZ=    34.5884   ZZ=     4.0194
   Eigenvalues:    -6.1000    27.1324   163.7209
132  C    Isotropic =    41.9773   Anisotropy =    95.9046
   XX=    -4.7467   YX=    46.5793   ZX=    18.3244
   XY=    47.6394   YY=    83.8504   ZY=     0.4796
   XZ=    25.1094   YZ=     2.0157   ZZ=    46.8280
   Eigenvalues:   -30.0732    50.0913   105.9137
133  C    Isotropic =    42.4112   Anisotropy =    95.9156
   XX=    -3.8349   YX=    14.3085   ZX=   -46.6164
   XY=    11.2872   YY=    59.1194   ZY=   -15.0060
   XZ=   -54.1659   YZ=   -18.8858   ZZ=    71.9490
   Eigenvalues:   -29.1687    50.0473   106.3549
134  C    Isotropic =    62.0884   Anisotropy =   152.8635
   XX=    49.9487   YX=     5.2869   ZX=   -56.2684
   XY=    20.1219   YY=    25.5134   ZY=   -48.1468
   XZ=   -54.6308   YZ=   -59.4332   ZZ=   110.8033
   Eigenvalues:    -5.2811    27.5490   163.9975
135  C    Isotropic =    64.8843   Anisotropy =   126.2637
   XX=    51.0707   YX=    52.4301   ZX=   -28.4131
   XY=    48.9370   YY=    22.1148   ZY=   -29.2462
   XZ=   -26.3946   YZ=   -32.0143   ZZ=   121.4674
   Eigenvalues:   -16.6330    62.2258   149.0601
136  C    Isotropic =    61.2066   Anisotropy =   151.1178
   XX=    26.1234   YX=    26.6085   ZX=   -65.6610
   XY=    11.5067   YY=    43.5547   ZY=   -43.2479
   XZ=   -60.5987   YZ=   -31.9976   ZZ=   113.9417
   Eigenvalues:    -6.8734    28.5414   161.9518
137  C    Isotropic =    42.0449   Anisotropy =    96.6865
   XX=    50.8782   YX=   -12.9550   ZX=   -31.5813
   XY=   -12.4429   YY=     2.7011   ZY=   -48.7298
   XZ=   -27.3994   YZ=   -50.7066   ZZ=    72.5552
   Eigenvalues:   -30.9905    50.6225   106.5025
138  C    Isotropic =    74.8693   Anisotropy =   131.0618
   XX=    53.8060   YX=    40.7375   ZX=   -34.0506
   XY=    37.4727   YY=    43.0099   ZY=   -35.5040
   XZ=   -30.7943   YZ=   -43.1472   ZZ=   127.7918
   Eigenvalues:     8.1399    54.2241   162.2438
139  C    Isotropic =   156.1850   Anisotropy =    12.9896
   XX=   164.6248   YX=     0.8000   ZX=     0.0666
   XY=     0.1584   YY=   161.3894   ZY=    -9.6662
   XZ=    -2.5607   YZ=     2.9759   ZZ=   142.5409
   Eigenvalues:   141.9068   161.8035   164.8448
140  C    Isotropic =   155.9048   Anisotropy =    12.8845
   XX=   145.2979   YX=   -12.4896   ZX=     6.7090
   XY=    -2.7319   YY=   158.8412   ZY=     2.1979
```

```
   XZ=     0.8780   YZ=     2.1474   ZZ=   163.5752
   Eigenvalues:   141.0421   162.1777   164.4944
141  C    Isotropic =   156.2676   Anisotropy =    12.4244
   XX=   164.4700   YX=     0.6043   ZX=    -0.9414
   XY=     1.1054   YY=   154.5346   ZY=    -4.3563
   XZ=     2.1769   YZ=   -14.9003   ZZ=   149.7983
   Eigenvalues:   142.2050   162.0473   164.5506
142  C    Isotropic =   155.8452   Anisotropy =    13.1005
   XX=   140.9147   YX=     4.8547   ZX=    -2.2378
   XY=    -5.0863   YY=   162.5458   ZY=     1.2577
   XZ=     2.2352   YZ=     0.7659   ZZ=   164.0752
   Eigenvalues:   140.9141   162.0427   164.5789
143  O    Isotropic =   189.1599   Anisotropy =   143.5725
   XX=   169.2079   YX=   -35.4809   ZX=   -39.2182
   XY=   -56.9575   YY=   170.9229   ZY=    56.2614
   XZ=   -15.6953   YZ=    61.3730   ZZ=   227.3490
   Eigenvalues:   117.3636   165.2412   284.8749
144  O    Isotropic =   186.3435   Anisotropy =   143.2516
   XX=   253.1358   YX=   -14.0542   ZX=   -53.7303
   XY=    -7.6156   YY=   167.0744   ZY=    26.5666
   XZ=   -71.2518   YZ=   -18.0725   ZZ=   138.8204
   Eigenvalues:   111.2854   165.9006   281.8446
145  O    Isotropic =   190.5531   Anisotropy =   120.1996
   XX=   263.8493   YX=    11.3873   ZX=    28.8560
   XY=     6.6066   YY=   128.8297   ZY=   -34.1792
   XZ=    20.9583   YZ=    -8.9376   ZZ=   178.9804
   Eigenvalues:   118.7358   182.2374   270.6862
146  O    Isotropic =   190.5245   Anisotropy =   121.7121
   XX=   168.1939   YX=    -9.9858   ZX=    30.9856
   XY=   -34.1718   YY=   149.9301   ZY=    30.8938
   XZ=    44.0851   YZ=    31.5287   ZZ=   253.4494
   Eigenvalues:   118.5590   181.3485   271.6659
147  O    Isotropic =   189.8282   Anisotropy =   139.6033
   XX=   135.3647   YX=    26.9296   ZX=    20.8176
   XY=    47.4869   YY=   215.2204   ZY=    59.2230
   XZ=    -1.3144   YZ=    57.3986   ZZ=   218.8995
   Eigenvalues:   118.5668   168.0207   282.8971
148  O    Isotropic =   190.2185   Anisotropy =   139.8592
   XX=   243.9510   YX=    51.9908   ZX=    -7.4301
   XY=    59.0052   YY=   203.1832   ZY=    29.6116
   XZ=    10.0243   YZ=     5.2050   ZZ=   123.5211
   Eigenvalues:   118.7699   168.4275   283.4579
149  O    Isotropic =   189.3976   Anisotropy =   143.3257
   XX=   244.6135   YX=    36.9092   ZX=   -39.6073
   XY=    26.9539   YY=   134.2250   ZY=   -45.5419
   XZ=   -55.6695   YZ=   -19.5097   ZZ=   189.3543
   Eigenvalues:   118.2568   164.9880   284.9481
150  O    Isotropic =   186.9439   Anisotropy =   142.7615
   XX=   135.0586   YX=   -25.4617   ZX=   -59.9053
   XY=    21.1248   YY=   170.5568   ZY=    26.0618
   XZ=   -54.2196   YZ=    18.3390   ZZ=   255.2163
   Eigenvalues:   111.5909   167.1225   282.1182
151  C    Isotropic =    49.4963   Anisotropy =   135.8777
   XX=   130.0187   YX=    35.9364   ZX=   -17.5532
```

```
   XY=    42.0556   YY=   -35.3038   ZY=    26.7168
   XZ=   -11.6494   YZ=     6.9488   ZZ=    53.7741
   Eigenvalues:   -47.8499    56.2575   140.0815
152  C    Isotropic =    49.9599   Anisotropy =   134.4942
   XX=   129.5226   YX=    34.6484   ZX=   -18.8454
   XY=    34.6544   YY=     4.4874   ZY=    37.1924
   XZ=   -26.5754   YZ=    49.8925   ZZ=    15.8697
   Eigenvalues:   -43.4813    53.7383   139.6227
153  N    Isotropic =   -54.1085   Anisotropy =   474.2049
   XX=   221.2904   YX=   145.4126   ZX=   -66.7309
   XY=   131.0232   YY=  -283.3881   ZY=    62.2577
   XZ=   -52.2625   YZ=    51.2714   ZZ=  -100.2278
   Eigenvalues:  -339.3769   -84.9768   262.0281
154  C    Isotropic =    38.3722   Anisotropy =    95.9357
   XX=    94.3661   YX=    33.7994   ZX=     0.4925
   XY=    23.4157   YY=   -15.4356   ZY=   -37.4187
   XZ=    -5.0738   YZ=    -8.7911   ZZ=    36.1861
   Eigenvalues:   -29.7627    42.5500   102.3293
155  C    Isotropic =    37.7549   Anisotropy =    97.1625
   XX=    93.9299   YX=    20.7595   ZX=   -28.6303
   XY=    19.0263   YY=    37.6209   ZY=    34.3929
   XZ=   -17.3549   YZ=     5.0056   ZZ=   -18.2861
   Eigenvalues:   -30.8778    41.6126   102.5299
156  N    Isotropic =   -56.9030   Anisotropy =   477.8121
   XX=   220.3835   YX=   143.0703   ZX=   -79.2812
   XY=   120.3592   YY=  -236.0163   ZY=    97.1710
   XZ=   -75.1418   YZ=   106.0621   ZZ=  -155.0761
   Eigenvalues:  -345.9369   -86.4104   261.6385
157  N    Isotropic =   -54.2843   Anisotropy =   474.7054
   XX=   -86.8211   YX=    13.7771   ZX=   -14.1148
   XY=    21.6862   YY=  -220.0599   ZY=   229.7308
   XZ=   -26.6067   YZ=   246.9027   ZZ=   144.0283
   Eigenvalues:  -340.3584   -84.6804   262.1860
158  C    Isotropic =    49.7370   Anisotropy =   134.3832
   XX=    31.5388   YX=    41.7856   ZX=   -23.8498
   XY=    31.1213   YY=     7.8040   ZY=    63.3812
   XZ=   -14.4898   YZ=    61.0263   ZZ=   109.8683
   Eigenvalues:   -44.0338    53.9190   139.3258
159  C    Isotropic =    49.6838   Anisotropy =   135.7745
   XX=    56.8791   YX=   -10.3440   ZX=     2.0564
   XY=     7.2157   YY=   -15.4289   ZY=    72.7859
   XZ=    -8.0941   YZ=    69.3030   ZZ=   107.6012
   Eigenvalues:   -47.8897    56.7409   140.2001
160  N    Isotropic =   -56.6271   Anisotropy =   477.1083
   XX=  -121.0834   YX=    78.8639   ZX=   -52.9712
   XY=    69.7744   YY=  -197.1703   ZY=   215.4305
   XZ=   -49.8078   YZ=   238.8545   ZZ=   148.3724
   Eigenvalues:  -345.4568   -85.8696   261.4451
161  C    Isotropic =    37.9717   Anisotropy =    96.9328
   XX=    -9.0469   YX=    13.6104   ZX=   -11.3307
   XY=    39.7767   YY=    39.3534   ZY=    31.6321
   XZ=   -27.6563   YZ=    37.2182   ZZ=    83.6087
   Eigenvalues:   -30.3023    41.6239   102.5936
162  C    Isotropic =    38.2140   Anisotropy =    96.3491
```

```
   XX=    24.4883   YX=   -15.2478   ZX=     5.6199
   XY=   -41.5249   YY=    11.8444   ZY=    43.1460
   XZ=    18.4823   YZ=    50.1972   ZZ=    78.3092
   Eigenvalues:   -29.6873    41.8825   102.4467
163  N    Isotropic =   -45.8561   Anisotropy =   470.3946
   XX=   188.5192   YX=   112.5208   ZX=  -107.1507
   XY=   161.6462   YY=  -279.9030   ZY=    25.6847
   XZ=  -140.6376   YZ=    16.8021   ZZ=   -46.1845
   Eigenvalues:  -327.9173   -77.3912   267.7403
164  C    Isotropic =    39.2760   Anisotropy =    97.9365
   XX=    98.5393   YX=     3.3621   ZX=    -8.0779
   XY=    19.0081   YY=   -17.8884   ZY=   -45.1365
   XZ=   -22.3614   YZ=    -6.9228   ZZ=    37.1772
   Eigenvalues:   -28.4302    41.6913   104.5671
165  C    Isotropic =    38.4799   Anisotropy =    96.5537
   XX=    91.4581   YX=    14.5573   ZX=   -36.4158
   XY=    20.1193   YY=    35.5620   ZY=    34.6780
   XZ=   -28.7407   YZ=     5.5307   ZZ=   -11.5804
   Eigenvalues:   -30.0258    42.6164   102.8490
166  N    Isotropic =   -57.2289   Anisotropy =   477.2527
   XX=   192.1431   YX=   144.6305   ZX=  -128.7980
   XY=   118.3042   YY=  -249.9276   ZY=    82.5786
   XZ=  -138.2853   YZ=    81.2808   ZZ=  -113.9021
   Eigenvalues:  -347.3537   -85.2724   260.9396
167  C    Isotropic =    50.6868   Anisotropy =   134.4348
   XX=   119.0413   YX=    44.4966   ZX=   -31.8922
   XY=    38.9565   YY=   -32.8844   ZY=    18.5098
   XZ=   -28.7729   YZ=    -6.1643   ZZ=    65.9035
   Eigenvalues:   -45.3015    57.0519   140.3100
168  C    Isotropic =    50.4828   Anisotropy =   134.2493
   XX=   106.6563   YX=    51.2833   ZX=   -45.5082
   XY=    44.4466   YY=     5.0810   ZY=    24.7356
   XZ=   -54.3341   YZ=    36.5334   ZZ=    39.7110
   Eigenvalues:   -43.0566    54.5226   139.9824
169  N    Isotropic =   -46.9632   Anisotropy =   470.8809
   XX=   -59.5575   YX=   -42.6190   ZX=   -78.8726
   XY=   -26.5112   YY=  -183.3171   ZY=   277.1619
   XZ=   -62.0720   YZ=   225.1645   ZZ=   101.9852
   Eigenvalues:  -329.6324   -78.2145   266.9575
170  C    Isotropic =    39.2218   Anisotropy =    98.0219
   XX=    22.8684   YX=   -18.4196   ZX=    -9.2177
   XY=   -51.5547   YY=     7.8610   ZY=    47.7261
   XZ=     9.8042   YZ=    28.4475   ZZ=    86.9360
   Eigenvalues:   -28.6063    41.7020   104.5697
171  C    Isotropic =    38.4740   Anisotropy =    96.3127
   XX=    -4.2472   YX=    10.0579   ZX=   -21.2847
   XY=    37.1159   YY=    38.6381   ZY=    36.9489
   XZ=   -35.6473   YZ=    33.1629   ZZ=    81.0310
   Eigenvalues:   -29.9846    42.7241   102.6824
172  N    Isotropic =   -56.8696   Anisotropy =   476.6778
   XX=   -98.1861   YX=    35.2930   ZX=  -100.6784
   XY=    36.5023   YY=  -181.3283   ZY=   236.0192
   XZ=   -86.8375   YZ=   255.7672   ZZ=   108.9056
   Eigenvalues:  -346.6870   -84.8374   260.9156
```

```
173  C    Isotropic =    50.6126   Anisotropy =   134.6876
   XX=    61.3872   YX=   -26.6845   ZX=    -9.7380
   XY=    -4.3033   YY=    -0.6363   ZY=    73.7581
   XZ=   -18.5893   YZ=    81.3803   ZZ=    91.0871
   Eigenvalues:   -45.2842    56.7178   140.4043
174  C    Isotropic =    50.3152   Anisotropy =   134.6666
   XX=    45.4338   YX=    20.9090   ZX=   -45.1435
   XY=    11.4135   YY=    25.0821   ZY=    74.1578
   XZ=   -32.3794   YZ=    79.3007   ZZ=    80.4296
   Eigenvalues:   -43.4581    54.3107   140.0929
175  C    Isotropic =    34.8090   Anisotropy =    81.8639
   XX=    82.3550   YX=    22.2495   ZX=   -18.7748
   XY=    21.3901   YY=    15.6848   ZY=    41.7531
   XZ=   -21.2986   YZ=    57.7511   ZZ=     6.3873
   Eigenvalues:   -45.7332    60.7753    89.3850
176  C    Isotropic =    30.6091   Anisotropy =    97.5870
   XX=    88.4296   YX=    28.5795   ZX=    -9.7435
   XY=    28.6505   YY=   -33.2185   ZY=    14.9197
   XZ=    -9.1913   YZ=     5.7197   ZZ=    36.6162
   Eigenvalues:   -41.5073    37.6675    95.6671
177  C    Isotropic =    30.7094   Anisotropy =    97.5748
   XX=    37.7890   YX=    -8.5724   ZX=     0.6357
   XY=     0.7033   YY=   -17.9513   ZY=    51.4323
   XZ=    -2.0441   YZ=    51.6498   ZZ=    72.2905
   Eigenvalues:   -41.4681    37.8370    95.7592
178  C    Isotropic =    34.3308   Anisotropy =    83.0802
   XX=    24.6116   YX=    51.6321   ZX=   -24.8880
   XY=    36.5895   YY=     7.4651   ZY=    39.3202
   XZ=   -19.0634   YZ=    39.2905   ZZ=    70.9157
   Eigenvalues:   -45.8047    59.0795    89.7176
179  C    Isotropic =    35.7387   Anisotropy =    82.1765
   XX=    65.4070   YX=    36.4544   ZX=   -38.4689
   XY=    29.4538   YY=    15.1250   ZY=    35.6173
   XZ=   -40.0405   YZ=    49.5085   ZZ=    26.6841
   Eigenvalues:   -45.3290    62.0221    90.5231
180  C    Isotropic =    30.8939   Anisotropy =    95.2522
   XX=    75.2799   YX=    25.2284   ZX=   -24.7125
   XY=    31.9058   YY=   -33.2072   ZY=     5.7520
   XZ=   -23.5608   YZ=    -0.8442   ZZ=    50.6090
   Eigenvalues:   -41.0344    39.3206    94.3953
181  C    Isotropic =    35.3905   Anisotropy =    82.9317
   XX=    39.0942   YX=    36.9286   ZX=   -40.1521
   XY=    24.0568   YY=    17.6503   ZY=    48.0862
   XZ=   -33.8667   YZ=    52.5354   ZZ=    49.4271
   Eigenvalues:   -45.1764    60.6697    90.6783
182  C    Isotropic =    31.0393   Anisotropy =    94.9890
   XX=    45.8473   YX=   -19.2013   ZX=    -9.7311
   XY=   -12.5505   YY=    -9.5235   ZY=    57.8941
   XZ=   -13.3342   YZ=    51.8190   ZZ=    56.7941
   Eigenvalues:   -41.2514    40.0041    94.3653
183  O    Isotropic =  -142.9333   Anisotropy =   770.6682
   XX=   236.3814   YX=    22.9916   ZX=  -270.9574
   XY=    26.0514   YY=  -385.6826   ZY=  -214.6665
   XZ=  -275.6706   YZ=  -219.6737   ZZ=  -279.4987
```

```
   Eigenvalues:  -587.4839  -212.1615   370.8455
184  O    Isotropic =   -83.4660   Anisotropy =   675.9819
   XX=   179.9668   YX=    68.0941   ZX=  -328.7595
   XY=    69.1624   YY=  -139.8068   ZY=   -96.1978
   XZ=  -322.5341   YZ=   -84.6135   ZZ=  -290.5580
   Eigenvalues:  -464.8086  -152.7779   367.1886
185  O    Isotropic =  -134.1912   Anisotropy =   756.2705
   XX=  -455.3811   YX=  -163.0514   ZX=    52.0152
   XY=  -158.6548   YY=  -199.9095   ZY=   257.7067
   XZ=    52.8196   YZ=   258.1599   ZZ=   252.7171
   Eigenvalues:  -568.1947  -204.3679   369.9891
186  N    Isotropic =    88.7035   Anisotropy =   103.6433
   XX=    62.8231   YX=     9.5283   ZX=    -6.9242
   XY=     6.2455   YY=    63.8309   ZY=    40.1349
   XZ=    -6.8779   YZ=    42.6153   ZZ=   139.4565
   Eigenvalues:    40.9937    67.3178   157.7991
187  O    Isotropic =   -82.6022   Anisotropy =   671.7886
   XX=  -457.2549   YX=    10.9952   ZX=   -37.5588
   XY=     0.8112   YY=   -63.6325   ZY=   197.1655
   XZ=   -35.3345   YZ=   197.4990   ZZ=   273.0807
   Eigenvalues:  -459.7876  -153.2759   365.2568
188  O    Isotropic =  -137.9553   Anisotropy =   761.3251
   XX=   334.8147   YX=   138.2357   ZX=   -41.5685
   XY=   137.8340   YY=  -363.8817   ZY=  -193.9845
   XZ=   -41.9583   YZ=  -197.6683   ZZ=  -384.7991
   Eigenvalues:  -575.2611  -208.1996   369.5947
189  O    Isotropic =   -80.9428   Anisotropy =   669.3700
   XX=   332.7108   YX=   100.7254   ZX=   -88.7750
   XY=    99.9564   YY=  -135.8123   ZY=   -62.3946
   XZ=   -88.0490   YZ=   -52.4372   ZZ=  -439.7269
   Eigenvalues:  -456.5537  -151.5786   365.3039
190  O    Isotropic =  -139.9314   Anisotropy =   767.3270
   XX=  -411.7741   YX=  -256.2667   ZX=  -152.2171
   XY=  -250.6581   YY=  -206.8955   ZY=   220.4670
   XZ=  -149.3407   YZ=   216.2330   ZZ=   198.8755
   Eigenvalues:  -582.7285  -208.6855   371.6200
191  O    Isotropic =   -86.6867   Anisotropy =   681.3161
   XX=  -369.8426   YX=   -93.6219   ZX=  -246.7676
   XY=  -107.0130   YY=   -31.9111   ZY=   200.9121
   XZ=  -250.5372   YZ=   199.5088   ZZ=   141.6936
   Eigenvalues:  -471.6302  -155.9540   367.5240
192  H    Isotropic =    24.9405   Anisotropy =     8.2717
   XX=    25.9405   YX=     2.5024   ZX=     3.3007
   XY=     3.6020   YY=    23.4785   ZY=     1.4103
   XZ=     2.6299   YZ=     2.3912   ZZ=    25.4026
   Eigenvalues:    21.4151    22.9515    30.4550
193  H    Isotropic =    23.9207   Anisotropy =     7.4630
   XX=    23.1704   YX=     2.6479   ZX=     3.3961
   XY=     3.3065   YY=    23.8608   ZY=     0.2563
   XZ=     4.6776   YZ=    -0.1652   ZZ=    24.7307
   Eigenvalues:    18.7483    24.1178    28.8960
194  H    Isotropic =    24.9287   Anisotropy =     8.9545
   XX=    26.2487   YX=    -0.7938   ZX=    -4.4615
   XY=     0.4574   YY=    22.4715   ZY=     2.3506
```

```
   XZ=    -4.6009   YZ=     1.0680   ZZ=    26.0659
   Eigenvalues:    20.8077    23.0800    30.8984
195  H    Isotropic =    24.3773   Anisotropy =     7.4822
   XX=    24.4089   YX=    -0.0767   ZX=    -5.0016
   XY=    -1.1280   YY=    24.1741   ZY=     0.0595
   XZ=    -4.5109   YZ=     1.0580   ZZ=    24.5489
   Eigenvalues:    19.7218    24.0446    29.3654
196  H    Isotropic =    23.9456   Anisotropy =     7.6642
   XX=    25.0039   YX=    -2.9967   ZX=    -3.2276
   XY=    -2.4997   YY=    20.7826   ZY=    -0.6607
   XZ=    -3.4669   YZ=    -1.4049   ZZ=    26.0502
   Eigenvalues:    18.5588    24.2229    29.0550
197  H    Isotropic =    24.8728   Anisotropy =     8.7524
   XX=    26.5136   YX=    -0.5927   ZX=    -3.9909
   XY=    -1.6654   YY=    21.3455   ZY=     0.4184
   XZ=    -3.6939   YZ=     1.4526   ZZ=    26.7593
   Eigenvalues:    21.1038    22.8068    30.7078
198  H    Isotropic =    24.9377   Anisotropy =     8.3789
   XX=    26.4488   YX=     2.5823   ZX=     2.8453
   XY=     1.7720   YY=    23.8367   ZY=     3.4384
   XZ=     3.4833   YZ=     2.0230   ZZ=    24.5278
   Eigenvalues:    21.3455    22.9440    30.5237
199  H    Isotropic =    23.8875   Anisotropy =     7.4513
   XX=    25.0930   YX=     0.8854   ZX=     5.0903
   XY=     0.9362   YY=    24.7979   ZY=     1.8314
   XZ=     3.6455   YZ=     1.6104   ZZ=    21.7716
   Eigenvalues:    18.6213    24.1862    28.8550
200  H    Isotropic =    26.9797   Anisotropy =     3.5669
   XX=    24.6201   YX=    -0.7685   ZX=     0.2786
   XY=    -0.5022   YY=    27.2039   ZY=     1.1571
   XZ=     0.6166   YZ=     0.2500   ZZ=    29.1152
   Eigenvalues:    24.3954    27.1862    29.3577
201  H    Isotropic =    28.9167   Anisotropy =     6.6700
   XX=    27.5069   YX=    -1.1548   ZX=     1.0428
   XY=    -1.5432   YY=    31.8802   ZY=    -4.1255
   XZ=     0.2928   YZ=    -0.8378   ZZ=    27.3630
   Eigenvalues:    26.2613    27.1254    33.3633
202  H    Isotropic =    28.9018   Anisotropy =     6.8614
   XX=    26.5692   YX=    -2.9725   ZX=     1.5245
   XY=     0.1454   YY=    32.2830   ZY=    -1.9176
   XZ=     0.1455   YZ=    -2.2087   ZZ=    27.8534
   Eigenvalues:    26.1195    27.1099    33.4761
203  H    Isotropic =    27.1016   Anisotropy =     3.5995
   XX=    29.2651   YX=     1.3612   ZX=    -0.0595
   XY=     0.0823   YY=    27.0888   ZY=    -0.7349
   XZ=    -0.1096   YZ=    -0.6771   ZZ=    24.9508
   Eigenvalues:    24.7350    27.0685    29.5012
204  H    Isotropic =    28.9237   Anisotropy =     6.8514
   XX=    27.3882   YX=    -1.1932   ZX=     0.3643
   XY=    -0.6363   YY=    32.0120   ZY=    -1.0665
   XZ=     1.0587   YZ=    -4.4136   ZZ=    27.3709
   Eigenvalues:    26.0426    27.2371    33.4913
205  H    Isotropic =    27.1287   Anisotropy =     3.6014
   XX=    24.8562   YX=    -0.2153   ZX=     0.1668
```

```
   XY=    -0.1643   YY=    27.6712   ZY=     0.5433
   XZ=     0.4816   YZ=     1.6741   ZZ=    28.8586
   Eigenvalues:    24.8001    27.0563    29.5296
206  H    Isotropic =    27.0176   Anisotropy =     3.3986
   XX=    29.2635   YX=    -0.1920   ZX=     0.1479
   XY=     0.5947   YY=    26.6685   ZY=    -0.9882
   XZ=    -0.2419   YZ=    -1.1142   ZZ=    25.1208
   Eigenvalues:    24.5888    27.1806    29.2834
207  H    Isotropic =    28.9315   Anisotropy =     6.7024
   XX=    26.6142   YX=     0.1378   ZX=     0.0103
   XY=    -2.9201   YY=    31.9227   ZY=    -2.3493
   XZ=     1.4145   YZ=    -2.2736   ZZ=    28.2575
   Eigenvalues:    26.2530    27.1417    33.3997
208  H    Isotropic =    21.5550   Anisotropy =    16.7545
   XX=    21.9560   YX=    -4.8075   ZX=     1.0473
   XY=    -4.3130   YY=    29.5998   ZY=    -4.5082
   XZ=     1.1980   YZ=    -4.1900   ZZ=    13.1092
   Eigenvalues:    12.0325    19.9079    32.7247
209  N    Isotropic =    88.5141   Anisotropy =   104.0504
   XX=   152.0435   YX=    22.6097   ZX=   -13.9547
   XY=    20.4442   YY=    54.8132   ZY=     8.3337
   XZ=   -13.1625   YZ=    11.5796   ZZ=    58.6856
   Eigenvalues:    40.8581    66.8033   157.8810
210  H    Isotropic =    21.5288   Anisotropy =    16.6425
   XX=    12.0988   YX=    -0.8264   ZX=     0.9825
   XY=    -1.0578   YY=    28.4099   ZY=    -5.7072
   XZ=     0.6670   YZ=    -6.1342   ZZ=    24.0777
   Eigenvalues:    12.0210    19.9416    32.6238
211  N    Isotropic =    88.0061   Anisotropy =   106.8567
   XX=   134.5168   YX=    22.1129   ZX=   -44.1321
   XY=    19.6468   YY=    54.7520   ZY=     0.0337
   XZ=   -39.8988   YZ=     3.4507   ZZ=    74.7496
   Eigenvalues:    41.4055    63.3689   159.2439
212  H    Isotropic =    21.4802   Anisotropy =    18.9652
   XX=    13.4599   YX=    -5.3926   ZX=     5.0021
   XY=    -4.3161   YY=    28.9132   ZY=    -5.3092
   XZ=     4.5497   YZ=    -5.8001   ZZ=    22.0674
   Eigenvalues:    10.9997    19.3171    34.1236
213  N    Isotropic =    87.3043   Anisotropy =   109.1620
   XX=    67.5882   YX=    -7.3373   ZX=   -29.1407
   XY=   -10.3546   YY=    72.0343   ZY=    44.0811
   XZ=   -32.1195   YZ=    48.2554   ZZ=   122.2904
   Eigenvalues:    40.9767    60.8573   160.0789
214  H    Isotropic =    21.2049   Anisotropy =    19.5911
   XX=    20.2427   YX=    -3.8737   ZX=     3.9333
   XY=    -3.2427   YY=    28.0988   ZY=    -8.1065
   XZ=     3.9753   YZ=    -9.2884   ZZ=    15.2732
   Eigenvalues:    10.4527    18.8964    34.2656
215  C    Isotropic =    64.8843   Anisotropy =   126.2637
   XX=    51.0707   YX=    52.4301   ZX=    28.4131
   XY=    48.9370   YY=    22.1148   ZY=    29.2462
   XZ=    26.3946   YZ=    32.0143   ZZ=   121.4674
   Eigenvalues:   -16.6330    62.2258   149.0601
216  C    Isotropic =    61.2066   Anisotropy =   151.1178
```

```
   XX=    26.1234   YX=    26.6085   ZX=    65.6610
   XY=    11.5067   YY=    43.5547   ZY=    43.2479
   XZ=    60.5987   YZ=    31.9976   ZZ=   113.9417
   Eigenvalues:    -6.8734    28.5414   161.9518
217  C    Isotropic =    42.0449   Anisotropy =    96.6865
   XX=    50.8782   YX=   -12.9550   ZX=    31.5813
   XY=   -12.4429   YY=     2.7011   ZY=    48.7298
   XZ=    27.3994   YZ=    50.7066   ZZ=    72.5552
   Eigenvalues:   -30.9905    50.6225   106.5025
218  C    Isotropic =    74.8693   Anisotropy =   131.0618
   XX=    53.8060   YX=    40.7375   ZX=    34.0506
   XY=    37.4727   YY=    43.0099   ZY=    35.5040
   XZ=    30.7943   YZ=    43.1472   ZZ=   127.7918
   Eigenvalues:     8.1399    54.2241   162.2438
219  C    Isotropic =    42.4112   Anisotropy =    95.9156
   XX=    -3.8349   YX=    14.3085   ZX=    46.6164
   XY=    11.2872   YY=    59.1194   ZY=    15.0060
   XZ=    54.1659   YZ=    18.8858   ZZ=    71.9490
   Eigenvalues:   -29.1687    50.0473   106.3549
220  C    Isotropic =    62.0884   Anisotropy =   152.8635
   XX=    49.9487   YX=     5.2869   ZX=    56.2684
   XY=    20.1219   YY=    25.5134   ZY=    48.1468
   XZ=    54.6308   YZ=    59.4332   ZZ=   110.8033
   Eigenvalues:    -5.2811    27.5490   163.9975
221  C    Isotropic =    61.8068   Anisotropy =   152.5349
   XX=    25.2521   YX=    64.0283   ZX=   -15.5701
   XY=    54.3293   YY=   129.2041   ZY=   -30.6786
   XZ=   -28.2533   YZ=   -18.5722   ZZ=    30.9642
   Eigenvalues:    -4.7855    26.7091   163.4967
222  C    Isotropic =    63.7200   Anisotropy =   129.2133
   XX=    45.8627   YX=    54.0662   ZX=    24.3764
   XY=    50.6533   YY=   117.3625   ZY=   -37.2095
   XZ=    24.0255   YZ=   -40.5965   ZZ=    27.9347
   Eigenvalues:   -18.2588    59.5565   149.8622
223  C    Isotropic =    61.5844   Anisotropy =   153.2047
   XX=    49.0443   YX=    46.4350   ZX=   -29.1247
   XY=    55.4389   YY=   131.6895   ZY=   -21.3257
   XZ=   -16.9610   YZ=   -34.5884   ZZ=     4.0194
   Eigenvalues:    -6.1000    27.1324   163.7209
224  C    Isotropic =    41.9773   Anisotropy =    95.9046
   XX=    -4.7467   YX=    46.5793   ZX=   -18.3244
   XY=    47.6394   YY=    83.8504   ZY=    -0.4796
   XZ=   -25.1094   YZ=    -2.0157   ZZ=    46.8280
   Eigenvalues:   -30.0732    50.0913   105.9137
225  C    Isotropic =    75.9951   Anisotropy =   130.3573
   XX=    54.1189   YX=    51.6115   ZX=    13.0892
   XY=    45.2759   YY=   136.3979   ZY=   -26.9425
   XZ=    11.1727   YZ=   -33.3205   ZZ=    37.4686
   Eigenvalues:     9.2362    55.8492   162.9000
226  C    Isotropic =    42.2216   Anisotropy =    94.9645
   XX=    51.5254   YX=    17.7379   ZX=   -27.7442
   XY=    14.5945   YY=    94.4264   ZY=   -19.2716
   XZ=   -22.4131   YZ=   -18.9717   ZZ=   -19.2871
   Eigenvalues:   -28.7710    49.9044   105.5312
```

```
227  C    Isotropic =    74.2387   Anisotropy =   131.2398
   XX=    97.2202   YX=   -29.8477   ZX=   -49.7282
   XY=   -30.2579   YY=    17.5007   ZY=    21.7318
   XZ=   -49.6667   YZ=    21.6819   ZZ=   107.9953
   Eigenvalues:     7.1185    53.8657   161.7319
228  C    Isotropic =    41.2940   Anisotropy =    96.7713
   XX=    54.3125   YX=    15.2680   ZX=   -50.4049
   XY=    11.6291   YY=    18.3247   ZY=    38.5317
   XZ=   -45.5382   YZ=    44.1160   ZZ=    51.2449
   Eigenvalues:   -29.2551    47.3290   105.8082
229  C    Isotropic =    62.5296   Anisotropy =   153.7000
   XX=    66.5899   YX=   -34.8934   ZX=   -82.9051
   XY=   -18.3036   YY=    31.3006   ZY=    23.4596
   XZ=   -76.3261   YZ=     7.7179   ZZ=    89.6983
   Eigenvalues:    -5.4223    28.0147   164.9963
230  C    Isotropic =    62.5010   Anisotropy =   131.0571
   XX=    91.6746   YX=   -38.8559   ZX=   -41.5316
   XY=   -35.5432   YY=    -8.1997   ZY=    20.1043
   XZ=   -40.3266   YZ=    23.3020   ZZ=   104.0282
   Eigenvalues:   -21.0822    58.7127   149.8724
231  C    Isotropic =    62.8259   Anisotropy =   153.4143
   XX=    86.5027   YX=    -4.8419   ZX=   -72.8384
   XY=   -19.5699   YY=    24.5453   ZY=    25.4320
   XZ=   -78.1088   YZ=    42.9465   ZZ=    77.4297
   Eigenvalues:    -5.2964    28.6720   165.1021
232  C    Isotropic =    41.3883   Anisotropy =    96.3946
   XX=    35.3885   YX=   -35.1490   ZX=   -56.0293
   XY=   -29.1971   YY=    40.2031   ZY=    -5.1106
   XZ=   -61.3219   YZ=    -8.1401   ZZ=    48.5732
   Eigenvalues:   -29.0712    47.5846   105.6513
233  C    Isotropic =    42.0999   Anisotropy =    97.0281
   XX=    45.4977   YX=   -65.6673   ZX=    16.0832
   XY=   -64.8797   YY=    26.5999   ZY=    -2.4957
   XZ=    20.7808   YZ=    -3.0435   ZZ=    54.2022
   Eigenvalues:   -31.0963    50.6108   106.7854
234  C    Isotropic =    61.1818   Anisotropy =   151.7790
   XX=    89.5552   YX=   -52.7894   ZX=    50.5405
   XY=   -64.2630   YY=    74.6471   ZY=   -18.4590
   XZ=    50.0016   YZ=   -33.4828   ZZ=    19.3432
   Eigenvalues:    -7.2001    28.3778   162.3678
235  C    Isotropic =    65.1561   Anisotropy =   126.0875
   XX=   102.7067   YX=   -49.3099   ZX=     8.6504
   XY=   -47.0411   YY=    62.8914   ZY=   -53.6315
   XZ=    10.0234   YZ=   -57.8415   ZZ=    29.8702
   Eigenvalues:   -16.1847    62.4386   149.2144
236  C    Isotropic =    62.0648   Anisotropy =   152.6754
   XX=    79.7222   YX=   -79.5710   ZX=    35.3881
   XY=   -69.0788   YY=    62.4805   ZY=   -32.8698
   XZ=    42.2197   YZ=   -19.2316   ZZ=    43.9917
   Eigenvalues:    -4.8875    27.2335   163.8485
237  C    Isotropic =    42.5346   Anisotropy =    95.2229
   XX=    59.5912   YX=   -29.0395   ZX=    47.9885
   XY=   -23.7985   YY=    73.5624   ZY=    -8.3674
   XZ=    41.6524   YZ=    -9.6054   ZZ=    -5.5500
```

```
   Eigenvalues:   -28.5511    50.1383   106.0165
238  C    Isotropic =    74.9658   Anisotropy =   130.9378
   XX=   104.9210   YX=   -58.5779   ZX=    13.2413
   XY=   -51.6960   YY=    81.9948   ZY=   -40.1984
   XZ=    17.0965   YZ=   -45.1026   ZZ=    37.9815
   Eigenvalues:     8.2630    54.3766   162.2577
239  C    Isotropic =   156.2676   Anisotropy =    12.4244
   XX=   164.4700   YX=     0.6043   ZX=     0.9414
   XY=     1.1054   YY=   154.5346   ZY=     4.3563
   XZ=    -2.1769   YZ=    14.9003   ZZ=   149.7983
   Eigenvalues:   142.2050   162.0473   164.5506
240  C    Isotropic =   155.8452   Anisotropy =    13.1005
   XX=   140.9147   YX=     4.8547   ZX=     2.2378
   XY=    -5.0863   YY=   162.5458   ZY=    -1.2577
   XZ=    -2.2352   YZ=    -0.7659   ZZ=   164.0752
   Eigenvalues:   140.9141   162.0427   164.5789
241  C    Isotropic =   156.1850   Anisotropy =    12.9896
   XX=   164.6248   YX=     0.8000   ZX=    -0.0666
   XY=     0.1584   YY=   161.3894   ZY=     9.6662
   XZ=     2.5607   YZ=    -2.9759   ZZ=   142.5409
   Eigenvalues:   141.9068   161.8035   164.8448
242  C    Isotropic =   155.9048   Anisotropy =    12.8845
   XX=   145.2979   YX=   -12.4896   ZX=    -6.7090
   XY=    -2.7319   YY=   158.8412   ZY=    -2.1979
   XZ=    -0.8780   YZ=    -2.1474   ZZ=   163.5752
   Eigenvalues:   141.0421   162.1777   164.4944
243  O    Isotropic =   186.9439   Anisotropy =   142.7615
   XX=   135.0586   YX=   -25.4617   ZX=    59.9053
   XY=    21.1248   YY=   170.5568   ZY=   -26.0618
   XZ=    54.2196   YZ=   -18.3390   ZZ=   255.2163
   Eigenvalues:   111.5909   167.1225   282.1182
244  O    Isotropic =   189.3976   Anisotropy =   143.3257
   XX=   244.6135   YX=    36.9092   ZX=    39.6073
   XY=    26.9539   YY=   134.2250   ZY=    45.5419
   XZ=    55.6695   YZ=    19.5097   ZZ=   189.3543
   Eigenvalues:   118.2568   164.9880   284.9481
245  O    Isotropic =   190.2185   Anisotropy =   139.8592
   XX=   243.9510   YX=    51.9908   ZX=     7.4301
   XY=    59.0052   YY=   203.1832   ZY=   -29.6116
   XZ=   -10.0243   YZ=    -5.2050   ZZ=   123.5211
   Eigenvalues:   118.7699   168.4276   283.4579
246  O    Isotropic =   189.8282   Anisotropy =   139.6033
   XX=   135.3647   YX=    26.9296   ZX=   -20.8176
   XY=    47.4869   YY=   215.2204   ZY=   -59.2230
   XZ=     1.3144   YZ=   -57.3986   ZZ=   218.8995
   Eigenvalues:   118.5668   168.0207   282.8971
247  O    Isotropic =   190.5245   Anisotropy =   121.7121
   XX=   168.1939   YX=    -9.9858   ZX=   -30.9856
   XY=   -34.1718   YY=   149.9301   ZY=   -30.8938
   XZ=   -44.0851   YZ=   -31.5287   ZZ=   253.4494
   Eigenvalues:   118.5590   181.3485   271.6659
248  O    Isotropic =   190.5531   Anisotropy =   120.1996
   XX=   263.8493   YX=    11.3873   ZX=   -28.8560
   XY=     6.6066   YY=   128.8297   ZY=    34.1792
```

```
   XZ=   -20.9583   YZ=     8.9376   ZZ=   178.9804
   Eigenvalues:   118.7358   182.2374   270.6862
249  O    Isotropic =   186.3435   Anisotropy =   143.2516
   XX=   253.1358   YX=   -14.0542   ZX=    53.7303
   XY=    -7.6156   YY=   167.0744   ZY=   -26.5666
   XZ=    71.2518   YZ=    18.0725   ZZ=   138.8204
   Eigenvalues:   111.2854   165.9006   281.8446
250  O    Isotropic =   189.1599   Anisotropy =   143.5725
   XX=   169.2079   YX=   -35.4809   ZX=    39.2182
   XY=   -56.9575   YY=   170.9229   ZY=   -56.2614
   XZ=    15.6953   YZ=   -61.3730   ZZ=   227.3490
   Eigenvalues:   117.3636   165.2412   284.8749
251  C    Isotropic =    50.4828   Anisotropy =   134.2493
   XX=   106.6563   YX=    51.2833   ZX=    45.5082
   XY=    44.4466   YY=     5.0810   ZY=   -24.7356
   XZ=    54.3341   YZ=   -36.5334   ZZ=    39.7110
   Eigenvalues:   -43.0566    54.5226   139.9824
252  C    Isotropic =    50.6868   Anisotropy =   134.4348
   XX=   119.0413   YX=    44.4966   ZX=    31.8922
   XY=    38.9565   YY=   -32.8844   ZY=   -18.5098
   XZ=    28.7729   YZ=     6.1643   ZZ=    65.9035
   Eigenvalues:   -45.3015    57.0519   140.3100
253  N    Isotropic =   -57.2289   Anisotropy =   477.2527
   XX=   192.1431   YX=   144.6305   ZX=   128.7980
   XY=   118.3042   YY=  -249.9276   ZY=   -82.5786
   XZ=   138.2853   YZ=   -81.2808   ZZ=  -113.9021
   Eigenvalues:  -347.3537   -85.2724   260.9396
254  C    Isotropic =    38.4799   Anisotropy =    96.5537
   XX=    91.4581   YX=    14.5573   ZX=    36.4158
   XY=    20.1193   YY=    35.5620   ZY=   -34.6780
   XZ=    28.7407   YZ=    -5.5307   ZZ=   -11.5804
   Eigenvalues:   -30.0258    42.6164   102.8490
255  C    Isotropic =    39.2760   Anisotropy =    97.9365
   XX=    98.5393   YX=     3.3621   ZX=     8.0779
   XY=    19.0081   YY=   -17.8884   ZY=    45.1365
   XZ=    22.3614   YZ=     6.9228   ZZ=    37.1772
   Eigenvalues:   -28.4302    41.6913   104.5671
256  N    Isotropic =   -45.8561   Anisotropy =   470.3946
   XX=   188.5192   YX=   112.5208   ZX=   107.1507
   XY=   161.6462   YY=  -279.9030   ZY=   -25.6847
   XZ=   140.6376   YZ=   -16.8021   ZZ=   -46.1845
   Eigenvalues:  -327.9173   -77.3912   267.7403
257  N    Isotropic =   -56.8696   Anisotropy =   476.6778
   XX=   -98.1861   YX=    35.2930   ZX=   100.6784
   XY=    36.5023   YY=  -181.3283   ZY=  -236.0192
   XZ=    86.8375   YZ=  -255.7672   ZZ=   108.9056
   Eigenvalues:  -346.6870   -84.8374   260.9156
258  C    Isotropic =    50.6126   Anisotropy =   134.6876
   XX=    61.3872   YX=   -26.6845   ZX=     9.7380
   XY=    -4.3033   YY=    -0.6363   ZY=   -73.7581
   XZ=    18.5893   YZ=   -81.3803   ZZ=    91.0871
   Eigenvalues:   -45.2842    56.7178   140.4043
259  C    Isotropic =    50.3152   Anisotropy =   134.6666
   XX=    45.4338   YX=    20.9090   ZX=    45.1435
```

```
   XY=    11.4135   YY=    25.0821   ZY=   -74.1578
   XZ=    32.3794   YZ=   -79.3007   ZZ=    80.4296
   Eigenvalues:   -43.4581    54.3107   140.0929
260  N    Isotropic =   -46.9632   Anisotropy =   470.8809
   XX=   -59.5575   YX=   -42.6190   ZX=    78.8726
   XY=   -26.5112   YY=  -183.3171   ZY=  -277.1619
   XZ=    62.0720   YZ=  -225.1645   ZZ=   101.9852
   Eigenvalues:  -329.6324   -78.2145   266.9575
261  C    Isotropic =    39.2218   Anisotropy =    98.0219
   XX=    22.8684   YX=   -18.4196   ZX=     9.2177
   XY=   -51.5547   YY=     7.8610   ZY=   -47.7261
   XZ=    -9.8042   YZ=   -28.4475   ZZ=    86.9360
   Eigenvalues:   -28.6063    41.7020   104.5697
262  C    Isotropic =    38.4740   Anisotropy =    96.3127
   XX=    -4.2472   YX=    10.0579   ZX=    21.2847
   XY=    37.1159   YY=    38.6381   ZY=   -36.9489
   XZ=    35.6473   YZ=   -33.1629   ZZ=    81.0310
   Eigenvalues:   -29.9846    42.7241   102.6824
263  N    Isotropic =   -56.9030   Anisotropy =   477.8121
   XX=   220.3835   YX=   143.0703   ZX=    79.2812
   XY=   120.3592   YY=  -236.0163   ZY=   -97.1710
   XZ=    75.1418   YZ=  -106.0621   ZZ=  -155.0761
   Eigenvalues:  -345.9369   -86.4104   261.6385
264  C    Isotropic =    37.7549   Anisotropy =    97.1625
   XX=    93.9299   YX=    20.7595   ZX=    28.6303
   XY=    19.0263   YY=    37.6209   ZY=   -34.3929
   XZ=    17.3549   YZ=    -5.0056   ZZ=   -18.2861
   Eigenvalues:   -30.8778    41.6126   102.5299
265  C    Isotropic =    38.3722   Anisotropy =    95.9357
   XX=    94.3661   YX=    33.7994   ZX=    -0.4925
   XY=    23.4157   YY=   -15.4356   ZY=    37.4187
   XZ=     5.0738   YZ=     8.7911   ZZ=    36.1861
   Eigenvalues:   -29.7627    42.5500   102.3293
266  N    Isotropic =   -54.1085   Anisotropy =   474.2049
   XX=   221.2904   YX=   145.4126   ZX=    66.7309
   XY=   131.0232   YY=  -283.3881   ZY=   -62.2577
   XZ=    52.2625   YZ=   -51.2714   ZZ=  -100.2278
   Eigenvalues:  -339.3769   -84.9768   262.0281
267  C    Isotropic =    49.9599   Anisotropy =   134.4942
   XX=   129.5226   YX=    34.6484   ZX=    18.8454
   XY=    34.6544   YY=     4.4874   ZY=   -37.1924
   XZ=    26.5754   YZ=   -49.8925   ZZ=    15.8697
   Eigenvalues:   -43.4813    53.7383   139.6227
268  C    Isotropic =    49.4963   Anisotropy =   135.8777
   XX=   130.0187   YX=    35.9364   ZX=    17.5532
   XY=    42.0556   YY=   -35.3038   ZY=   -26.7168
   XZ=    11.6494   YZ=    -6.9488   ZZ=    53.7741
   Eigenvalues:   -47.8499    56.2575   140.0815
269  N    Isotropic =   -56.6271   Anisotropy =   477.1083
   XX=  -121.0834   YX=    78.8639   ZX=    52.9712
   XY=    69.7744   YY=  -197.1703   ZY=  -215.4305
   XZ=    49.8078   YZ=  -238.8545   ZZ=   148.3724
   Eigenvalues:  -345.4568   -85.8696   261.4451
270  C    Isotropic =    37.9717   Anisotropy =    96.9328
```

```
   XX=    -9.0470   YX=    13.6104   ZX=    11.3307
   XY=    39.7767   YY=    39.3534   ZY=   -31.6321
   XZ=    27.6563   YZ=   -37.2182   ZZ=    83.6087
   Eigenvalues:   -30.3023    41.6239   102.5936
271  C    Isotropic =    38.2140   Anisotropy =    96.3491
   XX=    24.4883   YX=   -15.2478   ZX=    -5.6199
   XY=   -41.5249   YY=    11.8444   ZY=   -43.1460
   XZ=   -18.4823   YZ=   -50.1972   ZZ=    78.3092
   Eigenvalues:   -29.6873    41.8825   102.4467
272  N    Isotropic =   -54.2843   Anisotropy =   474.7054
   XX=   -86.8211   YX=    13.7771   ZX=    14.1148
   XY=    21.6862   YY=  -220.0599   ZY=  -229.7308
   XZ=    26.6067   YZ=  -246.9027   ZZ=   144.0283
   Eigenvalues:  -340.3584   -84.6804   262.1860
273  C    Isotropic =    49.7370   Anisotropy =   134.3832
   XX=    31.5388   YX=    41.7856   ZX=    23.8498
   XY=    31.1213   YY=     7.8040   ZY=   -63.3812
   XZ=    14.4898   YZ=   -61.0263   ZZ=   109.8683
   Eigenvalues:   -44.0338    53.9190   139.3258
274  C    Isotropic =    49.6838   Anisotropy =   135.7745
   XX=    56.8791   YX=   -10.3440   ZX=    -2.0564
   XY=     7.2157   YY=   -15.4289   ZY=   -72.7859
   XZ=     8.0941   YZ=   -69.3030   ZZ=   107.6012
   Eigenvalues:   -47.8897    56.7409   140.2001
275  C    Isotropic =    30.8939   Anisotropy =    95.2522
   XX=    75.2799   YX=    25.2284   ZX=    24.7125
   XY=    31.9058   YY=   -33.2072   ZY=    -5.7520
   XZ=    23.5608   YZ=     0.8442   ZZ=    50.6090
   Eigenvalues:   -41.0344    39.3206    94.3953
276  C    Isotropic =    35.7387   Anisotropy =    82.1765
   XX=    65.4070   YX=    36.4544   ZX=    38.4689
   XY=    29.4538   YY=    15.1250   ZY=   -35.6173
   XZ=    40.0405   YZ=   -49.5085   ZZ=    26.6841
   Eigenvalues:   -45.3290    62.0221    90.5231
277  C    Isotropic =    35.3905   Anisotropy =    82.9317
   XX=    39.0942   YX=    36.9286   ZX=    40.1521
   XY=    24.0568   YY=    17.6503   ZY=   -48.0862
   XZ=    33.8667   YZ=   -52.5354   ZZ=    49.4271
   Eigenvalues:   -45.1764    60.6697    90.6783
278  C    Isotropic =    31.0393   Anisotropy =    94.9890
   XX=    45.8473   YX=   -19.2013   ZX=     9.7311
   XY=   -12.5505   YY=    -9.5235   ZY=   -57.8941
   XZ=    13.3342   YZ=   -51.8190   ZZ=    56.7941
   Eigenvalues:   -41.2514    40.0041    94.3653
279  C    Isotropic =    30.6091   Anisotropy =    97.5870
   XX=    88.4296   YX=    28.5795   ZX=     9.7435
   XY=    28.6505   YY=   -33.2185   ZY=   -14.9197
   XZ=     9.1913   YZ=    -5.7197   ZZ=    36.6162
   Eigenvalues:   -41.5073    37.6675    95.6671
280  C    Isotropic =    34.8090   Anisotropy =    81.8639
   XX=    82.3550   YX=    22.2495   ZX=    18.7748
   XY=    21.3901   YY=    15.6848   ZY=   -41.7531
   XZ=    21.2986   YZ=   -57.7511   ZZ=     6.3873
   Eigenvalues:   -45.7332    60.7753    89.3850
```

```
281  C    Isotropic =    30.7094   Anisotropy =    97.5748
   XX=    37.7890   YX=    -8.5724   ZX=    -0.6357
   XY=     0.7033   YY=   -17.9513   ZY=   -51.4323
   XZ=     2.0441   YZ=   -51.6498   ZZ=    72.2905
   Eigenvalues:   -41.4681    37.8370    95.7592
282  C    Isotropic =    34.3308   Anisotropy =    83.0802
   XX=    24.6116   YX=    51.6321   ZX=    24.8880
   XY=    36.5895   YY=     7.4651   ZY=   -39.3202
   XZ=    19.0634   YZ=   -39.2905   ZZ=    70.9157
   Eigenvalues:   -45.8047    59.0795    89.7176
283  O    Isotropic =   -80.9428   Anisotropy =   669.3700
   XX=   332.7108   YX=   100.7254   ZX=    88.7750
   XY=    99.9564   YY=  -135.8123   ZY=    62.3946
   XZ=    88.0490   YZ=    52.4372   ZZ=  -439.7269
   Eigenvalues:  -456.5537  -151.5786   365.3039
284  O    Isotropic =  -137.9553   Anisotropy =   761.3251
   XX=   334.8147   YX=   138.2357   ZX=    41.5685
   XY=   137.8340   YY=  -363.8817   ZY=   193.9844
   XZ=    41.9583   YZ=   197.6683   ZZ=  -384.7991
   Eigenvalues:  -575.2611  -208.1996   369.5947
285  O    Isotropic =   -86.6867   Anisotropy =   681.3161
   XX=  -369.8426   YX=   -93.6219   ZX=   246.7676
   XY=  -107.0130   YY=   -31.9111   ZY=  -200.9121
   XZ=   250.5372   YZ=  -199.5088   ZZ=   141.6936
   Eigenvalues:  -471.6302  -155.9540   367.5240
286  N    Isotropic =    87.3043   Anisotropy =   109.1620
   XX=    67.5882   YX=    -7.3373   ZX=    29.1407
   XY=   -10.3546   YY=    72.0343   ZY=   -44.0811
   XZ=    32.1195   YZ=   -48.2554   ZZ=   122.2904
   Eigenvalues:    40.9767    60.8573   160.0789
287  O    Isotropic =  -139.9314   Anisotropy =   767.3270
   XX=  -411.7741   YX=  -256.2667   ZX=   152.2171
   XY=  -250.6581   YY=  -206.8955   ZY=  -220.4670
   XZ=   149.3407   YZ=  -216.2330   ZZ=   198.8755
   Eigenvalues:  -582.7285  -208.6855   371.6200
288  O    Isotropic =   -83.4660   Anisotropy =   675.9819
   XX=   179.9668   YX=    68.0941   ZX=   328.7595
   XY=    69.1624   YY=  -139.8068   ZY=    96.1978
   XZ=   322.5341   YZ=    84.6135   ZZ=  -290.5580
   Eigenvalues:  -464.8086  -152.7779   367.1886
289  O    Isotropic =  -142.9333   Anisotropy =   770.6682
   XX=   236.3814   YX=    22.9916   ZX=   270.9574
   XY=    26.0514   YY=  -385.6826   ZY=   214.6665
   XZ=   275.6706   YZ=   219.6737   ZZ=  -279.4987
   Eigenvalues:  -587.4839  -212.1615   370.8455
290  O    Isotropic =   -82.6022   Anisotropy =   671.7886
   XX=  -457.2549   YX=    10.9952   ZX=    37.5588
   XY=     0.8112   YY=   -63.6325   ZY=  -197.1655
   XZ=    35.3345   YZ=  -197.4990   ZZ=   273.0807
   Eigenvalues:  -459.7876  -153.2759   365.2568
291  O    Isotropic =  -134.1912   Anisotropy =   756.2705
   XX=  -455.3811   YX=  -163.0514   ZX=   -52.0152
   XY=  -158.6548   YY=  -199.9095   ZY=  -257.7067
   XZ=   -52.8196   YZ=  -258.1599   ZZ=   252.7171
```

```
   Eigenvalues:  -568.1947  -204.3679   369.9891
292  H    Isotropic =    24.9377   Anisotropy =     8.3789
   XX=    26.4488   YX=     2.5823   ZX=    -2.8453
   XY=     1.7720   YY=    23.8367   ZY=    -3.4384
   XZ=    -3.4833   YZ=    -2.0230   ZZ=    24.5278
   Eigenvalues:    21.3455    22.9440    30.5237
293  H    Isotropic =    23.8875   Anisotropy =     7.4513
   XX=    25.0930   YX=     0.8854   ZX=    -5.0903
   XY=     0.9362   YY=    24.7979   ZY=    -1.8314
   XZ=    -3.6455   YZ=    -1.6104   ZZ=    21.7716
   Eigenvalues:    18.6213    24.1862    28.8550
294  H    Isotropic =    24.8728   Anisotropy =     8.7524
   XX=    26.5136   YX=    -0.5927   ZX=     3.9909
   XY=    -1.6654   YY=    21.3455   ZY=    -0.4184
   XZ=     3.6939   YZ=    -1.4526   ZZ=    26.7593
   Eigenvalues:    21.1038    22.8068    30.7078
295  H    Isotropic =    23.9456   Anisotropy =     7.6642
   XX=    25.0039   YX=    -2.9967   ZX=     3.2276
   XY=    -2.4997   YY=    20.7826   ZY=     0.6607
   XZ=     3.4669   YZ=     1.4049   ZZ=    26.0502
   Eigenvalues:    18.5588    24.2229    29.0550
296  H    Isotropic =    24.3773   Anisotropy =     7.4822
   XX=    24.4089   YX=    -0.0767   ZX=     5.0016
   XY=    -1.1280   YY=    24.1741   ZY=    -0.0595
   XZ=     4.5109   YZ=    -1.0580   ZZ=    24.5489
   Eigenvalues:    19.7218    24.0446    29.3654
297  H    Isotropic =    24.9287   Anisotropy =     8.9545
   XX=    26.2487   YX=    -0.7938   ZX=     4.4615
   XY=     0.4574   YY=    22.4715   ZY=    -2.3506
   XZ=     4.6009   YZ=    -1.0680   ZZ=    26.0659
   Eigenvalues:    20.8077    23.0800    30.8984
298  H    Isotropic =    24.9405   Anisotropy =     8.2717
   XX=    25.9405   YX=     2.5024   ZX=    -3.3007
   XY=     3.6020   YY=    23.4785   ZY=    -1.4103
   XZ=    -2.6299   YZ=    -2.3912   ZZ=    25.4026
   Eigenvalues:    21.4151    22.9515    30.4550
299  H    Isotropic =    23.9207   Anisotropy =     7.4630
   XX=    23.1704   YX=     2.6479   ZX=    -3.3961
   XY=     3.3065   YY=    23.8608   ZY=    -0.2563
   XZ=    -4.6776   YZ=     0.1652   ZZ=    24.7307
   Eigenvalues:    18.7483    24.1178    28.8960
300  H    Isotropic =    27.1287   Anisotropy =     3.6014
   XX=    24.8562   YX=    -0.2153   ZX=    -0.1668
   XY=    -0.1643   YY=    27.6712   ZY=    -0.5433
   XZ=    -0.4816   YZ=    -1.6741   ZZ=    28.8586
   Eigenvalues:    24.8001    27.0563    29.5296
301  H    Isotropic =    28.9237   Anisotropy =     6.8514
   XX=    27.3882   YX=    -1.1932   ZX=    -0.3643
   XY=    -0.6363   YY=    32.0120   ZY=     1.0665
   XZ=    -1.0587   YZ=     4.4136   ZZ=    27.3709
   Eigenvalues:    26.0426    27.2371    33.4913
302  H    Isotropic =    28.9315   Anisotropy =     6.7024
   XX=    26.6142   YX=     0.1378   ZX=    -0.0103
   XY=    -2.9201   YY=    31.9227   ZY=     2.3493
```

```
   XZ=    -1.4145   YZ=     2.2736   ZZ=    28.2575
   Eigenvalues:    26.2530    27.1417    33.3997
303  H    Isotropic =    27.0176   Anisotropy =     3.3986
   XX=    29.2635   YX=    -0.1920   ZX=    -0.1479
   XY=     0.5947   YY=    26.6685   ZY=     0.9882
   XZ=     0.2419   YZ=     1.1142   ZZ=    25.1208
   Eigenvalues:    24.5888    27.1806    29.2834
304  H    Isotropic =    28.9167   Anisotropy =     6.6700
   XX=    27.5069   YX=    -1.1548   ZX=    -1.0428
   XY=    -1.5432   YY=    31.8802   ZY=     4.1255
   XZ=    -0.2928   YZ=     0.8378   ZZ=    27.3630
   Eigenvalues:    26.2613    27.1254    33.3633
305  H    Isotropic =    26.9797   Anisotropy =     3.5669
   XX=    24.6201   YX=    -0.7685   ZX=    -0.2786
   XY=    -0.5022   YY=    27.2039   ZY=    -1.1571
   XZ=    -0.6166   YZ=    -0.2500   ZZ=    29.1152
   Eigenvalues:    24.3954    27.1862    29.3577
306  H    Isotropic =    27.1016   Anisotropy =     3.5995
   XX=    29.2651   YX=     1.3612   ZX=     0.0595
   XY=     0.0823   YY=    27.0888   ZY=     0.7349
   XZ=     0.1096   YZ=     0.6771   ZZ=    24.9508
   Eigenvalues:    24.7350    27.0685    29.5012
307  H    Isotropic =    28.9018   Anisotropy =     6.8614
   XX=    26.5692   YX=    -2.9725   ZX=    -1.5245
   XY=     0.1454   YY=    32.2830   ZY=     1.9176
   XZ=    -0.1455   YZ=     2.2087   ZZ=    27.8534
   Eigenvalues:    26.1195    27.1099    33.4761
308  H    Isotropic =    21.2049   Anisotropy =    19.5911
   XX=    20.2427   YX=    -3.8737   ZX=    -3.9333
   XY=    -3.2427   YY=    28.0988   ZY=     8.1065
   XZ=    -3.9753   YZ=     9.2884   ZZ=    15.2732
   Eigenvalues:    10.4527    18.8964    34.2656
309  N    Isotropic =    88.0061   Anisotropy =   106.8567
   XX=   134.5168   YX=    22.1129   ZX=    44.1321
   XY=    19.6468   YY=    54.7520   ZY=    -0.0337
   XZ=    39.8988   YZ=    -3.4507   ZZ=    74.7496
   Eigenvalues:    41.4055    63.3689   159.2439
310  H    Isotropic =    21.4802   Anisotropy =    18.9652
   XX=    13.4599   YX=    -5.3926   ZX=    -5.0021
   XY=    -4.3161   YY=    28.9132   ZY=     5.3092
   XZ=    -4.5497   YZ=     5.8001   ZZ=    22.0674
   Eigenvalues:    10.9997    19.3171    34.1236
311  N    Isotropic =    88.5141   Anisotropy =   104.0504
   XX=   152.0435   YX=    22.6097   ZX=    13.9547
   XY=    20.4442   YY=    54.8132   ZY=    -8.3337
   XZ=    13.1625   YZ=   -11.5796   ZZ=    58.6856
   Eigenvalues:    40.8581    66.8033   157.8810
312  H    Isotropic =    21.5288   Anisotropy =    16.6425
   XX=    12.0988   YX=    -0.8264   ZX=    -0.9825
   XY=    -1.0578   YY=    28.4099   ZY=     5.7072
   XZ=    -0.6670   YZ=     6.1342   ZZ=    24.0777
   Eigenvalues:    12.0210    19.9416    32.6238
313  N    Isotropic =    88.7035   Anisotropy =   103.6433
   XX=    62.8231   YX=     9.5283   ZX=     6.9242
```

```
   XY=     6.2455   YY=    63.8309   ZY=   -40.1349
   XZ=     6.8779   YZ=   -42.6153   ZZ=   139.4565
   Eigenvalues:    40.9937    67.3178   157.7991
314  H    Isotropic =    21.5550   Anisotropy =    16.7545
   XX=    21.9560   YX=    -4.8075   ZX=    -1.0473
   XY=    -4.3130   YY=    29.5998   ZY=     4.5082
   XZ=    -1.1980   YZ=     4.1900   ZZ=    13.1092
   Eigenvalues:    12.0325    19.9079    32.7247
```

cartesians coordinates

```
0 1
 H                  4.71253100    4.56614900    6.72987500
 C                  4.90157000    4.52230100    5.65491600
 C                  4.50707000    5.83817400    4.98914400
 C                  4.01119100    3.47231800    4.99109500
 H                  5.95624200    4.30351400    5.47374600
 H                  5.08009000    6.68156800    5.38048700
 H                  4.19741300    2.47152500    5.38660200
 N                  4.32629000    3.44675200    3.56568700
 N                  4.80077500    5.73216800    3.56316700
 H                  4.77844600    6.59323200    3.01494700
 N                  3.09890100    6.09184200    5.28457900
 H                  2.73862700    7.02572900    5.08328800
 N                  2.61950100    3.80873900    5.28693100
 H                  1.90091800    3.07383400    5.28129000
 C                  2.16573200    5.09087200    5.30998300
 C                  4.59527100    4.57634700    2.85651600
 O                  4.69480700    4.55536700    1.60726800
 O                  0.94119500    5.34933400    5.39646100
 H                  4.16533600    2.58999300    3.01785300
 H                 -5.23286600   -0.64028300   -1.77136900
 C                 -4.18509500   -0.87146600   -1.97530500
 C                 -3.79462800   -2.21514100   -1.35929200
 C                 -3.27457600    0.14959200   -1.29195400
 H                 -4.01520200   -0.87975400   -3.05427400
 H                 -4.37601600   -3.04773400   -1.76055800
 H                 -3.44931900    1.16259300   -1.66103600
 N                 -1.88584800   -0.20293400   -1.59002200
 N                 -2.39168900   -2.47044500   -1.68617100
 H                 -2.08509600   -3.41507900   -1.90786700
 N                 -4.04585500   -2.14608800    0.07787600
 H                 -4.21635300   -3.00640400    0.60363900
 N                 -3.57456700    0.13145300    0.13628600
 H                 -3.45346000    0.97823100    0.69824700
 C                 -3.85802900   -1.01295000    0.81445600
 C                 -1.45629800   -1.48101700   -1.74768300
 O                 -0.24262700   -1.73667400   -1.95592300
 O                 -3.97319100   -1.02071000    2.06653100
 H                 -1.16187100    0.51982100   -1.64681000
 H                 -3.11609600    2.49883900    6.82626400
```

C	-3.34931700	2.54316900	5.76000600
C	-2.96329600	1.23019400	5.07591400
C	-2.49888100	3.60522200	5.06284800
H	-4.41256600	2.75338500	5.62325100
H	-3.52013400	0.38338900	5.48435800
H	-2.67113700	4.60889100	5.45643000
N	-2.87764000	3.62362300	3.65055400
N	-3.30002800	1.33369100	3.65852800
H	-3.49482400	0.48863000	3.10768100
N	-1.54923200	0.98826400	5.32953200
H	-1.19004200	0.02751300	5.30171500
N	-1.09442300	3.27255100	5.28309500
H	-0.38646800	4.01172500	5.26054000
C	-0.62663100	1.99227100	5.32648000
C	-3.20898800	2.49723200	2.95894500
O	-3.44371600	2.52765100	1.72655100
O	0.60167800	1.74673200	5.39839500
H	-2.91691100	4.50610700	3.14372500
H	4.01520200	0.87975400	-3.05427400
C	4.18509500	0.87146600	-1.97530500
C	3.27457600	-0.14959200	-1.29195400
C	3.79462800	2.21514100	-1.35929200
H	5.23286600	0.64028300	-1.77136900
H	3.44931900	-1.16259300	-1.66103600
H	4.37601600	3.04773400	-1.76055800
N	4.04585500	2.14608800	0.07787600
N	3.57456700	-0.13145300	0.13628600
H	3.45346000	-0.97823100	0.69824700
N	1.88584800	0.20293400	-1.59002200
H	1.16187100	-0.51982100	-1.64681000
N	2.39168900	2.47044500	-1.68617100
H	2.08509600	3.41507900	-1.90786700
C	1.45629800	1.48101700	-1.74768300
C	3.85802900	1.01295000	0.81445600
O	3.97319100	1.02071000	2.06653100
O	0.24262700	1.73667400	-1.95592300
H	4.21635300	3.00640400	0.60363900
H	4.41256600	-2.75338500	5.62325100
C	3.34931700	-2.54316900	5.76000600
C	2.49888100	-3.60522200	5.06284800
C	2.96329600	-1.23019400	5.07591400
H	3.11609600	-2.49883900	6.82626400
H	2.67113700	-4.60889100	5.45643000
H	3.52013400	-0.38338900	5.48435800
N	1.54923200	-0.98826400	5.32953200
N	1.09442300	-3.27255100	5.28309500
H	0.38646800	-4.01172500	5.26054000
N	2.87764000	-3.62362300	3.65055400
H	2.91691100	-4.50610700	3.14372500
N	3.30002800	-1.33369100	3.65852800
H	3.49482400	-0.48863000	3.10768100
C	3.20898800	-2.49723200	2.95894500
C	0.62663100	-1.99227100	5.32648000
O	-0.60167800	-1.74673200	5.39839500

O	3.44371600	-2.52765100	1.72655100
H	1.19004200	-0.02751300	5.30171500
H	-5.95624200	-4.30351400	5.47374600
C	-4.90157000	-4.52230100	5.65491600
C	-4.01119100	-3.47231800	4.99109500
C	-4.50707000	-5.83817400	4.98914400
H	-4.71253100	-4.56614900	6.72987500
H	-4.19741300	-2.47152500	5.38660200
H	-5.08009000	-6.68156800	5.38048700
N	-3.09890100	-6.09184200	5.28457900
N	-2.61950100	-3.80873900	5.28693100
H	-1.90091800	-3.07383400	5.28129000
N	-4.32629000	-3.44675200	3.56568700
H	-4.16533600	-2.58999300	3.01785300
N	-4.80077500	-5.73216800	3.56316700
H	-4.77844600	-6.59323200	3.01494700
C	-4.59527100	-4.57634700	2.85651600
C	-2.16573200	-5.09087200	5.30998300
O	-0.94119500	-5.34933400	5.39646100
O	-4.69480700	-4.55536700	1.60726800
H	-2.73862700	-7.02572900	5.08328800
C	-3.80287300	13.11782300	-0.94213400
C	-3.09654700	13.23581000	0.26191700
C	-3.41156200	12.32606600	1.27915000
C	-4.36241700	11.32349900	1.11100700
C	-5.00132100	11.22732900	-0.11987300
C	-4.75774600	12.12183200	-1.16876700
C	-3.14518700	10.65020200	-5.21289600
C	-3.82782400	11.47781600	-4.31504300
C	-4.85680300	11.03535900	-3.47609200
C	-5.22919000	9.68884300	-3.59125900
C	-4.60282800	8.83012000	-4.49129000
C	-3.55876200	9.31208500	-5.27651500
C	1.48516100	12.69514600	0.59646900
C	0.22837500	13.17187900	0.96048000
C	-0.63048200	13.78785000	0.04112000
C	-0.17232400	13.90213900	-1.27591800
C	1.06834400	13.41611900	-1.70225600
C	1.88012900	12.81120900	-0.73392900
C	1.85007400	11.17829800	-4.10902000
C	1.05449500	12.32509100	-3.99173000
C	-0.18286700	12.31374500	-4.64826800
C	-0.65494200	11.20754600	-5.36161700
C	0.17660700	10.08234900	-5.41008900
C	1.43372100	10.05438000	-4.81686900
C	-2.01281000	14.29078400	0.43707100
C	-5.50533800	11.99919900	-2.49076500
C	-2.00940400	11.21150600	-6.05900800
C	1.49338800	13.52317700	-3.16113900
O	-2.79190400	12.48948700	2.52484100
O	-5.98232000	10.25675100	-0.33634700
O	-6.27786300	9.18519800	-2.80110500
O	-2.92872100	8.43203100	-6.17299400
O	-0.17660700	13.10825600	2.30101000

O	3.15725800	12.38284700	-1.11911700
O	3.13308500	11.20592200	-3.54714500
O	-0.28226500	8.98893400	-6.14917700
C	3.95269100	8.48043700	-1.51296200
C	3.96842100	9.09194600	-0.26699400
N	3.69669800	10.38443400	-0.07318700
C	3.40691600	11.04509700	-1.18290600
C	3.39377000	10.42077600	-2.46597800
N	3.66735900	9.13600200	-2.64099000
N	0.45937700	11.08887300	3.24690000
C	0.01648700	9.94682800	3.77744300
C	-1.32774400	9.61769900	3.88385700
N	-2.30877400	10.42366400	3.46928200
C	-1.88590800	11.56216200	2.93892600
C	-0.50253400	11.89404200	2.82479100
N	-5.04728800	8.29513200	0.48648900
C	-5.60700200	8.96269500	-0.50944400
C	-5.87319000	8.37086400	-1.77867600
N	-5.71588100	7.07630900	-2.00530000
C	-5.21587700	6.39714800	-0.96873200
C	-4.83871800	7.00444000	0.22097800
N	0.16966700	7.15230600	-4.80039700
C	-0.69919800	7.87857700	-5.48526000
C	-2.07835200	7.53409400	-5.58689900
N	-2.56302100	6.39593200	-5.11529600
C	-1.66223500	5.63866000	-4.48246000
C	-0.34629900	6.03452800	-4.28693300
C	4.29185000	7.01793900	-1.33020800
C	4.31872000	8.05835000	0.76418800
C	0.82800000	8.81239100	4.33280900
C	-1.43372100	8.24636800	4.51265100
C	-4.90258400	4.92298600	-0.86937300
C	-4.18034400	5.97184700	1.09199500
C	-1.85636500	4.29445600	-3.82062500
C	0.33213500	5.01034300	-3.42060200
O	-5.12678200	4.03618800	-1.65835100
O	-3.64714300	6.12630500	2.17703300
O	-2.41638800	7.59688700	4.78448200
N	-0.10150200	7.86446000	4.73282100
O	2.03985500	8.72257100	4.42402600
O	4.38983900	6.14116200	-2.15582500
O	4.43769300	8.20264300	1.96861900
O	-2.83607600	3.58692000	-3.79726600
O	1.46172600	5.03454100	-2.96473300
H	-3.59538100	13.83193000	-1.73635900
H	-4.58943500	10.63264900	1.91318400
H	-3.53295400	12.52354000	-4.25671600
H	-4.91274000	7.79428900	-4.56750500
H	2.14064300	12.24312000	1.33148900
H	-0.81597900	14.38734000	-2.00655800
H	-0.80514900	13.20475300	-4.59655700
H	2.06744300	9.17974900	-4.89330600
H	-1.99432800	14.62859700	1.47513800
H	-2.26153100	15.15476900	-0.18846300

H	-5.54910300	12.98981300	-2.95610800
H	-6.53203900	11.68186500	-2.29651800
H	-2.25770300	12.24507100	-6.32372800
H	-1.93735300	10.64284700	-6.98847600
H	2.57833000	13.63087600	-3.21790900
H	1.04712300	14.42770400	-3.58806100
H	0.19195000	6.93427500	5.08331400
N	4.48489000	6.88001900	0.05370300
H	4.66065900	5.97924600	0.53510400
N	-4.27948200	4.78781400	0.38100200
H	-3.95404200	3.89042100	0.78788700
N	-0.62045400	4.02573200	-3.21567800
H	-0.38718500	3.14922600	-2.70584100
C	0.18286700	-12.31374500	-4.64826800
C	0.65494200	-11.20754600	-5.36161700
C	-0.17660700	-10.08234900	-5.41008900
C	-1.43372100	-10.05438000	-4.81686900
C	-1.85007400	-11.17829800	-4.10902000
C	-1.05449500	-12.32509100	-3.99173000
C	0.63048200	-13.78785000	0.04112000
C	0.17232400	-13.90213900	-1.27591800
C	-1.06834400	-13.41611900	-1.70225600
C	-1.88012900	-12.81120900	-0.73392900
C	-1.48516100	-12.69514600	0.59646900
C	-0.22837500	-13.17187900	0.96048000
C	4.60282800	-8.83012000	-4.49129000
C	3.55876200	-9.31208500	-5.27651500
C	3.14518700	-10.65020200	-5.21289600
C	3.82782400	-11.47781600	-4.31504300
C	4.85680300	-11.03535900	-3.47609200
C	5.22919000	-9.68884300	-3.59125900
C	5.00132100	-11.22732900	-0.11987300
C	4.75774600	-12.12183200	-1.16876700
C	3.80287300	-13.11782300	-0.94213400
C	3.09654700	-13.23581000	0.26191700
C	3.41156200	-12.32606600	1.27915000
C	4.36241700	-11.32349900	1.11100700
C	2.00940400	-11.21150600	-6.05900800
C	-1.49338800	-13.52317700	-3.16113900
C	2.01281000	-14.29078400	0.43707100
C	5.50533800	-11.99919900	-2.49076500
O	0.28226500	-8.98893400	-6.14917700
O	-3.13308500	-11.20592200	-3.54714500
O	-3.15725800	-12.38284700	-1.11911700
O	0.17660700	-13.10825600	2.30101000
O	2.92872100	-8.43203100	-6.17299400
O	6.27786300	-9.18519800	-2.80110500
O	5.98232000	-10.25675100	-0.33634700
O	2.79190400	-12.48948700	2.52484100
C	4.83871800	-7.00444000	0.22097800
C	5.21587700	-6.39714800	-0.96873200
N	5.71588100	-7.07630900	-2.00530000
C	5.87319000	-8.37086400	-1.77867600
C	5.60700200	-8.96269500	-0.50944400

N	5.04728800	-8.29513200	0.48648900
N	2.56302100	-6.39593200	-5.11529600
C	1.66223500	-5.63866000	-4.48246000
C	0.34629900	-6.03452800	-4.28693300
N	-0.16966700	-7.15230600	-4.80039700
C	0.69919800	-7.87857700	-5.48526000
C	2.07835200	-7.53409400	-5.58689900
N	-3.66735900	-9.13600200	-2.64099000
C	-3.39377000	-10.42077600	-2.46597800
C	-3.40691600	-11.04509700	-1.18290600
N	-3.69669800	-10.38443400	-0.07318700
C	-3.96842100	-9.09194600	-0.26699400
C	-3.95269100	-8.48043700	-1.51296200
N	2.30877400	-10.42366400	3.46928200
C	1.88590800	-11.56216200	2.93892600
C	0.50253400	-11.89404200	2.82479100
N	-0.45937700	-11.08887300	3.24690000
C	-0.01648700	-9.94682800	3.77744300
C	1.32774400	-9.61769900	3.88385700
C	4.18034400	-5.97184700	1.09199500
C	4.90258400	-4.92298600	-0.86937300
C	1.85636500	-4.29445600	-3.82062500
C	-0.33213500	-5.01034300	-3.42060200
C	-4.31872000	-8.05835000	0.76418800
C	-4.29185000	-7.01793900	-1.33020800
C	-0.82800000	-8.81239100	4.33280900
C	1.43372100	-8.24636800	4.51265100
O	-4.43769300	-8.20264300	1.96861900
O	-4.38983900	-6.14116200	-2.15582500
O	-1.46172600	-5.03454100	-2.96473300
N	0.62045400	-4.02573200	-3.21567800
O	2.83607600	-3.58692000	-3.79726600
O	3.64714300	-6.12630500	2.17703300
O	5.12678200	-4.03618800	-1.65835100
O	-2.03985500	-8.72257100	4.42402600
O	2.41638800	-7.59688700	4.78448200
H	0.80514900	-13.20475300	-4.59655700
H	-2.06744300	-9.17974900	-4.89330600
H	0.81597900	-14.38734000	-2.00655800
H	-2.14064300	-12.24312000	1.33148900
H	4.91274000	-7.79428900	-4.56750500
H	3.53295400	-12.52354000	-4.25671600
H	3.59538100	-13.83193000	-1.73635900
H	4.58943500	-10.63264900	1.91318400
H	1.93735300	-10.64284700	-6.98847600
H	2.25770300	-12.24507100	-6.32372800
H	-1.04712300	-14.42770400	-3.58806100
H	-2.57833000	-13.63087600	-3.21790900
H	2.26153100	-15.15476900	-0.18846300
H	1.99432800	-14.62859700	1.47513800
H	6.53203900	-11.68186500	-2.29651800
H	5.54910300	-12.98981300	-2.95610800
H	0.38718500	-3.14922600	-2.70584100
N	4.27948200	-4.78781400	0.38100200

H	3.95404200	-3.89042100	0.78788700
N	-4.48489000	-6.88001900	0.05370300
H	-4.66065900	-5.97924600	0.53510400
N	0.10150200	-7.86446000	4.73282100
H	-0.19195000	-6.93427500	5.08331400

```
1 2 1.0
2 3 1.0 4 1.0 5 1.0
3 6 1.0 9 1.0 11 1.0
4 7 1.0 8 1.0 13 1.0
5
6
7
8 16 1.5 19 1.0
9 10 1.0 16 1.5
10
11 12 1.0 15 1.5
12
13 14 1.0 15 1.5
14
15 18 2.0
16 17 2.0
17
18
19
20 21 1.0
21 22 1.0 23 1.0 24 1.0
22 25 1.0 28 1.0 30 1.0
23 26 1.0 27 1.0 32 1.0
24
25
26
27 35 1.5 38 1.0
28 29 1.0 35 1.5
29
30 31 1.0 34 1.5
31
32 33 1.0 34 1.5
33
34 37 2.0
35 36 2.0
36
37
38
39 40 1.0
40 41 1.0 42 1.0 43 1.0
41 44 1.0 47 1.0 49 1.0
42 45 1.0 46 1.0 51 1.0
43
44
45
46 54 1.5 57 1.0
47 48 1.0 54 1.5
48
```

49 50 1.0 53 1.5
50
51 52 1.0 53 1.5
52
53 56 2.0
54 55 2.0
55
56
57
58 59 1.0
59 60 1.0 61 1.0 62 1.0
60 63 1.0 66 1.0 68 1.0
61 64 1.0 65 1.0 70 1.0
62
63
64
65 73 1.5 76 1.0
66 67 1.0 73 1.5
67
68 69 1.0 72 1.5
69
70 71 1.0 72 1.5
71
72 75 2.0
73 74 2.0
74
75
76
77 78 1.0
78 79 1.0 80 1.0 81 1.0
79 82 1.0 85 1.0 87 1.0
80 83 1.0 84 1.0 89 1.0
81
82
83
84 92 1.5 95 1.0
85 86 1.0 92 1.5
86
87 88 1.0 91 1.5
88
89 90 1.0 91 1.5
90
91 94 2.0
92 93 2.0
93
94
95
96 97 1.0
97 98 1.0 99 1.0 100 1.0
98 101 1.0 104 1.0 106 1.0
99 102 1.0 103 1.0 108 1.0
100
101
102

103 111 1.5 114 1.0
104 105 1.0 111 1.5
105
106 107 1.0 110 1.5
107
108 109 1.0 110 1.5
109
110 113 2.0
111 112 2.0
112
113
114
115 116 1.5 120 1.5 192 1.0
116 117 1.5 139 1.0
117 118 1.5 143 1.0
118 119 1.5 193 1.0
119 120 1.5 144 1.0
120 140 1.0
121 122 1.5 126 1.5 141 1.0
122 123 1.5 194 1.0
123 124 1.5 140 1.0
124 125 1.5 145 1.0
125 126 1.5 195 1.0
126 146 1.0
127 128 1.5 132 1.5 196 1.0
128 129 1.5 147 1.0
129 130 1.5 139 1.0
130 131 1.5 197 1.0
131 132 1.5 142 1.0
132 148 1.0
133 134 1.5 138 1.5 149 1.0
134 135 1.5 142 1.0
135 136 1.5 198 1.0
136 137 1.5 141 1.0
137 138 1.5 150 1.0
138 199 1.0
139 200 1.0 201 1.0
140 202 1.0 203 1.0
141 204 1.0 205 1.0
142 206 1.0 207 1.0
143 161 1.0
144 164 1.0
145 165 1.0
146 171 1.0
147 162 1.0
148 154 1.0
149 155 1.0
150 170 1.0
151 152 1.5 156 1.5 175 1.0
152 153 1.5 176 1.0
153 154 1.5
154 155 1.5
155 156 1.5
156

```
157 158 1.5 162 1.5
158 159 1.5 177 1.0
159 160 1.5 178 1.0
160 161 1.5
161 162 1.5
162
163 164 1.5 168 1.5
164 165 1.5
165 166 1.5
166 167 1.5
167 168 1.5 179 1.0
168 180 1.0
169 170 1.5 174 1.5
170 171 1.5
171 172 1.5
172 173 1.5
173 174 1.5 181 1.0
174 182 1.0
175 188 2.0 209 1.0
176 189 2.0 209 1.0
177 186 1.0 187 2.0
178 185 2.0 186 1.0
179 183 2.0 211 1.0
180 184 2.0 211 1.0
181 190 2.0 213 1.0
182 191 2.0 213 1.0
183
184
185
186 208 1.0
187
188
189
190
191
192
193
194
195
196
197
198
199
200
201
202
203
204
205
206
207
208
209 210 1.0
210
```

```
211 212 1.0
212
213 214 1.0
214
215 216 1.5 220 1.5 292 1.0
216 217 1.5 239 1.0
217 218 1.5 243 1.0
218 219 1.5 293 1.0
219 220 1.5 244 1.0
220 240 1.0
221 222 1.5 226 1.5 241 1.0
222 223 1.5 294 1.0
223 224 1.5 240 1.0
224 225 1.5 245 1.0
225 226 1.5 295 1.0
226 246 1.0
227 228 1.5 232 1.5 296 1.0
228 229 1.5 247 1.0
229 230 1.5 239 1.0
230 231 1.5 297 1.0
231 232 1.5 242 1.0
232 248 1.0
233 234 1.5 238 1.5 249 1.0
234 235 1.5 242 1.0
235 236 1.5 298 1.0
236 237 1.5 241 1.0
237 238 1.5 250 1.0
238 299 1.0
239 300 1.0 301 1.0
240 302 1.0 303 1.0
241 304 1.0 305 1.0
242 306 1.0 307 1.0
243 261 1.0
244 264 1.0
245 265 1.0
246 271 1.0
247 262 1.0
248 254 1.0
249 255 1.0
250 270 1.0
251 252 1.5 256 1.5 275 1.0
252 253 1.5 276 1.0
253 254 1.5
254 255 1.5
255 256 1.5
256
257 258 1.5 262 1.5
258 259 1.5 277 1.0
259 260 1.5 278 1.0
260 261 1.5
261 262 1.5
262
263 264 1.5 268 1.5
264 265 1.5
```

```
265 266 1.5
266 267 1.5
267 268 1.5 279 1.0
268 280 1.0
269 270 1.5 274 1.5
270 271 1.5
271 272 1.5
272 273 1.5
273 274 1.5 281 1.0
274 282 1.0
275 288 2.0 309 1.0
276 289 2.0 309 1.0
277 286 1.0 287 2.0
278 285 2.0 286 1.0
279 283 2.0 311 1.0
280 284 2.0 311 1.0
281 290 2.0 313 1.0
282 291 2.0 313 1.0
283
284
285
286 308 1.0
287
288
289
290
291
292
293
294
295
296
297
298
299
300
301
302
303
304
305
306
307
308
309 310 1.0
310
311 312 1.0
312
313 314 1.0
314
```
